# Grapevine comparative early transcriptomic profiling suggests that *Flavescence dorée* phytoplasma represses plant responses induced by vector feeding in susceptible varieties

**DOI:** 10.1186/s12864-019-5908-6

**Published:** 2019-06-26

**Authors:** Nadia Bertazzon, Paolo Bagnaresi, Vally Forte, Elisabetta Mazzucotelli, Luisa Filippin, Davide Guerra, Antonella Zechini, Luigi Cattivelli, Elisa Angelini

**Affiliations:** 1 CREA Research Centre for Viticulture and Enology, 31015 Conegliano (TV), Italy; 2 CREA Research Centre for Genomics and Bioinformatics, 29017 Fiorenzuola d’Arda (PC), Italy

**Keywords:** *Flavescence dorèe*, *Scaphoideus titanus*, *Vitis vinifera*, Grapevine, RNA-Seq, Passive defense, Active defense

## Abstract

**Background:**

*Flavescence dorée* is the most serious grapevine yellows disease in Europe. It is caused by phytoplasmas which are transmitted from grapevine to grapevine by the leafhopper *Scaphoideus titanus*. Differences in susceptibility among grapevine varieties suggest the existence of specific genetic features associated with resistance to the phytoplasma and/or possibly with its vector. In this work, RNA-Seq was used to compare early transcriptional changes occurring during the three-trophic interaction between the phytoplasma, its vector and the grapevine, represented by two different cultivars, one very susceptible to the disease and the other scarcely susceptible.

**Results:**

The comparative analysis of the constitutive transcriptomic profiles suggests the existence of passive defense strategies against the insect and/or the phytoplasma in the scarcely-susceptible cultivar. Moreover, the attack by the infective vector on the scarcely-susceptible variety prompted immediate and substantial transcriptomic changes that led to the rapid erection of further active defenses. On the other hand, in the most susceptible variety the response was delayed and mainly consisted of the induction of phytoalexin synthesis. Surprisingly, the jasmonic acid- and ethylene-mediated defense reactions, activated by the susceptible cultivar following FD-free insect feeding, were not detected in the presence of the phytoplasma-infected vector.

**Conclusions:**

The comparison of the transcriptomic response in two grapevine varieties with different levels of susceptibility to *Flavescence dorèe* highlighted both passive and active defense mechanisms against the vector and/or the pathogen in the scarcely-susceptible variety, as well as the capacity of the phytoplasmas to repress the defense reaction against the insect in the susceptible variety.

**Electronic supplementary material:**

The online version of this article (10.1186/s12864-019-5908-6) contains supplementary material, which is available to authorized users.

## Background

Phytoplasmas are plant pathogenic wall-less gram-positive bacteria associated with numerous diseases in wild and cultivated species worldwide [1]. They are phloem-obligate parasites, are very difficult to cultivate in an axenic medium [2] and are characterized by a transmission mediated by specific insect vectors or by grafting.

In grapevine, phytoplasmas cause grapevine yellows (GY) diseases, which lead to very serious damage to the grape and wine industry, ranging from a lower yield of berries to plant death. One of the most destructive GY diseases is *Flavescence dorée* (FD), a quarantine pest in the European Community ([3]; CE Directive 257/2000). FD, transmitted by the leafhopper *Scaphoideus titanus* (St) [4], is highly epidemic. It is caused by several isolates of phytoplasmas classified in the phylogenetic group 16SrV, subgroups C and D [3]. In Europe, *S. titanus* is strongly dependent on the vine, on which it completes one generation per year. The eggs are laid in the late summer and pass the winter; and hatching begins in May. From the first larval stage onwards, the insect can acquire the pathogen passively during feeding on infected plants and, after an incubation period of about one month, can transmit the phytoplasma to healthy plants throughout its remaining life [5]. The only effective strategies to limit FD epidemics are strictly preventive, and are mostly based on insecticide treatments targeting the insect vector. However, with a view to create more sustainable agriculture, they should be combined with modern approaches, such as the elicitation of grapevine defenses and/or the selection of resistant plant material.

In *Vitis vinifera*, intraspecific variability for susceptibility to GY diseases has already been observed both from field experience [6–11] and in controlled inoculation conditions [12]. In this context it is worth to note that resistance to phytoplasmas is defined as absence of symptoms and growth alteration and low titer of the pathogen, whereas tolerance is absence or mild symptoms but high titer of the pathogen. Indeed, some varieties, such as Chardonnay and Pinot gris, show very serious damage from GY, while when some others, such as Tocai friulano or Moscato bianco, are subjected to the same disease pressure, only a few plants display the symptoms, usually in just one or two branches. It is not known if the observed differences in susceptibility could be caused also by insect preference or feeding behavior. In the last few years, some molecular studies aimed at identifying the grapevine genetic determinants involved in the response to GY disease have been carried out on Chardonnay and a few other varieties in field experiments [13–18]. These analyses have been performed on healthy and infected plants grown under field conditions, thus revealing how the disease and the symptomatology modify the transcriptomic grapevine profile. Generally, the recorded differences consisted of the inhibition of genes involved in the photosynthetic chain and in the phenylpropanoid biosynthesis in susceptible varieties, and of the induction of defense genes from the metabolic pathways, leading to the formation of flavonoids, anthocyanins, antioxidants and a few PR proteins, which sometimes differ among varieties with diverse susceptibility [13–17]. The induction of genes responsible for cell wall reinforcement was observed in cultivars with both high and low susceptibility [13]. In order to take a step forward in understanding the defense mechanisms activated in scarcely-susceptible varieties, it is of paramount importance to examine the response of the plant during the early phase of infection, though this kind of analysis is very difficult in the species-specific tri-trophic interaction involving the FD phytoplasma, the grapevine and the vector. Indeed, transmission trials should be established in controlled conditions using the leafhopper *S. titanus,* which it is unfortunately not yet possible to rear for the whole life cycle. Moreover, as FD is transmitted by an insect vector, the scarce susceptibility observed in some grapevine varieties could be a combined plant response to both the phytoplasma and the vector.

In the present work a focused experiment aimed at distinguishing the above effects was set up in controlled conditions on two grapevine varieties which display very different susceptibility to FD. The total mRNA expression was evaluated in two early infection stages in order to identify the quantitative and qualitative transcriptomic differences putatively associated with the diverse plant susceptibility to FD and its vector.

## Results

### Transmission trial

All *S. titanus* specimens used in the experiments were analyzed singularly by real-time PCR for the presence of FD phytoplasma. The analysis confirmed the absence of the pathogen in all the insects used in HSt treatments. On the other hand, the phytoplasma was found in 16 *S. titanus* specimens fed on FD-infected leaves, which corresponds to 28% of the insects collected from FDSt treatments.

All the plantlets used in noSt and HSt treatments and analyzed by real-time PCR proved to be negative for the presence of the FD phytoplasma. The plantlets of the FDSt treatments where at least one of the two insects had been found to be infected were analyzed. In total, ten micropropagated plantlets were infected (six out of 11 in Chardonnay; four out of 19 in T. friulano; no statistically significant differences). The quantification of the FD phytoplasma gave different 16S rRNA copy numbers, varying from 1.2 × 10^3^ to 3.7 × 10^4^ for μg of RNA, without any significant differences between the two varieties and the two time points (Additional file 1).

### Selection of biological replicates for RNA-Seq analyses

Some plantlets used for the transmission trials were discarded due either to absence of FD phytoplasma in the FDSt conditions or to poor quality of the RNA extracted (RIN < 7) (Additional file 2). A further selection of the samples was performed after sequencing and calculation of the expression values following statistic criteria including Pearson correlation coefficients and sample clustering of rlog transformed data (data for all samples originally sequenced are shown in Additional files 3 and 4, respectively). To illustrate the sample selection procedures, Spearman correlation coefficients (according to RNA-seq ENCODE standard, 2016 [19]) and PCA analyses (Additional files 5 and 6, respectively) are provided. In all samples a minimum of two replicates were retained for the analysis. Pearson correlation coefficients and sample clustering heatmaps of the rlog-transformed data of selected samples are shown in Additional files 7 and 8, respectively.

### Preliminary analyses on nucleotide sequence differences between the two varieties compared to Vitis reference genome

As the full genomes of the two varieties of *V. vinifera* are not sequenced, some preliminary analyses were performed in order to estimate the accuracy of the comparison between the two grapevine cultivar transcriptomes. It should be stressed that, in case of strong sequence differences among two genotypes, such comparison may produce several artifacts raised by different mapping of reads on the reference genome which could therefore hamper read counts and called DEG fold changes. Towards the clarification of this issue, little data are available with respect to T. friulano and Chardonnay genomic diversity. Bacilieri et al. [20] reported a dendrogram based on 20 SSR markers in which Pinot noir and Chardonnay are grouped in the same clade, while Sauvignonasse (a synonym of T. friulano) is located in a different clade. This told, it is difficult to infer the extent of absolute differences in transcript sequences, as even the most distant grapevine varieties reported in the dendrogram could still exhibit sequence differences compatible with satisfactory read mapping percentages, at least for the adopted mismatches threshold (two mismatches for 51 nt read; i.e. a tolerance approaching 96% sequence identity).

Indeed, some insights with respect to the extent of genome diversity among the two genotypes can actually be obtained by our own data by comparing overall read mapping rates for all samples in both genotypes (Additional file 9). Based on this table, read alignment percentages summarize to 89.74% (SD 2.513) and 90.4% (SD 1.79), respectively, for T. friulano and Chardonnay. Therefore, within the limits of this similarity assessment based on global read mapping percentages, also with this approach the two genomes do not appear dramatically different in such a way to substantially hamper RNA-Seq approaches as the present one.

In addition to provide overall read mapping percentages, it was investigated whether, at least for a tractable subset of relevant genes consisting in the set of 8 genes used for the RNA-Seq validation, sequence differences could be detected and lead to severe alterations in read mapping patterns. Toward this end, after merging all alignment files (BAM files) from all samples for each of the two varieties, such merged alignments were compared on IGV (Integrative Genome Viewer) with allele frequency threshold set to 0.4. As shown in additional figure (Additional file 10), despite the fact that alignment files were not normalized for read abundance and that read coverage could differ legitimately due to diverse extents of expression level, read patterning appeared fairly similar for all genes but one, namely VIT_00s0233g00010 (Vacuolar invertase 2, GIN2). In the case of this gene, actually, the poor read coverage in T. friulano does not allow to compare potential mismatches among reads and gene sequences. Such altered patterning could also be compatible with poor expression of the invertase gene in T. friulano; however, the read patterning appears anomalous also in Chardonnay, suggesting that the reference gene model could be inadequate for both genotypes, making the read patterning poorly informative for both genotypes. However, among all the remaining cases, based on detected SNPs (shown as colored spikes in Additional file 10), the minimum identity was 98.4%, in the case of the greatest dissimilarity (with T. friulano sequence VIT_13s0064g01370 presenting 16 SNPs over approximately 1 Kb length versus reference genome). Finally, all the remaining genes of the validation set showed little or no SNPs, leading to an estimated sequence identity well over 99%, as expected in different varieties of the same species, and in some cases (e.g. VIT_18s0072g00260; Ethylene-responsive transcription factor, ERF) no SNPs could be detected in CDS.

Since all available data could not indicate dramatic differences at the sequence level among the two genotypes, it is reasonable to assume that a substantial proportion of DEG calls obtained when comparing the two transcriptomes proves to be reliable, even if some caution is mandatory, especially when evaluating the magnitude of called fold changes.

### Analysis of differentially expressed genes (DEGs)

Differences in gene expression between the two grapevine cultivars were analyzed comparing no insect treatments (Cha_noSt vs. To_noSt) and FD-free St or FD-infected St treatments (Cha_HSt vs. To_HSt and Cha_FDSt vs. To_FDSt, respectively) (Additional files 11 and 12). Moreover, for each grapevine cultivar and for each time point, two kinds of comparisons were set: namely, grapevine without St vs. grapevine with FD-free St and grapevine without St vs. grapevine with FD-infected St. These comparisons were designed to identify the responses of Chardonnay and T. friulano to insect piercing and sucking (HSt) and the responses to the three-trophic grapevine-phytoplasma-insect (FDSt) interaction, respectively (Additional files 13, 14, 15 and 16). The number of DEGs and enriched GO terms of the BP major classes called for each comparison at the FDR threshold of 0.05 are reported in Tables 1 and 2. The overlaps between up-regulated and down-regulated DEGs in the different treatments are illustrated with Venn diagrams reported in Additional file 17.

**Table 1:** Number of DEGs and enriched GO terms of the major biological process classes in the comparisons between cultivars

	Cha_HSt vs. To_HSt		Cha_FDSt vs. To_FDSt		Cha_noSt vs. To_noSt	
	3 dpi	6 dpi	3 dpi	6 dpi	3 dpi	6 dpi
n. DEGs	3739	1790	2581	2902	4982	3830
n. DEGs higher in To	1760	822	1207	1293	2480	1926
n. DEGs higher in Cha	1979	968	1374	1609	2502	1904
n. enriched GO terms (BP)	35	10	23	29	30	73

Cha Chardonnay, To Tocai friulano, dpi day post infestation

**Table 2:** Number of DEGs and enriched GO terms of the major biological process classes in the comparisons “noSt vs. HSt” and “noSt vs. FDSt” within each grapevine cultivar

	noSt vs. HSt				noSt vs. FDSt			
	Chardonnay		Tocai friulano		Chardonnay		Tocai friulano	
	3 dpi	6 dpi	3 dpi	6 dpi	3 dpi	6 dpi	3 dpi	6 dpi
n. DEGs	1656	526	815	949	460	1471	1735	1713
n. DEGs up-regulated	977	160	446	467	242	795	799	853
n. DEGs down-regulated	679	366	369	482	218	676	936	860
n. enriched GO terms (BP)	57	119	8	11	17	121	118	68

The annotation and analysis of DEGs were performed using GO, KEGG and MapMan orthologies. The MapMan desktop application coupled to Mercator has been used to visualize the DEGs identified in the present manuscript. Although this automatic procedure keeps the mapping pipeline efficiently updated with continual increasing of annotations and novel classifications of characterized proteins in any plant species, this approach resulted in many misleading annotations, because of the absence of the appropriate bins for many species. As a consequence, in general the analysis based on PageMan enrichment [21] needs to be considered as indicative and useful only to help interpretation of dataset rather than to give definite evidences. This is true above all for those biological processes that *V. vinifera* highly diversified in respect to other and model plant species. To face this problem, for all the pathways potentially important in our work, such as those related to the defense, an accurate analysis of each DEG was performed comparing manually the MapMan annotations in BLAST, and using also the GO annotations.

The expression profile of 15 genes was analyzed by qRT-PCR to validate and confirm the RNA-Seq data. Eight genes were selected based on their fold change values, some of them being chosen with low values and others with high ones, in order to validate the RNA-Seq technique. Moreover, seven genes specifically involved in the JA-related pathway were selected in order to confirm the main RNA-Seq results obtained in this work. For all these genes the fold change values obtained by qRT-PCR were very similar to those obtained by the RNA-Seq analyses (Additional file 18).

### Comparison of the constitutive transcriptome between the two grapevine cultivars

**Fig. 1:**
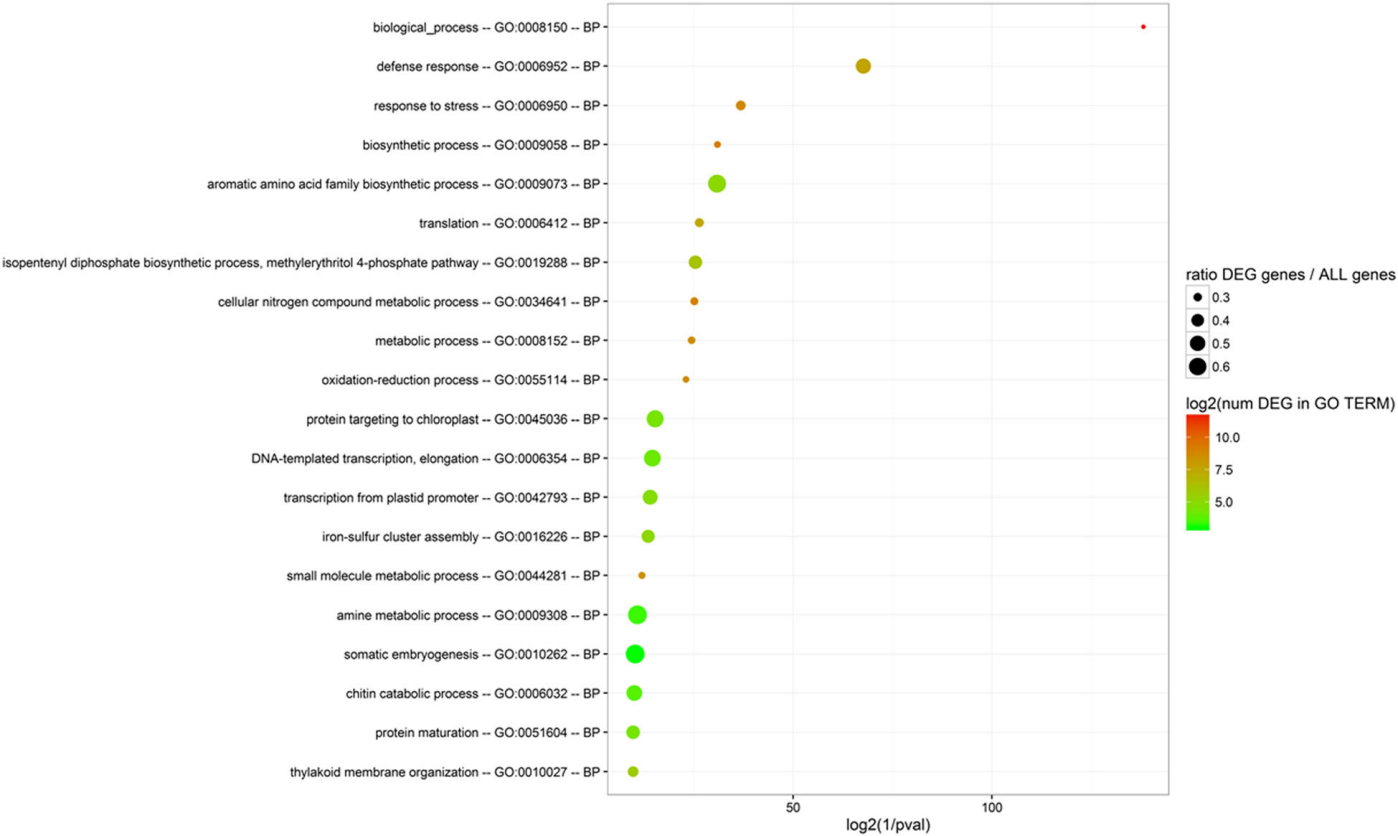
GO enrichment for Biological Process domain in the comparison of the constitutive transcriptomes of Chardonnay versus Tocai friulano. All noSt replicate treatments were considered. GO IDs and corresponding GO terms are as specified in the y-axis. In order to place most significantly enriched GOs at top on the y-axis, GOs are sorted according to decreasing log_2_(1/*p*-value) on the x-axis. The absolute number of DEGs that matched the GO term (log_2_-transformed) is indicated by the color of each spot, whereas the size of each spot shows the ratio of DEGs versus all grapevine genes matching the same considered GO term

The comparison of the transcriptomes of Chardonnay and T. friulano in the absence of *S. titanus* feeding was performed using all the noSt treatments. Taking into due account the cautionary notes summarized above, a total of 6233 DEGs were identified in the comparison: 3046 genes were more highly expressed in T. friulano, while 3187 genes showed a higher expression in Chardonnay (Additional file 19). Despite the high number of DEGs detected between the two transcriptomes, GO enrichment analysis showed only 20 over-represented GO terms in the BP domain, most of them involved in response to stress and secondary metabolic pathways (Fig. 1). These findings highlighted a substantial constitutive difference between the two transcriptomes with a specific focus on the genes associated with plant defense. The most frequently-present genes related to plant defense response were annotated as coding for putative proteins with the Toll-Interleukin receptor (TIR), Leucine-rich repeat (LRR) and nucleotide binding (NB) domains (*p* = 4.95E-13), which are all domains characteristic of molecules involved in surveillance systems triggered by the recognition of microbe-associated molecular patterns (MAMPs) or specific effectors released from pathogens [22]. Significantly higher expression levels of these genes were observed in the scarcely-susceptible cultivar: 145 DEGs more highly expressed in T. friulano belonged to this GO vs. 61 in Chardonnay. These critical regulatory components of plant constitutive resistance to invasive (hemi) biotrophic pathogens and TIR-NB-LRR protein-triggered resistance require some specific hydrolase proteins, such as those encoded by the *Senescence associated gene 101* (*SAG101*) [23], which was markedly more highly expressed in T. friulano.

Several genes encoding PR proteins showed constitutively higher expression levels in T. friulano compared with Chardonnay, such as four *PR-1,* one *PR-5 (thaumatin-like),* two *PR-6 (proteinase-inhibitor)* and two *PR-8 (chitinase class III)* genes. In the same cultivar, there was also a significant up-regulation of three genes encoding putative salicylic acid methyltransferase (SAMT), an enzyme that mediates the synthesis of gaseous methyl salicylate (MeSA), which was shown to be an inducer of the expression of the *PR-1* gene in tobacco [24].

**Fig. 2:**
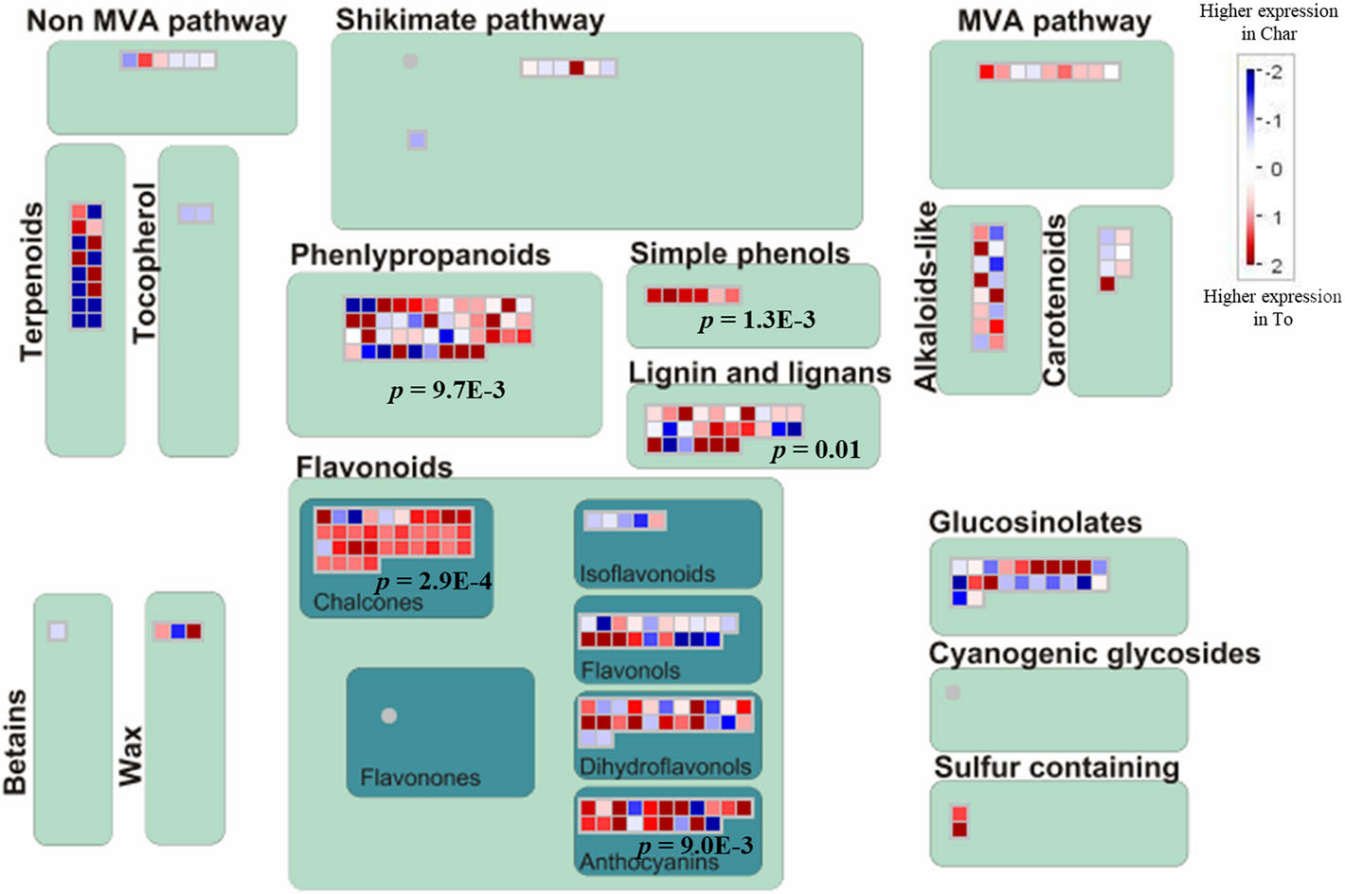
Constitutive differences in the expression of genes involved in the secondary metabolism between Chardonnay and Tocai friulano (comparison Cha vs. To_noSt), visualised by MapMan. Each entity within a pathway is depicted by a color signal where red signifies genes with higher expression in T. friulano compared to Chardonnay and blue signifies genes with higher expression in Chardonnay compared to T. friulano. The intensity of the color indicates the level of expression. Scale bar displays log2 fold changes. The *p*-values, obtained with Wilcoxon test, are reported for some bins

A constitutive different modulation in some branches of the secondary metabolism was observed between Chardonnay and T. friulano, above all in genes involved in phenolic metabolism, especially monolignol and stilbene biosynthesis (Fig. 2). Enhanced transcript levels of some genes involved in the general phenylpropanoid pathway were detected in T. friulano, namely the genes *phenylalanine ammonia lyase* (*PAL)* and 4*-coumarate-CoA ligase* (*4CL*). A branch of the phenylpropanoid pathway produces the precursors of monolignols, leading to the biosynthesis of lignans and lignin. Genes specifically dedicated to monolignols biosynthesis, such as those encoding cinnamoyl-CoA reductase (CCR), ferulate-5-hydroxylase (F5H), caffeoyl-CoA 3-O-methyltransferase (CCoAOMT) and cinnamyl alcohol dehydrogenase (CAD), showed a higher expression level in T. friulano. This part of the biosynthetic pathway is shared with the production of many volatile benzoids, such as eugenol, methyleugenol, chavicol and methylchavicol. Similarly, several genes coding for laccases, probably involved in the lignin polymerization process [25], showed a higher transcript number in T. friulano. The latter variety also exhibited a higher level of several genes coding for stilbene synthase (STS). Another branch of phenolic metabolism, involved in flavonoid biosynthesis, showed divergent profiles between the two grapevine varieties in the expression level of many genes encoding enzymes for the synthesis of flavones, flavonols and anthocyanins, such as *UDP-glycosyltransferase, flavonoid 3′-monooxigenase and isoflavone reductase*.

Several differences between T. friulano and Chardonnay were also observed in the constitutive expression of genes involved in terpenoid metabolism (Fig. 2). Two biosynthetic pathways are responsible for the synthesis of the universal precursors of all terpenoids. One of these, the 2-C-methylerythritol 4-phosphate (MEP) pathway, represented an enriched GO category, with as many as 64 genes more highly expressed in Chardonnay. In some plant species the MEP pathway is reported to be the main element responsible for the production of terpenes [26, 27]. The generation of an array of structurally-diverse terpenes, which takes place during the last step of terpenoid biosynthesis, is provided by the action of a large family of enzymes known as terpene synthases (TPSs) [28]. In this study, the two grapevine cultivars exhibited differing modulation in the profiles of the 16 genes annotated as putative *TPSs*, specifically involved in the production of monoterpenes, sesquiterpenes or triterpenes.

**Fig. 3:**
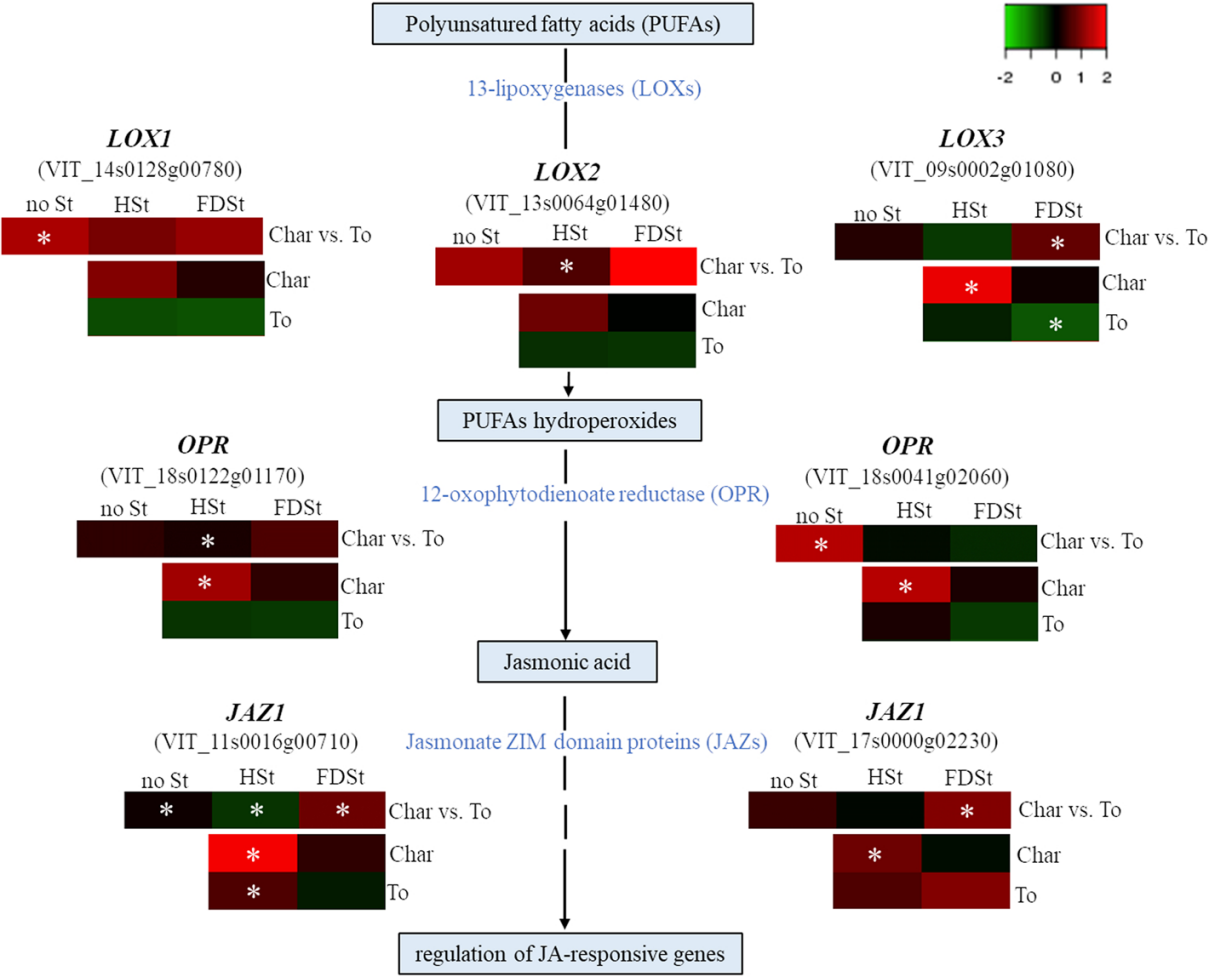
Transcriptional modulation of genes involved in the jasmonic acid pathway. Relative expression levels obtained by qRT-PCR analysis of three *13-lipoxygenases* (VIT_14s0128g00780, VIT_13s0064g01480, VIT_09s0002g01080), two *12-oxophytodienoate reductase* (VIT_18s0122g01170, VIT_18s0041g02060) and two genes encoding for jasmonate ZIM domain protein (VIT_11s0016g00710, VIT_17s0000g02230). The expression of each gene is represented by green-red heatmap. Differences in gene expression, evaluated on noSt, HSt and FDSt treatments (different columns), between Chardonnay and Tocai friulano are reported in the first row of each heatmap (Char vs. To), where red means genes with higher expression in T. friulano compared to Chardonnay and green means genes with higher expression in Chardonnay compared to T. friulano. The gene modulation induced by HSt and FDSt (different columns) in each grapevine cultivar is reported in the second and third rows (Char and To, respectively), where green and red correspond to low or higher transcriptional levels, respectively. Asterisks denote a significant gene modulation (*p*<0.05)

Finally, significant transcriptional differences between T. friulano and Chardonnay were observed for a group of genes involved in hormone biosynthesis, mobilization and signal transduction. For instance, many genes involved in the JA biosynthetic pathway showed a higher transcript level in T. friulano (*p* = 1.29E-7). They included two genes for oxo-phytodienoic acid reductase (OPR), an enzyme specific for JA biosynthesis, and eight genes encoding lipoxygenase (LOX), the key enzyme in the first general part of the octadecanoid pathway that leads to the production of jasmonates and of a group of oxylipins named green leaf volatiles (GLV). The highest modulated *LOXs* encoded for 13-LOX enzymes that catalyze the initial step of JA formation in plants [29]. The higher constitutive expression level of some genes involved in the JA-related pathway in T. friulano was confirmed by means of qRT-PCR (Fig. 3).

### Comparison of gene expression profiles between the two grapevine cultivars in response to HSt and FDSt feeding

**Fig. 4:**
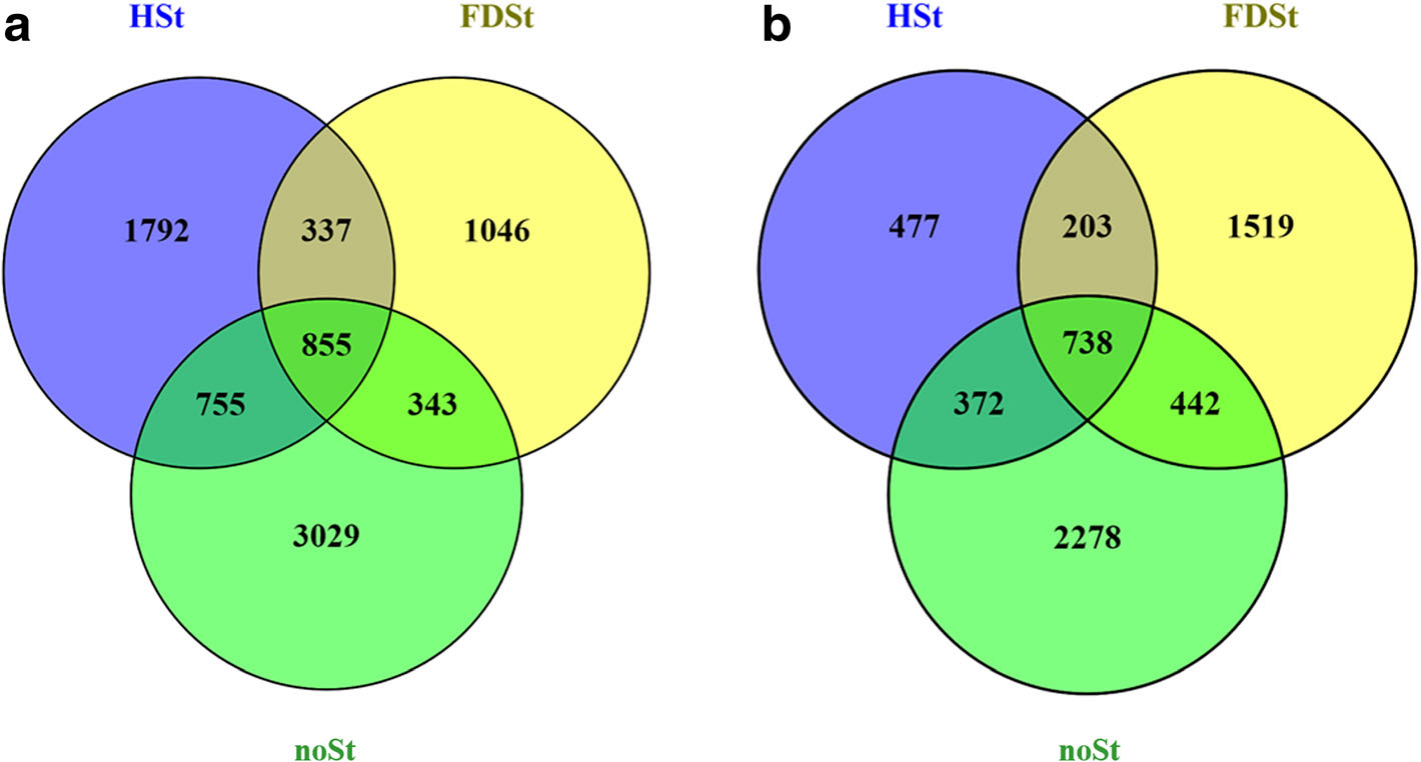
Venn diagram of genes differentially expressed between the two grapevine cultivars at constitutive level and after HSt and FDSt attacks. Venn diagram illustrating the relationships between DEGs identified in the three “Cha_HSt vs. To_HSt”, “Cha_FDSt vs. To_FDSt” and “Cha_noSt vs. To_noSt” comparisons at 3 (**a**) and 6 days post infestation (**b**)

**Fig. 5:**
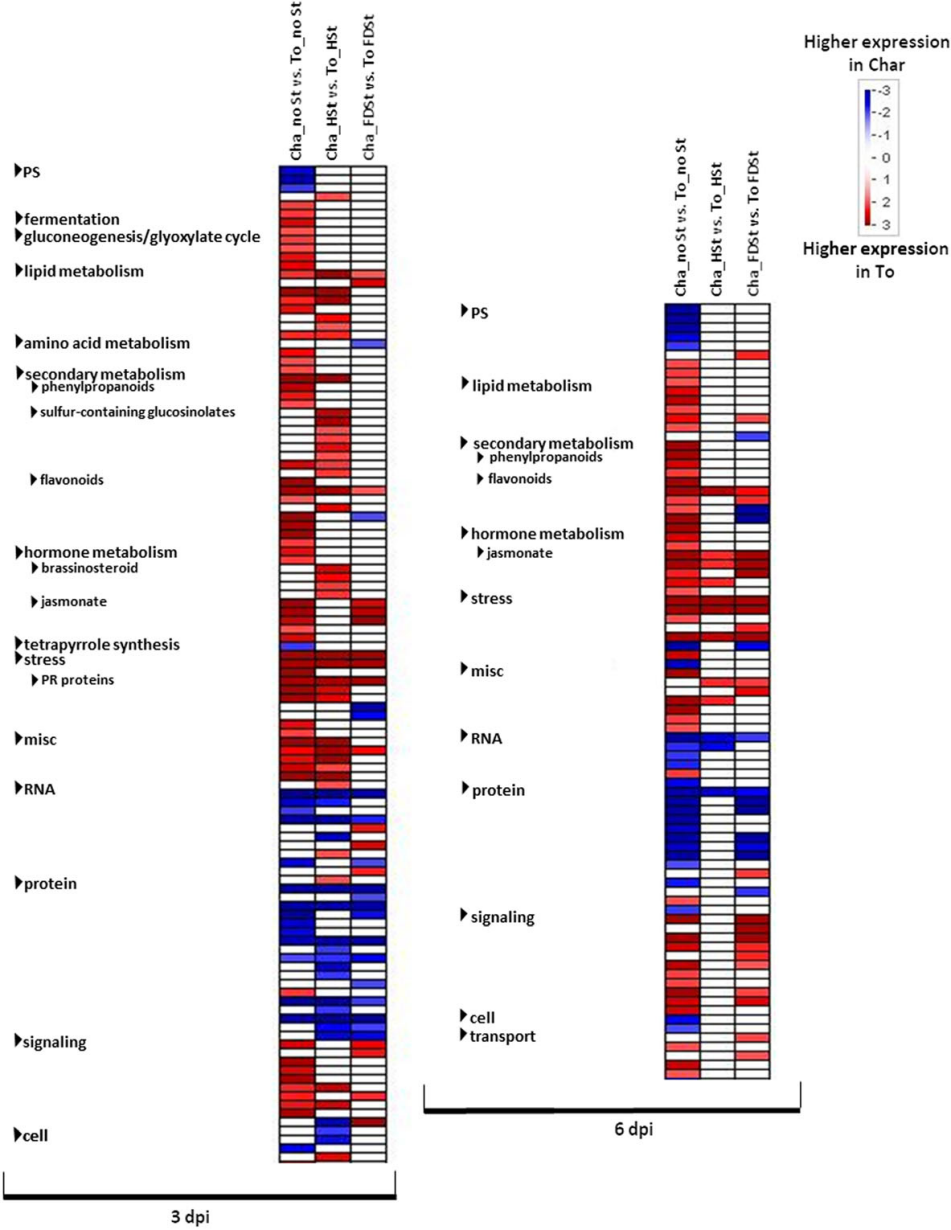
Overview of the significantly affected functional categories in the comparisons between Chardonnay and Tocai friulano. Changes in transcript levels are presented as log2 fold changes. The data were subjected to Wilcoxon test in PageMan, and the results are displayed in false-color code. Bins colored in red are significantly more expressed in T. friulano compared to Chardonnay, whereas bins colored in blue are significantly more expressed in Chardonnay compared to T. friulano. Differences in gene expression between the two varieties evaluated on noSt (Cha_noSt vs. To_noSt), HSt (Cha_HSt vs. To_HSt) and FDSt (Cha_FDSt vs. To_FDSt) treatments are reported on different columns. Changes in transcript levels registered at 3 and 6 dpi are shown

Transcriptional differences were evaluated between the two cultivars after HSt or FDSt attacks (deduced by the comparisons: Cha_HSt vs. To_HSt and Cha_FDSt vs. To_FDSt), together with the differences detected between the noSt samples (Fig. 4). In total, 855 and 738 DEGs were common among the comparisons between the two varieties in all experimental conditions at 3 dpi and 6 dpi, respectively. A statistically-based overview of the enriched functional categories (Fig. 5) indicated that treatments (noSt, HSt and FDSt) and time points (3 and 6 dpi) play a greater role than genotype in defining overall transcriptomic dynamics. Indeed, several bins were enriched for DEGs in noSt conditions but not in HSt or FDSt treatments.

Differences in the response induced by HSt feeding in the two grapevine cultivars were greater at 3 dpi than at 6 dpi. Indeed, 3739 and 1790 genes were differentially expressed in the two cultivars after HSt feeding at 3 and 6 dpi, respectively. Only 1164 genes modulated at 3 dpi remained DEGs also at 6 dpi, mainly maintaining the same expression profiles (Additional file 17). The Venn diagram illustrating the relationship between DEGs observed in the comparisons HSt, FDSt and noSt between the two cultivars revealed that a total of 1792 DEGs were specifically modulated in response to healthy insect attack at 3 dpi, while only 477 DEGs were associated to HSt feeding at 6 dpi (Fig. 4).

A different trend was observed in the comparison of the gene expression profiles for the two cultivars after FDSt infestation. Indeed, the number of DEGs was 2581 at 3 dpi and 2902 at 6 dpi, respectively, with 1046 and 1519 DEGs specifically induced by FDSt attack (Fig. 4, Additional file 17).

Despite known limitations in Mapman gene association to bins, such us with respect to isoprenoid pathways [30, 31], mainly overcame by manually annotations, Mapman ontology enrichment analysis of DEGs showed different set of enriched bins in the comparisons of HSt and FDSt responses between Chardonnay and T. friulano (Fig. 5). A large number of functional categories, involved in signaling and both in primary and secondary metabolism, mostly with constitutively-enhanced expression level in T. friulano, did not appear as enriched after HSt or FDSt feeding, indicating a substantial modulation of gene expression in at least one of the two grapevine cultivars. Most of genes involved in signaling, that showed higher transcription level in T. friulano in noSt condition and after FDSt attack at 6 dpi, were not enriched in HSt, both at 3 and 6 dpi, and in FDSt at 3 dpi. Indeed, differences in the transcription of the 282 genes coding for receptor-like kinases (RLKs), 167 of those over-expressed in Chardonnay, were observed after HSt feeding at 3 dpi. Seventy-one *RLKs* genes, mainly *Catharanthus roseus-like RLKs* and *thaumatin-like RLKs*, were identified as DEGs in Chardonnay and T. friulano only after HSt feeding. Furthermore, Ca^2+^ signaling became significantly enhanced after HSt attack, with 33 genes encoding Ca^2+^ binding proteins or Ca^2+^ transporting ATPase more highly expressed in Chardonnay (*p* = 6.92E-5). Among the hormone-dependent responses, several Mapman bins related to JA metabolism were enriched in DEGs in noSt and FDSt comparisons between the two cultivars but not in HSt. The enrichment of some bins of the brassinosteroid metabolism, with higher mRNA amount in T. friulano, was specific of HSt response. Several classes of the secondary metabolism were differentially modulated between the two cultivars in most of the conditions. Most of the bins related to phenylpropanoid and flavonoid metabolisms displayed a constitutive higher expression in T. friulano, but did not show any enrichment after HSt and FDSt attacks (Fig. 5). However, there was a significant enrichment of the sulfur glucosinolate metabolism observed specifically after HSt feeding at 3 dpi, with a higher expression level in T. friulano. At 6 dpi after FDSt treatment there was a significant difference between Chardonnay and T. friulano concerning the expression level of genes involved in the synthesis of phenylpropanoids and stilbenes. Indeed, an enhanced expression of six genes of the general phenylpropanoid pathways (*PAL, C4H, 4CL*) was observed in Chardonnay, besides a significant over-expression of 29 genes encoding STS.

Concerning primary metabolism, interestingly only a few bins related to photosynthesis or involved in lipid and cell wall metabolisms were enriched in the HSt and FDSt comparisons at 3 and 6 dpi, while at constitutive level several bins related to photosynthesis showed a lower expression level in T. friulano in comparison to Chardonnay, and many bins involved in lipid and cell wall metabolisms revealed higher transcription level in T. friulano.

### Differential gene expression profiles induced by HSt and FDSt in each grapevine cultivar

**Fig. 6:**
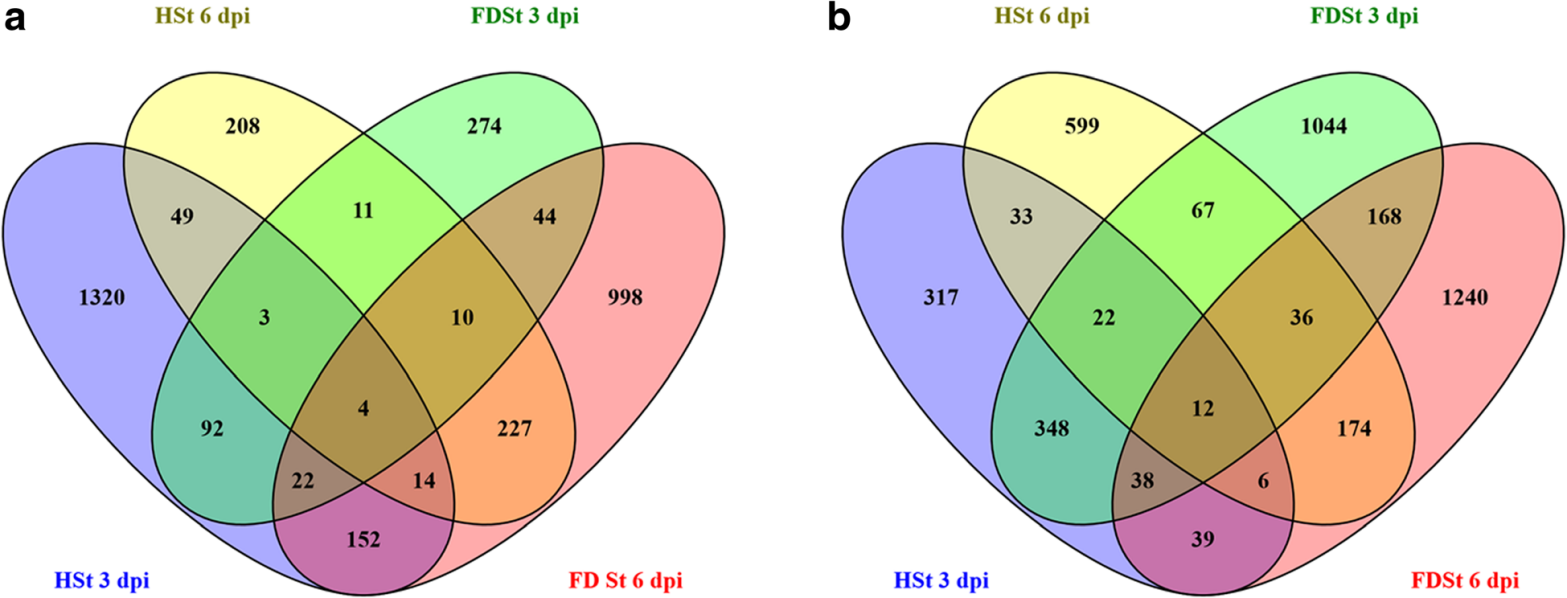
Venn diagram of genes modulated after HSt and FDSt attacks in the two grapevine cultivars. Venn diagrams illustrating the relationships between the DEGs induced by HSt and FDSt at 3 and 6 days post infestation on Chardonnay (**a**) and Tocai friulano (**b**)

In Chardonnay, the number of DEGs in response to HSt was higher at 3 dpi than at 6 dpi (1656 and 526, respectively, Table 2), with only 70 genes shared between the two time points. GO enrichment analysis yielded 58 and 120 enriched GO terms of the BP class, at 3 and 6 dpi, respectively, with 27 common GO terms. In the same cultivar, an increased number of DEGs was detected in response to FDSt infection throughout the trial (460 and 1471 at 3 and 6 dpi, respectively) with 80 common genes, as well as an increased number of enriched GO terms (18 and 122 in the BP class, respectively, nine of those shared between the two time points) (Fig. 6a).

On the other hand, a similar number of genes were differentially modulated in T. friulano at 3 and 6 days after HSt feeding (815 and 949 DEGs, respectively). GO enrichment analysis revealed that, within the BP class, only 2 GO terms were common to the different time points. The number of DEGs in response to FDSt infection seemed rather similar at both time points, being 1735 and 1713 at 3 and 6 dpi, respectively. However, only 254 DEGs and 39 enriched GO terms of BP were shared between the two time points (Fig. 6b).

**Fig. 7:**
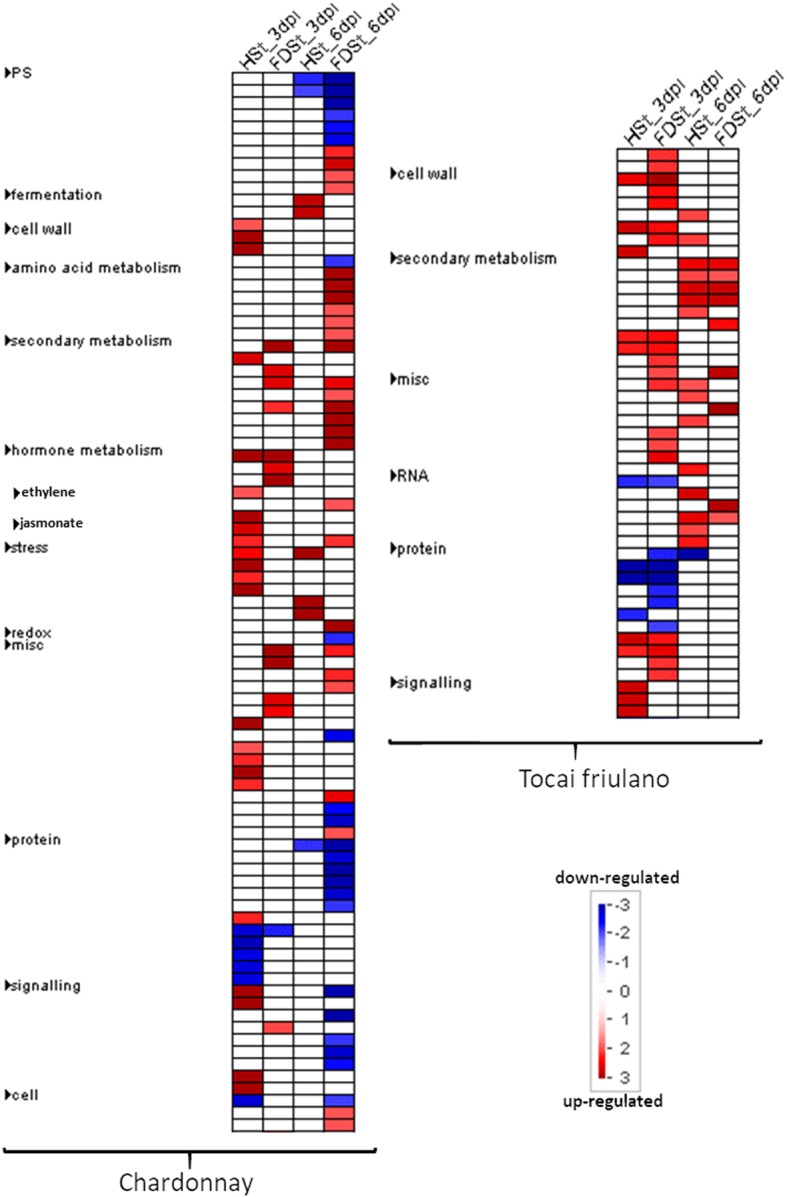
Overview of the significantly affected functional categories in Chardonnay and Tocai friulano in HSt and FDSt treatments at 3 and 6 dpi. Changes in transcript levels are presented as log2 fold changes. The data were subjected to Wilcoxon test in PageMan, and the results are displayed in false-color code. Bins colored in red are significantly up-regulated, whereas bins colored in blue are significantly down-regulated

**Fig. 8:**
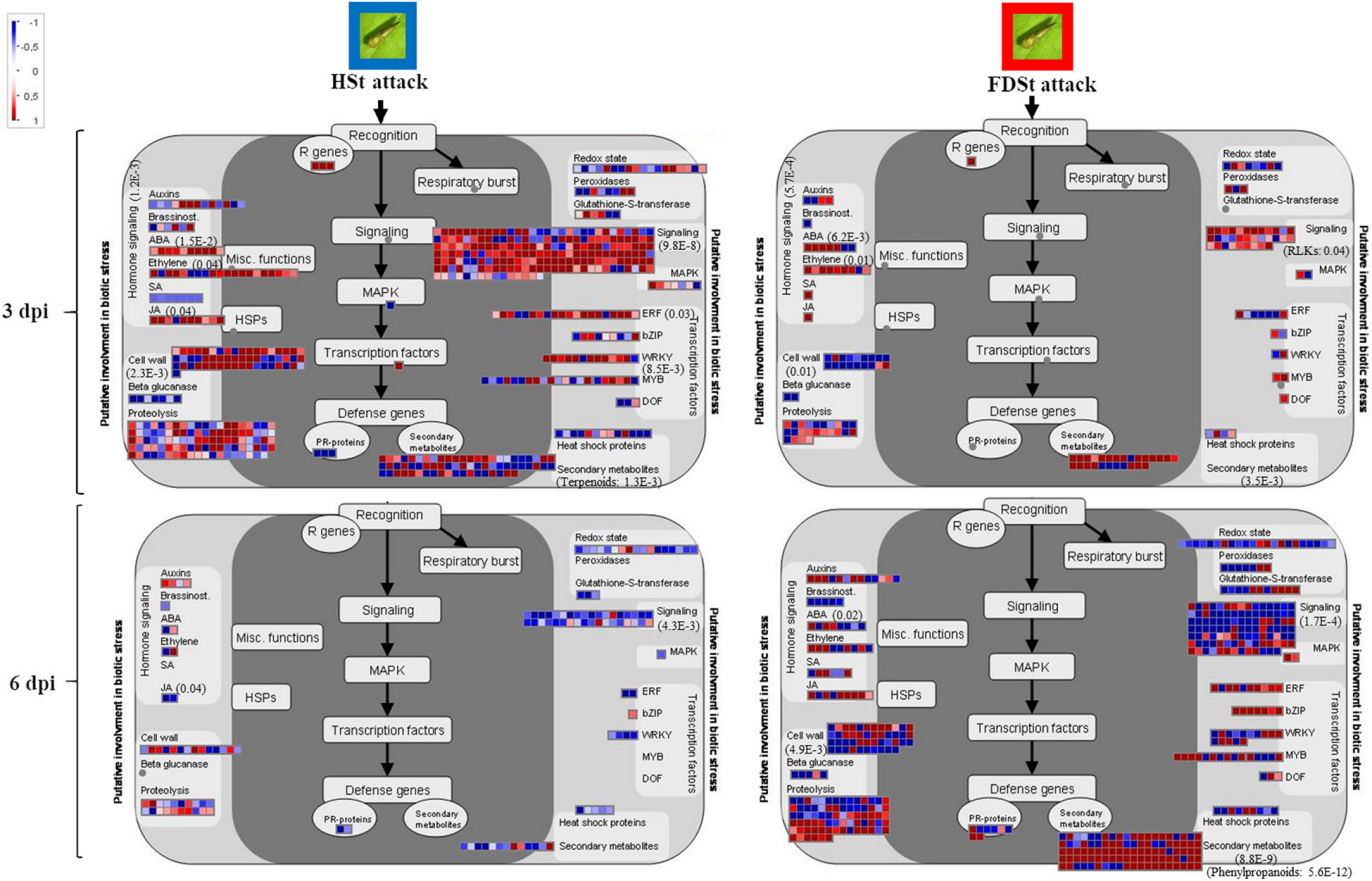
MapMan illustration depicting DEGs from the “Biotic stress” bins affected by HSt and FDSt attacks in Chardonnay. Transcriptomic data obtained from HSt and FDSt treatments, at 3 and 6 dpi, were compared to their respective controls (noSt). Log2 fold changes are indicated as a gradient of blu (down-regulated) and red (up-regulated). The *p*-values are indicated for some bins. Details of Mapman Wilcoxon’s rank sum test reporting the *p*-values for the different bins are reported in Additional file 18

**Fig. 9:**
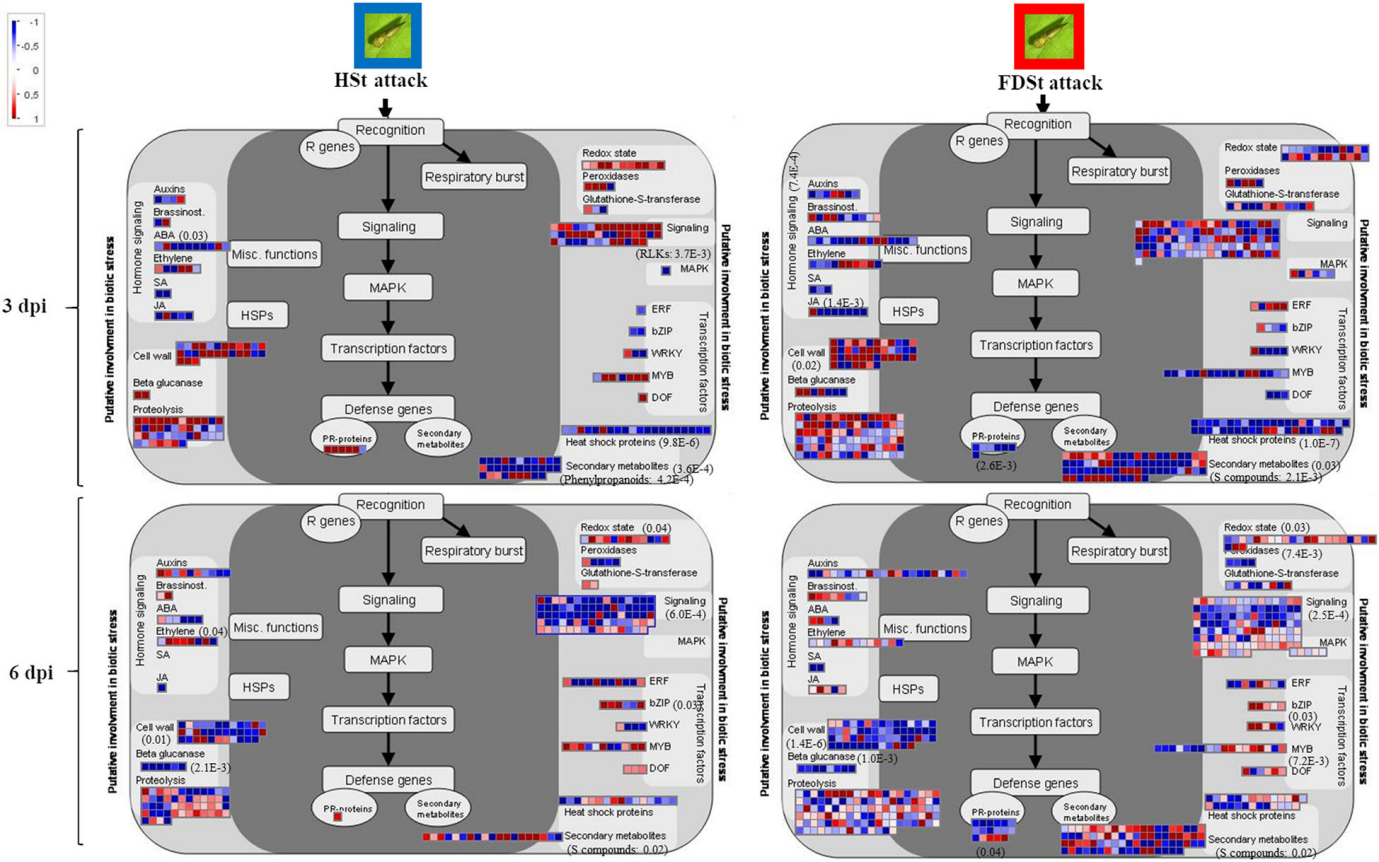
MapMan illustration depicting DEGs from the “Biotic stress” bins affected by HSt and FDSt attacks in Tocai friulano. Transcriptomic data obtained from HSt and FDSt treatments, at 3 and 6 dpi, were compared to their respective controls (noSt). Log2 fold changes are indicated as a gradient of blu (down-regulated) and red (up-regulated). The *p*-values are indicated for some bins. Details of Mapman Wilcoxon’s rank sum test reporting the *p*-values for the different bins are reported in Additional file 19

Mapman ontology enrichment analysis of DEGs showed different set of enriched bins modulated by HSt and FDSt attacks at 3 and 6 dpi in each variety (Fig. 7). Several affected metabolic pathways were involved in the plant reaction to biotic stress (Figs. 8 and 9), sometimes in a significant statistical manner (Additional files 20 and 21). The categories of genes modulated after HSt or FDSt feeding in each variety are described in detail in the following chapters and the heatmaps are reported in Additional files 22 and 23.

### Signaling pathways

In plants, several specialized signaling pathways, largely governed by protein kinases and Ca^2+^, are involved in the perception and transduction of environmental stimuli [32]. These pathways were significantly modulated in both varieties in most of the conditions (Figs. 8 and 9, Additional files 20 and 21).

In detail, the expression of a large number of genes coding for different groups of protein kinases, mainly RLKs, was modulated in response to HSt and FDSt (Additional files 22 and 23). In the early response of Chardonnay to HSt infestation (3 dpi) 81 genes encoding RLKs were identified as DEGs. Most of them were up-regulated and included some *RLK* family members (i.e. *RLK10*-*like*), *thaumatin-like* and *cell wall-associated-like kinases* (*WAKs*), which had been reported in plant-microbe interactions and stress response [33]. The *protein kinase* genes were also present among DEGs in T. friulano, but in a smaller amount. Indeed, in the latter variety insect piercing and sucking caused an increased abundance of transcripts for only 12 genes coding RLKs, above all the thaumatin-type ones. On the other hand, the response of both cultivars six days after FDSt infection was characterized by a general down-regulation of the expression of *RLKs* belonging to different families (28 and 32 genes in Chardonnay and T. friulano, respectively), including nine genes encoding thaumatin-like RLKs in Chardonnay, and 15 genes coding for receptors of the leucine-rich repeats family in T. friulano.

Many calcium-mediated signaling associated genes were significantly activated in the early response to HSt feeding (3 dpi) only in Chardonnay (Additional file 20), while the expression of very few genes was modulated in T. friulano (Additional files 22 and 23). In Chardonnay the up-regulation of 40 genes (out of the 44 DEGs involved in the calcium-mediated signaling pathway) was observed, many of them encoding Ca^2+^ − binding proteins, such as calmodulin and calmodulin-like proteins (CMLs), responsible for the sensing of the elevated level of Ca^2+^, and Ca^2+^-dependent protein kinases, which phosphorylate many regulatory proteins. Interestingly, HSt feeding in Chardonnay caused a significant accumulation of *CML37* and *CML38* transcripts, which had been reported to rapidly increase adjacent to a wound site [34]. On the other hand, in the two cultivars, 6 days after FDSt infection, a decreased abundance of transcripts for genes involved in calcium signaling was observed (15 out of 24 in Chardonnay and 15 out of 21 in T. friulano) (Additional file 15).

### Hormone-related pathways

Defense responses in plants are mediated by an interconnected network of signal transduction pathways depending mainly on hormones, such as salicylic acid (SA), JA, ethylene (ET) and abscisic acid (ABA) [35]. A significant modulation of JA-, ET- and ABA-pathways was observed only at 3 dpi after HSt treatment in Chardonnay (Fig. 7).

Indeed, in Chardonnay, after HSt feeding at 3 dpi, many DEGs involved in the JA, ET and ABA synthesis and signal transduction pathways were detected, most of them up-regulated (Fig. 8, Additional files 22 and 24). The expression of several genes of the octadecanoid pathway, involved in the JA biosynthesis, were strongly up-regulated, such as three *LOX*, two *OPR* and one gene encoding JA methiltransferase (*JAMT*). Moreover, the induction of some *JAZ/TIFY* genes was observed. These are known to encode proteins that repress the activity of transcription factors (i.e. MYC2), which positively regulate JA-responsive genes. In *Arabidopsis* in response to environmental or developmental signals that stimulate the biosynthesis of JA, the activation of *MYC2*, as a result of the degradation of JAZ/TIFY proteins, induces the transcription of early JA-responsive genes, including *JAZ/TIFY* genes themselves [36]. Activation of the ET-signaling pathway in response to HSt infestation might be hypothesized by the up-regulation of genes coding for two enzymes of the ET biosynthesis (*1-aminocyclopropane-1-carboxylic acid (ACC) synthase (ACS) and ACC oxidase (ACO)*) and the up-regulation of many genes putatively encoding transcription factors of the APETALA2/ethylene-responsive factor (*AP2/ERF*) family, as detailed in the next dedicated section. Moreover, the feeding of HSt in Chardonnay significantly induced several genes involved in the ABA-controlled signaling and biosynthesis, such as *9-cis-epoxycarotenoid dioxygenase* (*NCED*) and *ABA-aldehyde oxidase* (*AAO*). A lower number of DEGs involved in the JA-, ET- and ABA-signaling cascades was observed to be modulated in Chardonnay after FDSt infection (Fig. 8, Additional files 22 and 24). For example, among genes involved in the JA-defense response, 10 DEGs were identified in response to HSt infestation and only one *LOX* gene after infection of FDSt. Moreover, among genes known to be involved in the ET-signaling pathway, 20 and nine DEGs were detected after feeding of HSt and FDSt, respectively. Lastly, the expression of eight genes related to ABA-controlled signaling was up-regulated after HSt infestation and that of five out of seven genes was induced after FDSt infection.

In T. friulano the feeding of HSt and FDSt modified the expression of the genes involved in hormone signaling pathways to a lesser extent than in Chardonnay, and the differences were not statistically significant (Fig. 9, Additional files 23 and 24). Eight out of ten genes involved in the signaling and metabolism of ABA were down-regulated. Similarly, the transcription of genes encoding for some ABA responsive proteins was repressed after FDSt infection (11 out of the 14 DEGs involved in the ABA-related signaling), in addition to some genes of the octadecanoid pathway (two *LOX*, three *OPR*, one *JAMT*).

Different modulation of some genes involved in the JA-related pathway between Chardonnay and T. friulano after HSt and FDSt attacks was confirmed by means of qRT-PCR (Fig. 3).

### Transcription factors

In Chardonnay, many genes encoding transcription factors, including those belonging to the *AP2/ERF, WRKY, NAC, MYB* and *DOF* families, were differentially expressed during the time-frame of the experiment (Additional file 22). A large number of DEGs encoding transcription factors were observed after 3 days in response to HSt infestation, while only a few DEGs were detected after FDSt infection in the same cultivar. Indeed, several genes encoding AP2/ERF members were strongly up-regulated after HSt feeding (16 out of 20), while only two out of seven genes were up-regulated in response to FDSt infection (Additional file 20). Moreover, some *WRKY* genes showed increased expression specifically in HSt samples, such as three genes encoding for WRKY53, which had been functionally characterized as a positive regulator of defense response [37].

On the other hand, a lower number of DEGs encoding *WRKY* or *AP2/ERF* members were observed after infestation by HSt or FDSt in T. friulano (Additional file 23), while a significant modulation of genes encoding *MYB* transcription factors occurred after FDSt infection, both at 3 and 6 dpi (Fig. 9, Additional files 21 and 23).

### Antioxidant mechanisms

In plants, several enzymatic and non-enzymatic antioxidant mechanisms could be activated as protection against invading pathogens in addition to signaling systems [38]. In this experiment, the expression of many genes involved in the control of the cellular redox state was modulated in response to HSt or FDSt feeding in both cultivars (Figs. 8 and 9). However, a clear trend was observed only in the T. friulano response to HSt feeding at 3 dpi, with 15 up-regulated genes out of 17 DEGs (Additional file 23). Among these, some genes which performed a detoxifying function on reactive oxygen species (ROS), such as those coding for superoxide dismutase (SOD) and peroxidase (POD), together with non-enzymatic antioxidants, such as ascorbate peroxidase (APX) and glutathione S-transferase (GST), were present. Activation of these pathways had been observed in coconut and grapevine varieties which are scarcely-susceptible to phytoplasmas, both with transcriptomic and proteomic analyses [15, 39].

### Cell wall metabolism

The transcription of several genes involved in cell wall metabolism was affected after HSt or FDSt attacks in both varieties. An enrichment of some bins related to cell wall metabolism was detected at 3 dpi after HSt feeding in both varieties, and, in a most consistent way, after FDSt treatments in T. friulano (Fig. 7).

In a detailed analysis of the single DEGs it was observed that transcripts of enzymes involved in cell wall precursor biosynthesis - leading to the production of sucrose, galactose, UDP-D- glucuronic acid and subsequently of UDP-D-xylose and UDP-D-galacturonic acid - were induced in Chardonnay three days after HSt feeding and six days after FDSt infection. Moreover, ten transcripts coding for cellulose synthase and one for hemicelluloses were induced after HSt infestation. Interestingly, the genes involved in the hydrolysis of polysaccharides, like *xyloglucan endotransglycosylase* (*XTH*) and *expansin*, were also induced during the response of Chardonnay to HSt at 3 dpi, while the genes coding for pectinesterases were repressed (Fig. 8, Additional files 22 and 24).

Modulation in the expression of several genes involved in cell wall modification was also observed in T. friulano after HSt feeding, but with an opposite trend at 3 and 6 dpi (Fig. 9, Additional files 23 and 24). Indeed, several genes coding for pectin lyase, pectin hydrolase- and pectin methylesterase- like proteins, involved in cell wall degradation, were up-regulated at 3 dpi (15 out of 22 DEGs). Conversely, several genes related to cell wall metabolism were down-regulated at 6 dpi (25 out of 32 DEGs). A reduced expression of *expansin* and *cellulose synthase* genes, as well as of *pectin lyase* and *polygalacturonase* genes was observed, together with an increased expression of *XTH* genes. An analogous response was observed after FDSt infection. Indeed, 14 out of the 22 genes involved in the synthesis of cell wall elements were up-regulated at 3 dpi, as well as another 13 out of the 18 DEGs which were involved in the degradation of the cell wall. On the other hand, a large number of genes involved in cell wall metabolism were down-regulated at 6 dpi. They included nine genes encoding enzymes for the biosynthesis of cell wall-elements, in particular cellulose synthase, and another 28 genes involved in the degradation/modification of the cell wall, such as *XTH* and *pectin lyase* genes.

### Defense proteins

A few DEGs encoding various defense-related proteins, which can possess antimicrobial and anti-insect activities, were found in Chardonnay after HSt and FDSt feeding (Fig. 8, Additional file 22).

In T. friulano, five genes coding for pathogenesis-related proteins PR-1 were significantly up-regulated after HSt feeding at 3 dpi (Fig. 9, Additional file 23). Moreover, a number of *protease* genes were induced, in particular some *subtilisin-like protein* genes, which had been reported to be involved in both immune responses and programmed cell death [40]. Interestingly, in the same cultivar, three genes encoding PR-6, with proteinase-inhibitor properties, were down-regulated after FDSt infection.

### Secondary metabolism

Several bins involved in the secondary metabolism were enriched at 6 dpi after FDSt feeding in Chardonnay and after HSt and FDSt attacks in T. friulano (Fig. 7).

In a detailed analysis of the single DEGs it was observed that the expression of several genes involved in the phenolic metabolism was up-regulated in Chardonnay in response to FDSt at 6 dpi (Additional files 22 and 24). Among these, *PAL* and *cinnamate 4-hydroxylase* (*C4H*) genes were observed. They belong to the phenylpropanoid pathway, which starts with the deamination of phenylalanine and leads to hydroxycinnamoyl-CoA esters. Moreover, the over-expression of 22 genes encoding STS was observed. This enzyme catalyzes the biosynthesis of the stilbene backbone from the intermediates of the general phenylpropanoid pathway, leading to the production of metabolites which play key roles in plant protection [41]. Among genes with significantly up-regulated expression levels, 11 genes coding for putative laccases were also identified. The response of T. friulano seems different, as the expression of five genes encoding STS and of two genes coding for the laccase were down-regulated in response to FDSt at 6 dpi, while three days after HSt feeding the transcription of some *flavonoid reductases* and *oxoglutarate oxygenases,* belonging to the flavonol metabolism, was up-regulated (Additional files 23 and 24).

In Chardonnay, HSt feeding elicited, at 3 dpi, the accumulation of *3 (+)-delta-cadinene synthase* transcripts. This enzyme catalyzes a reaction in the synthesis of the terpenoid delta-cadinene, an important constituent of essential oils. The response of T. friulano to FDSt infection was characterized at both time points by the up-regulation of several genes involved in the synthesis of terpenoids at different levels, and in particular of carotenoids at 6 dpi (Additional files 23 and 24).

### Primary metabolism

A significant enrichment of several bins related to photosynthesis was observed in Chardonnay six days after HSt and mainly after FDSt attacks (Fig. 7).

In a detailed analysis of the single DEGs it was observed that several DEGs involved in photosynthesis-related processes of both light and dark phases were identified in Chardonnay and all of them showed a general down-regulation in response to HSt or FDSt feeding (Additional file 22). Transcripts coding for RubisCO were down-regulated, as were transcripts coding for chlorophyll-binding proteins, ATP synthase, cytochrome b6 and electron chain ferredoxins. Notably, almost all DEGs related to photosynthesis and identified in this study are well known to be repressed in symptomatic grapevines infected with phytoplasmas [13, 14].

In T. friulano the modulation of a number of genes involved in photosynthesis and sugar metabolism was observed after HSt or FDSt infestation (Additional file 23). Indeed, two *threalose synthase* genes were induced after HSt feeding at 3 dpi and repressed at 6 dpi, while the expression of *raffinose synthase* was down-regulated at 3 dpi and 6 dpi. The involvement of threalose in aphid defense had been reported for peach [42]. Sucrose and starch metabolism are also generally modified during insect attacks, since feeding causes the loss of carbon resources, mainly in the form of sucrose, leading to changes in the source-sink nature of the infested tissue [43]. Indeed, the expression of some genes involved in starch and sucrose degradation was down-regulated at 6 dpi. FDSt infection stimulated the expression of many genes encoding components of the photosystems in the same cultivar, with a greater number of genes modulated three days after FDSt infection, which also induced genes belonging to the Calvin cycle and to chlorophyll and carotenoid synthesis. Moreover, some genes responsible for synthesis of raffinose family sugars were down-regulated after FDSt feeding (*galactinol synthase* and *raffinose synthase*), while the genes for threalose synthesis (*threalose synthase* and *phosphatase*) increased at 3 dpi, as after HSt infection. The expression of a few genes belonging to the starch degradation pathway was induced at 3 dpi, although some genes of the following glycolysis pathway and Krebs cycle were slightly down-regulated at both timings.

### Co-expression analyses

Modulation of gene expression induced by HSt and FDSt feeding in Chardonnay and T. friulano was further investigated by means of co-expression analysis. DEGs that manifested similar expression pattern in different conditions (noSt, HSt and FDSt) were grouped into 15 major clusters, the largest containing 145 genes and the smallest composed of ten genes. The complete list of genes belonging to each cluster, their annotation and the results of the GO enrichment analysis are reported in Additional files 25 and 26. In general, co-expression results confirmed, and reinforced data obtained by the previous analyses.

The presence of two major clusters, 1 and 2, that included genes showing a higher expression level in all the experimental T. friulano or Chardonnay conditions, respectively, confirmed the wide difference in the constitutive transcriptome observed between the two varieties. Cluster 1 (T. friulano) consisted of 145 genes, most of them belonging to the GO terms “Defense response” (*p* – value = 4.71E-9) and “Response to stress” (*p* – value = 2.32E-4), such as 21 genes encoding NB-LRR resistance proteins and two genes coding for RLKs. Moreover, four genes encoding antioxidant enzymes, such as GST, catalase and peroxiredoxin (PRX), were included in cluster 1. Conversely, despite the fact that cluster 2 (Chardonnay) was composed of 100 genes, only one enriched GO term was identified (“ATP biosynthetic process”). Among defense-related genes, six *NB-LRR* resistance genes, four genes encoding RLKs and two genes coding for antioxidant enzymes (one *SOD* and one *PRX*) were present in cluster 2. This result reiterates that in T. friulano many genes involved in plant defense were constitutively expressed at higher level than in Chardonnay.

Several genes were specifically modulated after HSt feeding and were included in four clusters: two clusters with genes specifically-modulated in Chardonnay (5 and 12), one cluster with genes up-regulated in T. friulano (13) and one cluster with genes up-regulated in both cultivars (6). Most of the genes with higher expression level in Chardonnay at 3 dpi (clusters 5 and 12) were involved in cell wall metabolism and in different signaling pathways, confirming the above reported results. In details, cluster 5 contained 29 genes more highly expressed in Chardonnay HSt at 3 dpi. In this cluster the most represented GO term was “Glucan metabolic process”, with eight genes coding for XTH, enzyme affecting cell wall metabolism. Moreover, 11 genes involved in signaling were included, coding for two NB-LRR resistance proteins, three Ca^2+^ − binding proteins CML31 and five transcription factors of the AP2/ERF family tightly co-regulated with a TIFY transcription factor. A further 13 genes, up-regulated three days after HSt feeding in Chardonnay, were grouped in cluster 12. In this cluster, three genes encoding NB-LRR resistance proteins and six genes coding for RLKs, such as WAK-like kinases, were present. Eleven genes belonging to cluster 13, despite high standard deviation among biological replicates, were characteristic of T. friulano response at 3 days after HSt feeding. Among the annotated genes, three genes encoding proteins involved in defense response were included: a GDSL lipase, a strictosidine synthase, which is a key enzyme in alkaloid biosynthesis, and a PR1 protein. Six days after HSt feeding several genes involved in the cell wall metabolism were modulated in both varieties, and were grouped in cluster 6, consisting of 28 genes. It showed the highest enrichment of the GO terms “Cell wall biogenesis” and “Glucuronoxylan metabolic process”, with ten and eight members, respectively, encompassing genes coding for cellulose synthase, chitinase and xylosidase. Moreover, four genes belonging to the “Lignin metabolic process” were identified. Among functionally annotated genes including in cluster 6, it is interesting to note the presence of a *NAC* transcription factor, belonging to a class of gene regulators shown to be the activators of the common transcriptional network for the biosynthesis of lignin, cellulose and xylan [44].

Six clusters (4, 7, 10, 11, 14, 15) included genes specifically modulated after FDSt infection, indicating different transcriptomic profiles induced in the two grapevine cultivars at different time points. Clusters 10 and 15, specific of the Chardonnay response at 3 dpi confirmed the up-regulation of genes involved in the cell wall reinforcement and the down-regulation of genes involved in photosynthesis, respectively. At 3 dpi, 15 genes, belonging to cluster 10, were induced in Chardonnay. They mainly encoded proteins involved in the biosynthesis of cutin, suberin and wax, such as omega-hydroxypalmitate O-feruloyl transferase (HHT), cytochrome P450 86A1 (CYP86A1), fatty acyl-CoA reductase and GDSL lipase. Genes encoding peroxidase 11 and laccase 14, enzymes involved in the biosynthesis and degradation of lignin, belonged to cluster 10 as well. In the same cluster, a gene coding for a CASP-like protein, with an essential role in directing a local cell wall modification was included, together with a NAC transcription factor [45]. Cluster 15 encompassed ten genes down-regulated in Chardonnay at 3 days after FDSt infection, which were mainly involved in photosynthesis. Further genes which had been up-regulated at 6 days after FDSt feeding were divided into four clusters: two characteristics of Chardonnay (4 and 7), one specific to T. friulano (11) and another with genes modulated in both cultivars (14). Among the largest clusters, cluster 4 assembled 30 genes with a higher expression level in Chardonnay after FDSt feeding, confirming an enhanced modulation of the phenylpropanoid pathway. Indeed, several genes belonging to cluster 4 were involved in the metabolism of phenylpropanoids and lignin: three *laccases* and five *STS* were tightly co-regulated with the transcription factor *MYB14*, which had been reported to directly activate transcriptional expression of the *STS* gene [46]. As regards genes specifically induced in T. friulano, cluster 11 included 14 genes, many of which were involved in defense response. The presence of genes coding for LOX and galactolipase DONGLE, an enzyme involved in the hydrolysis of long fatty acid chains, suggests that the JA synthesis was activated. Furthermore, in cluster 11 some genes were present which were involved in cell wall metabolism, such as those coding for pectin esterase and laccase. Finally, cluster 14 included 11 genes which had been up-regulated after FDSt in both cultivars. It is interesting to note the presence in this cluster of four genes encoding reticuline oxidase-like protein, an enzyme that catalyzes H_2_O_2_ production by using hexose sugars, thus mediating constitutive resistance against pathogens [47].

## Discussion

In this study, RNA-Seq was used to compare early transcriptional changes occurring during the three-trophic interaction between the FD phytoplasma, its vector *S. titanus* and the grapevine, represented by two different cultivars: the highly-susceptible Chardonnay and the scarcely-susceptible T. friulano.

The experimental plan made use of micropropagated plantlets, reared vectors and field sources of inoculum, aiming to mimic the natural event of infection in controlled conditions. However, this strategy is not devoid of challenges, such as the impossibility of rearing the insect for its whole life cycle, the low rate of transmission in the experimental conditions used, and the impossibility of inoculating a controlled amount of the pathogen. In particular, the use of insect vectors could introduce possible undeterminable effects, related to biological differences among single insects, such as sex, feeding pressure, possible oviposition. On the other hand, direct inoculation of the grapevine with phytoplasmas is still not possible, as the pathogen cannot be transmitted from axenic culture to plant.

A similar experimental plan had recently been used for evaluating the susceptibility to FD of several grapevine varieties and species in controlled conditions [12]. The results confirmed that both the percentage of experimentally-infected plants and the FD phytoplasma titre are linked to susceptibility. The same conclusion was reached in field experiments with highly-susceptible Barbera and moderately-resistant Nebbiolo cultivars [17]. In the present paper, no statistically-significant difference in the percentage of infected plantlets or in phytoplasma concentration was clearly detected between T. friulano and Chardonnay. However, the current evaluation was carried out at a very early stage (3 and 6 dpi), while Eveillard et al. [12] identified diversities in pathogen concentration between susceptible and scarcely-susceptible varieties a few weeks post inoculation, when symptoms appeared in the most susceptible varieties. On the other hand, we could not check the FD phytoplasma concentration in field grapevines, as it was not possible to find a sufficient number of FD-infected T. friulano plants, despite field surveys having been undertaken for several years.

The aim of the present work was to understand the molecular mechanisms underlying the differences in susceptibility between diverse varieties. A transcriptomic analysis was therefore conducted. Plants have shown that they coped with pathogens through two principal kinds of defense strategies: passive or pre-existing mechanisms involving structural barriers, such as waxy cuticle or reservoirs of antimicrobial compounds, and active defense mechanisms, which are induced when structural barriers are breached by pathogens [35]. In this work, for the first time, the early response of grapevine to the vector and to the FD phytoplasma carried by infective vector was evaluated, thus allowing the changes in gene expression to be studied relating to the primary mechanisms of resistance/susceptibility, and without the secondary effects caused by the development of symptoms. In addition, already-present passive mechanisms were considered, by comparing the constitutive transcriptomic profiles of the two varieties.

### Evidence for the existence of passive defense mechanisms in Tocai friulano

Plants can take advantage of several physical and chemical features to defend themselves against pathogens and herbivores. This passive defense response is due to the constitutive presence of certain structural components (such as cuticle, wax, lignin) or certain types of metabolites (such as inhibitory compounds). In this work, the differences in constitutive gene expression between the two grapevine cultivars were analyzed by comparing uninfected plants which were grown and maintained in identical controlled conditions, in order to investigate the presence of passive defense strategies.

Despite minor sequence differences, at least based on read mapping percentages and patterns and on detected SNPs on a subset of validated genes, the RNA-Seq comparative analysis and co-expression analysis revealed that the two grapevine cultivars display substantially different transcriptomic profiles especially with respect to defense-related issues (Fig. 1). Sequence annotation and classification of the DEGs according to GO categories showed that “Response to stress” and “Defense response” were the most affected, especially in the secondary metabolic pathways. These genes were preferentially more highly-expressed in T. friulano, suggesting, for this cultivar, the possible involvement of constitutive defense strategies against *S. titanus* and/or FD phytoplasma. Moreover, despite the fact that comparisons among genomes must be taken with some caution as previously discussed, RNA-Seq analysis showed higher constitutive expression levels in T. friulano of genes putatively involved in the signaling defense network responsive to pathogen/insect attacks. Indeed, many genes, coding for putative pattern recognition receptors or TIR-NB-LRR proteins and dedicated to the recognition of microbe-associated molecular patterns (*MAMPs*) or to specific effectors, in concert with some mediators of plant defense signaling (such as *SAG101*) were preferentially expressed in T. friulano. A significantly-higher expression in T. friulano was also observed for many genes involved in the JA biosynthetic pathway (Fig. 5). Jasmonic acid is considered one of the most important elicitors of plant defenses against insects, mediating the induction of several defensive compounds [48]. It is known that decreased JA levels are crucial for the success of the vector of Aster Yellow (AY) phytoplasma infestation in *Arabidopsis* [49], and also could determine feeding preference in leafhoppers, as in *Empoasca* spp*.,* which prefers to feed on *Nicotiana attenuata* mutants with reduced JA accumulation [50]. Furthermore, many genes differentially-expressed in the two varieties encode enzymes involved in the biosynthesis of secondary metabolites (Fig. 2), compounds that play a major role in defense against insects [51]. Among the secondary metabolites, plant phenols constitute one of the most common and widespread groups of defensive secondary compounds, and the accumulation of phenols is a common reaction by plants to insects intended to adversely-affect the latter’s feeding and development [52]. In this work, noSt samples in T. friulano showed an enhanced expression level of several genes involved in phenylpropanoid metabolism in comparison to Chardonnay (Fig. 5). Genes related to the biosynthetic pathway for the production of lignans, lignin and volatile benzoids were also constitutively more active in T. friulano. Lignin plays a central role in defending plants against insects and pathogens: it limits the entry of pests by blocking them physically or by increasing the leaf toughness that reduces feeding by herbivores, and also decreases the nutritional content of the leaf [51]. Finally, T. friulano showed enhanced constitutive transcript levels of several *STS* genes, suggesting a higher content of stilbenes. In some plant species these pre-exist or are synthesized after microbial attack (phytoalexins) as part of constitutive and inducible defense responses, while, especially in conifers, a role of constitutive stilbenes as a deterrent for herbivores has been reported [53–55]. Furthermore, stilbenes, which can bind covalently to cell wall components such as lignin or polysaccharides, could participate in the process of cell wall strengthening [41].

Several differences in the constitutive expression level of genes involved in secondary metabolism could also be related to the degree of attractiveness of the two grapevine cultivars for *S. titanus*. Many characteristics of host plants influence insect choices, such as leaf volatiles and colors. Plant odors are recognized to either attract or repel insect species and, in some cases, the odors from different varieties showed differing attractiveness for the insect [56, 57]. A study on potato leafhopper varietal preferences in edible beans revealed that leaf color, rather than odor, significantly influences host preference [57]. Specifically regarding *S. titanus,* several studies have provided evidence that both olfaction/olfactory and visual cues are able to affect and orientate it [58–61]. Plant-secreted volatile organic compounds (VOCs) create the plant’s “headspace”, the blend of volatiles filling the arbitrary space surrounding a plant, after they have evaporated from the plant surface. The plant volatile blends are dominated by three biosynthetic classes: terpenoids, especially mono- and sesquiterpenes, phenylalanine-derivatives, such as volatile benzoids and MeSa, and LOX-derived GLVs [62]. Here, in the comparative transcriptomic profiling of the two cultivars with contrasting behavior in the presence of FD infection, many DEGs were involved in the above biosynthetic pathways, suggesting remarkable differences in VOC emission between the two cultivars, and probably differing attractiveness for *S. titanus*. Moreover, host plant odors may interact in synergism with visual stimuli in host finding and selection. Pigments responsible for the appearance of colors in plants are classified in several groups: chlorophylls, carotenoids, flavonoids and betalains [63]. In this work the comparison of the constitutive gene expression of T. friulano and Chardonnay showed many DEGs involved in the metabolism of flavonoids.

Overall, the higher expression of several defense-related genes in T. friulano, compared to Chardonnay, together with differences in the expression level of many genes putatively involved in the differing attractiveness of the two grapevine cultivars for *S. titanus*, suggest the existence of preexisting passive defense strategies in the scarcely-susceptible cultivar, especially toward the insect.

### Activation of defense mechanisms against FDSt occurs earlier in Tocai friulano

Active defense consists of a highly-coordinated series of molecular, cellular and tissue-based barriers, erected following complex interchanges of signals triggered by plant-pathogen interaction. If defense mechanisms are not properly activated, both in terms of place and time, they will fail to restrict the pathogen and the plant will be susceptible.

In this work, T. friulano and Chardonnay showed large differences in the amplitude and type of transcriptomic changes induced by infection. Plant response to FD infection at 3 dpi was greater in the scarcely-susceptible variety, with “Cell wall metabolism” as the most highly-represented category related to plant defense (Fig. 7). Indeed, several genes involved in the metabolism of the cell wall in this cultivar were up-regulated. In parallel, a significant up-regulation of the genes involved in photosynthesis was observed in the infected T. friulano plants. The stimulation of photosynthesis could be related to the increased demand for components of the cell wall. Remodeling of the plant cell wall has frequently been reported as being part of the induced plant defense against pathogens or herbivores [64, 65] and is usually associated with the strengthening of extracellular barriers [66] or the release of signaling molecules [67]. It had been reported that cell wall reinforcements are larger and form more quickly in resistant hosts than those formed in susceptible plants [68].

Unlike T. friulano, in the present study the susceptible variety showed wider defense response to FD infection at 6 dpi (Fig. 8), mostly related to an increase in expression of several *STS* genes and several genes with high homology to plant laccases, as confirmed in the co-expression analyses. The accumulation of *laccase* transcripts could be related to the role of these enzymes in the oxidation of phenolic compounds [69]. Indeed, it could be hypothesized that in Chardonnay the increase in the transcription of several *laccases* after FD infection is a strategy to prevent cellular damage that could be caused by resveratrol accumulation following the enhanced transcription of *STS* genes. As it has been shown in other pathosystems, several susceptible hosts are not passive towards the pathogen and can set up a defense response, which is however not sufficiently effective to stop pathogen spread in infected plant tissues [70, 71]. Phytoalexins accumulate in resistant as well as in susceptible interactions, but their success against infection depends on the speed, location and magnitude of the response. Generally, susceptible plants showed delayed and diffuse phytoalexin accumulation [72, 73].

Collectively, this data showed that FD infection prompted early and substantial transcriptomic changes in the scarcely-susceptible cultivar that led to the rapid erection of active defenses, for example the remodeling of the cell wall (Figs. 7 and 19), whereas the susceptible cultivar was characterized by the later activation of defense strategies, mainly consisting of the induction of phytoalexin synthesis (Fig. 8 and Additional file 24). Thus, the differences in the type, amplitude and kinetics of gene induction during the defense response seem to be fundamental.

### FD phytoplasma modifies plant response induced by HSt feeding in chardonnay

In natural systems, co-occurring attacks by herbivorous insects and phytopathogens are frequent. However, while plant responses to insects or pathogens have been examined in some detail, their combined effects had only occasionally been considered. The combination of plant-insect and plant-pathogen interactions in one system could provide important insight into how plants prioritize and integrate signals from different suites of attacking organisms. In this study, the experimental plan including three treatments (infective vector, FD-free vector and absence of vector) at first made the above analysis of different transcriptomic changes occurring during the three-trophic interaction in the context of the two grapevine varieties possible. Additionally, the design allowed the modulation of grapevine gene expression elicited by the piercing and sucking by the infective and FD-free leafhopper *S. titanus* to be investigated for the first time*,* together with the effect induced by the FD phytoplasma in the early stages of infection, thus enabling the plant-pathogen-vector interactions at the molecular level to be dissected.

Depending on the mode of attack of the pathogen (necrotroph vs. biotroph) or the herbivore (chewing vs. piercing-sucking), plants activate different defense pathways in which the phytohormones SA, JA and ET play key roles. As crosstalk is possible between these pathways, a pathogen infection can interfere in a plant’s defense response to herbivores, and vice versa. Plant responses to herbivores are correlated to the mode of insect feeding and the degree of tissue damage at the feeding site: chewing herbivores have been largely associated with the JA-mediated response, while phloem-feeding insects, such as *S. titanus*, are often associated with the SA-mediated response and a somewhat weaker JA response [74–77]. For microbial pathogens, similar groups can be delineated: SA induces defense against biotrophic pathogens which feed and reproduce on live host cells, such as phytoplasmas, whereas JA activates defense against necrotrophic pathogens which kill host cells for nutrition and reproduction [78].

Differentiation between the plant response elicited by the FD-free vector and the response produced by the interaction between the pathogen and the infective insect showed that, at 3 dpi, wide transcriptomic changes occurred in Chardonnay in response to the FD-free insect, while the defense response resulting from the plant-pathogen-insect interaction, which has been described above, appeared much more limited. Moreover, the analyses of the different metabolic pathways highlighted the fact that the FD phytoplasma even reverts the Chardonnay response induced by HSt feeding (Figs. 7 and 8).

Indeed, the clear activation of several signaling pathways, observed in response to FD-free insect feeding, was not detected after FDSt infection. Unlike most of the phloem feeder insects, such as whiteflies and aphids, which frequently activate the SA signaling pathway [79, 80], data reported in the present study revealed that the response of Chardonnay to HSt feeding includes the modulation of genes involved in the JA/ET mediated pathways (Figs. 7 and 8). Indeed, a significant up-regulation was detected both of several genes associated with JA or ET biosynthetic processes and of genes encoding the transcription factor of several families, such as AP2/ERF, WRKY, NAC, MYB and DOF, related with JA or ET-mediated defense response. Moreover, an induction of several genes involved in the ABA-controlled signaling and biosynthesis was also noticed. The activation of both ABA and JA/ET signaling pathways has been demonstrated to contribute significantly to resistance against insects [81]. Early events following biotic stress include an immediate and dramatic calcium influx limited to a few cell layers near the damaged zone, which could be triggered by oral secretions associated with herbivore feeding [82]. Indeed, an up-regulation was observed of a number of genes coding for CMLs, which were reported to be involved in several signaling processes induced by both abiotic as well as biotic stress factors [34]. In *Arabidopsis*, multiple *CML* genes were induced by insect-derived oral secretions, indicating important functions carried out by CMLs in plant defense against insect herbivores [83]. The activation of calcium-mediated signaling can also be presumed in Chardonnay after the feeding of HSt. On the other hand, the transcriptomic profile which resulted from Chardonnay-St-phytoplasma interaction was very divergent in comparison with that induced by HSt feeding (Figs. 7 and 8). A slight modulation in *RLKs* expression level was observed, while the transcripts of genes involved in calcium signaling decreased. As regards hormone biosynthesis and signaling, few DEGs were identified after FDSt attack, which excluded the activation of ET/JA and ABA signaling pathways described above in the Chardonnay response to HSt treatment. This is consistent with the role of phytoplasma effectors SAP11 and ATP00189, which had been proved to bind to TCP proteins and down-regulate JA biosynthesis in phytoplasma-infected *Arabidopsis* and apple, respectively [49, 84]. Actually, SAP-like proteins are present in the FD phytoplasma genome and are expressed in the transcriptome of the susceptible variety Barbera [85], thus the down-regulation of the JA-pathway (Fig. 8, Additional file 20), could be associated to these phytoplasma effectors. One of the most studied effect of the down-regulation of the JA-pathway is to enhance insect vector reproduction, as demonstrated in the pathosystem *Macrosteles quadrilineatus – Arabidobpsis thaliana* – AY phytoplasma [49]. At 3 dpi a down-regulation of the genes related to the JA-pathway was also observed in T. friulano after FDSt infestation, probably as the first result of phytoplasma effectors; however, at 6 dpi an up-regulation followed, suggesting an active response of T. Friulano, which re-established the pre-infection transcriptomic levels (Fig. 9). It would be possible that decreased JA levels increase insect feeding stress in Chardonnay, leading to higher susceptibility and further symptom development. Recently, an association between the presence of *Bois noir* phytoplasma in grapevine and the activation of the SA signaling pathway, although ineffective against the disease, was reported, that seems to antagonize the JA-mediated defense response. On the opposite, an activation of the entire JA signaling pathway was detected in phytoplasma recovered plants, suggesting the importance of JA-defense response in preventing *Bois noir* phytoplasma infection and the development of the disease [86]. Overall, these data suggested the importance of decreased JA levels for the success of phytoplasma infection, and vice versa.

The activation or suppression of different signaling pathways, which is triggered by the FD-free or infective insect, led to the activation of various downstream defenses which are highly specific for particular plant-attacker combinations. Signaling pathways are interconnected and the crosstalk between them gives plants the opportunity to fine-tune and prioritize their defense in response to specific attackers [87]. In the present work, the plant defense induced by HSt feeding involved the reinforcement of the cell wall by means of the enhanced transcription of cell wall biosynthetic enzymes (Fig. 7). The up-regulation of genes that reinforce cell wall structure is thought to deter phloem-feeding insects both locally and systemically by strengthening barriers against insects [65]. This kind of reinforcement of the cell wall, induced by St feeding, was lower in the presence of the phytoplasma. Conversely, the 6 dpi response of Chardonnay, specifically activated after FDSt feeding, consisted of the induction of several genes of the general phenylpropanoid pathway and of several genes encoding stilbene synthases, responsible for stilbene biosynthesis, which were not induced by the FD-free vector. In grapevine leaves, the presence of several stilbenes had been reported to be induced by *Plasmopara viticola* and *Botrytis cinerea* attacks and in the grapevine-virus compatible interaction [88–90]. Moreover, HSt induced the up-regulation of genes encoding enzymes for the synthesis of the terpenoid delta-cadinene, a component of the plant-volatile blend together with GLVs. Plants indirectly defend themselves from herbivore feeding by emitting a blend of volatile compounds, which are specific for a particular insect-plant system and play an important role in plant defense by either attracting the natural enemies of the herbivores or by acting as a feeding and/or oviposition deterrent [51]. Several reports indicate that this sesquiterpene is among the main constituents of essential oil which is active as a repellent or insecticide [91, 92]. Surprisingly, most of these genes were not differentially-expressed after FDSt feeding.

## Conclusions

Collectively, the data reported in this work indicates that FD phytoplasma provides signals that allow for the repression of the JA/ET-mediated response induced by St feeding. Thus, it is reasonable to suppose that the FD phytoplasma enhances its success on Chardonnay plants by suppressing the effective JA-regulated defenses induced by St feeding. On the opposite, we could speculate that the constitutive high level of the JA/ET gene pathways in T. friulano, together with the other constitutive and induced defense responses observed, could help in making the latter variety less susceptible to the vector colonization and feeding, and finally also to the disease. However, in-depth further studies on the detailed mechanisms of susceptibility/resistance of grapevine to FD phytoplasma and its vector are necessary to confirm these suggestions, and to find out feasible solutions in view of a more sustainable viticulture.

## Methods

### Experimental design

The design included 12 experimental conditions (Table 3). Two grapevine cultivars, characterized by very differing susceptibility to GY, were chosen: Tocai friulano clone ISV6, which is scarcely susceptible to FD, and Chardonnay clone R8, which is very susceptible, both were obtained from CREA Research Centre for Viticulture and Enology field ampelographic collection [10, 93]. Ex vitro micropropagated plantlets from the two varieties were exposed to infective vectors (FDSt treatments), to FD-free vectors (HSt treatments) or grown in the absence of the vectors (noSt treatments). Two time points were established for the analyses: 3 and 6 days post infestation (dpi).

**Table 3:** Experimental plan: cultivars, collection time and experimental treatments

	Cultivar	Tocai friulano		Chardonnay	
	Time point	3 dpi	6 dpi	3 dpi	6 dpi
Treatment	**FD-infected St**	To_FDSt_3dpi	To_FDSt_6dpi	Cha_FDSt_3dpi	Cha_FDSt_6dpi
	**FD-free St**	To_HSt_3dpi	To_HSt_6dpi	Cha_HSt_3dpi	Cha_HSt_6dpi
	**No St**	To_noSt_3dpi	To_noSt _6dpi	Cha_noSt_3dpi	Cha_noSt_6dpi

FD: *Flavescence dorée* phytoplasma; St: *Scaphoideus titanus*, vector of *Flavescence dorée*; dpi, days post infestation

Given the difficulty involved in phytoplasma transmission and the possible low infection rate, a high number of micropropagated plantlets (108) and insects (81) were used, to ensure that there were a sufficient number of suitable biological replicates for the analyses. Accordingly, 18 plantlets were used in the noSt treatments, 25 in the HSt treatments and 65 in the FDSt treatments (Additional file 2). As regards the vector, 25 specimens were used in the HSt treatments and 56 in the FDSt treatments.

### Grapevine plantlets

In vitro micropropagated plantlets from T. friulano and Chardonnay, maintained at 25 ± 1 °C under a 16:8 h photoperiod at 60 μmol m-2 s-1 cool-white light, were previously tested with ELISA and PCR diagnostic assays to exclude the presence of the most common viral grapevine pathogens, according to well-established protocols [94]. After multiplication, they were transferred to pots with sterile soil (20 min at 120 °C) and moved to a climatic chamber at 24 ± 1 °C under a 16:8 h photoperiod and 70–75% relative humidity (RH), until their use for the experimental transmission, approximately after one month.

### Insect rearing and infection

**Fig. 10:**
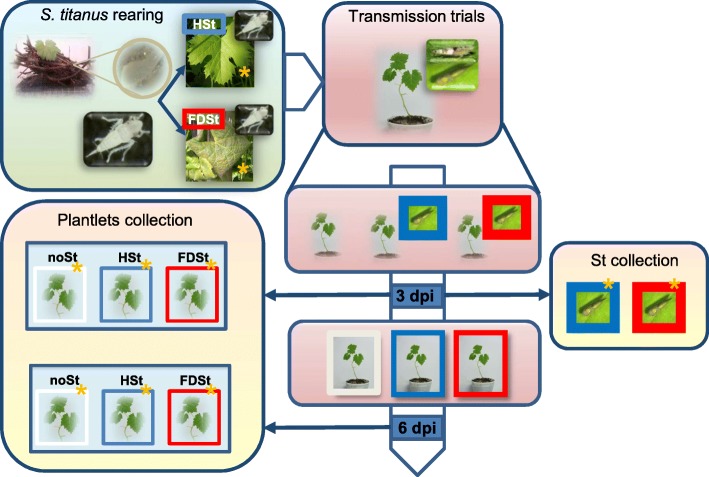
Diagram illustrating the rearing and infection of Scaphoideus titanus and the transmission trials. Hatched larvae (first instar), obtained from wood material collected from a very infested vineyard, were divided into two groups: the first was fed with grapevine leaves collected from FD-healthy grapevine plants, the second was fed with fresh FD-infected leaves from field-grown grapevine plants. For the transmission trials two S. titanus specimens (fifth instars or adults), both FD-free (HSt) or infective (FDSt), were put on every micropropagated plantlet pair and were allowed to feed for three days. Half of the plantlets were collected after three days (3 dpi), while the remaining were kept until six days (6 dpi). The asterisks indicate the points at which PCR analyses for FD phytoplasma presence were done

Egg-hatched *S. titanus* individuals, reared in cages in controlled conditions at 24 ± 1 °C, 70–75% RH and 16:8 photoperiod, were used for the transmission experiments [61]. The hatched larvae (first instar) were divided into two groups: the first was fed with fresh grapevine leaves collected from FD-healthy grapevine plants cv Pinot gris maintained in greenhouse, while the second was fed with fresh FD-infected leaves (isolate FD-D) from field-grown grapevine plants from the same cultivar, growing in a vineyard monitored since several years (Fig. 10). All the source grapevines had been previously tested by PCR to identify the FD-infected and healthy plants, as described later. Moreover, all the FD isolates present in the used plants were previously characterized by RFLP (Restriction Fragment Length Polymorphism) in the 16-23S region and by further sequencing of the *ribosomal* and *secY* genes, as described previously [95], in order to confirm their identification as FD-D phytoplasma isolate. From the infected grapevines, only leaves with clear GY symptoms were collected and used for FD acquisition. The detached leaves in the cages, maintained with the petioles immersed in a small tube with water, were checked daily and changed every 2–3 days, or earlier if they showed signals of senescence. The insects were reared in the conditions described until their use for the experimental trials.

### Transmission trials

The transmission trials were carried out with fifth instars or adults, regardless of the sex, both FD-free (fed on FD-healthy leaves) and infective (fed on FD-D-infected leaves), after two weeks of acquisition in infected leaves and 4 weeks of latency period, based on data reported in literature (Fig. 10) [5]. The micropropagated plantlets (weight 65 to 350 mg each) were placed in transparent boxes with a transpiring lid and wet substrate and separated two by two by means of a transparent wall, so that the insect movements could be monitored every day. Two *S. titanus* specimens were put on every micropropagated plantlet pair and were allowed to feed for three days. Their movements were observed daily to check whether, and where, they were feeding, and to collect the dead specimens. When both the insects preferred one of the two micropropagated plantlets, one specimen was moved to the other plant to increase the homogeneity of the treatment and the probability of FD transmission. When one or both the *S. titanus* specimens died within 24 h, one or two new specimens were added into the box. The dead specimens were frozen at − 20 °C for PCR analysis to test the presence of the FD phytoplasma. Half of the micropropagated plantlets were collected after three days (3 dpi treatment), while the remaining were kept until six days (6 dpi treatment) (Fig. 10). All the plantlets were weighed without roots and frozen at − 80 °C.

### DNA and RNA extraction

Leaf veins (1 g) from asymptomatic and symptomatic field-grown grapevine plants used for insect feeding were minced on nitrogen liquid and DNA was extracted, according to Angelini et al. [96]. DNA was extracted from the *S. titanus* specimens one by one using the same protocol.

The total RNA was extracted from 50 mg of vegetable tissue from the micropropagated plantlets employed in the trial using the plant/fungi total RNA purification kit (Norgen), then it was added to 1 μl of the RNase inhibitor RNasin Plus (Promega) to avoid it degrading, and treated with DNAse (DNA-free kit Ambion).

### PCR analyses for FD phytoplasma presence

To investigate the presence of the FD phytoplasma in insects, plants and plantlets, a real-time PCR was carried out using primers specific for FD phytoplasma, as previously described [97]. The molecular analyses were performed on DNA extracted from *S. titanus* specimens and field grapevines, while the diagnostic tests on the plantlets were carried out on the cDNA, as described later.

### Library preparation and sequencing (RNA-Seq)

The quantity and quality of RNA isolated from the micropropagated plantlets were evaluated with spectrophotometer and capillary electrophoresis (Agilent, 2100 BioAnalyzer). Only samples with a RNA Integrity Number (RIN) value higher than 7 were kept for the following analyses. Subsequently, 1.5 μg of the total RNA of each sample was used for library preparation using the Illumina TruSeq RNA library preparation kit (FC-122-1001) according to the manufacturer’s instructions. The libraries were amplified with 15 cycles of PCR and then purified and size-selected for an average size of 300 bp by a 2% low-range ultra-agarose gel (Bio-Rad). The libraries were quantified through quantitative RT-PCR (qRT-PCR), as recommended by the TruSeq RNA library preparation protocol, and single-end sequenced for 51 bases on an Illumina Genome Analyzer (GAIIx).

### RNA-Seq analyses, differentially expressed genes (DEGs) and GO enrichment methods

Following sequencing and data transfer via the 1.8 CASAVA pipeline, raw reads (Additional file 2) generated by GAIIx were checked for adapters and contaminants via FastQC application [98]. Adapters and low-quality regions were filtered out by the application of Cutadapt [99]. Filtered reads were mapped with TopHat2 and Bowtie2 [100] to the grapevine genome sequence (*Vitis vinifera* [101]; Vitis_vinifera.IGGP_12x.20). Read counts were generated from Bam alignment files with the HTSeq software [102]. Data normalization and call of differentially expressed genes (DEGs) was implemented with the DESeq2 version 1.2.8 bioconductor (R) package [103] by setting the fitting type to local and the False Discovery Rate (FDR) threshold to 0.05, and enabling independent filtering.

GO enrichment analyses were conducted using the GOseq bioconductor package. GOseq was specifically designed to minimize length-derived bias which might affect RNA-Seq data [104]. The data preparation for the GOseq analysis was as previously reported [105], apart from the use of an FDR cutoff of 0.1 for GO enrichments.

Sample clustering heatmaps were obtained upon log2-transformation [28] of DESeq2-normalized expression data.

### Bioinformatics and statistical analyses

For MapMan analysis [106], *V. vinifera* transcripts have been batch assigned to functional categories figures were obtained by running the Mercator tool [34] against the *Arabidopsis* TAIR10 protein database, the SwissProt plants database, the TIGR5 rice proteins database, the *Chlamydomonas reinhardtii* protein database, the InterPro database, the Conserved Domain Database (CDD) and the KOG database with default parameters to assign MapMan bins to *V. vinifera* transcripts. Indeed, although MapMan website (http://mapman.gabipd.org/mapmanstore) provided mapping files for *V. vinifera*, they were not updated with the gene models of the used *V. vinifera* genome release (Vitis_vinifera.IGGP_12x.20). For all the pathways potentially important in the present work, such as those related to the defense, an accurate analysis of each DEG was performed comparing manually the MapMan annotations in BLAST, and using also the GO annotations. Log2 fold changes as obtained from the DESeq2 output were used as MapMan inputs to represent expression changes. The Wilcoxon test was applied for determination of the *p*-values. To identify and to provide a statistics-based overview of the changed pathways, the DEGs were analyzed by PageMan embedded in MapMan [21]. Data were processed in PageMan using ORA analysis with Fisher’s exact test, setting a threshold of 1 (at least a twofold change).

In order to compare and summarize the responses of the two varieties at a glance in the various conditions tested, grapevine transcripts were assigned to KO (KEGG Orthology) terms through the KAAS server; the Pathview R tool package [107] was then used to allow a prompt comparative evaluation of expression responses. Boxes were placeholders for one or more genes assigned to the same KO group. When more than one gene was mapped to the same group, the expression fold-changes were summed up according to the default Pathview settings.

IGV (Integrative Genomic Viewer) figures were obtained after merging of all raw alignment BAM files (non-normalized) via SAMtools [108]. Allele frequency threshold was set to 0.4, data range sets to autoscale, and multimapper reads were filtered out from visualization by setting the mapping quality threshold to 50.

Spearman correlations were performed implementing the “cor” function as available in R “stats” package by setting the method parameter to “spearman”.

Principal Component Analyses (PCA) were performed as specified in DESeq2 reference [103] by customizing the “ggplot” function.

### Co-expression analyses

Following sub-sampling of the whole DESeq2-normalized matrix of expression data (countSet), carried out by filtering all the genes called DEGs (thresholds set at FDR 0.01) in at least one comparison, a matrix of 17,228 genes and 32 samples (including biological replicates) was generated. For this matrix, the signed network adjacency was calculated implementing the adjacency function as available in the R WGCNA package [109]. The correlation threshold was set to 0.86. Based on the adjacency matrix, a graphNEL-type graph was generated and sent via the Rcytoscape Bioconductor package [110] to the Cytoscape application version 2.8.1 [111]. Cluster visualizations and batch analysis were based on functions from the Rcytoscape package. Enriched GOs for the Biological Process (BP) domain for genes in clusters were calculated with the hypergeometric test as implemented in the Bioconductor GOstats package [112] using a *p*-value cutoff of 0.01.

### Quantitative RT-PCR (qRT-PCR)

Quantitative RT-PCR analyses were performed to calculate the FD titre in infected plantlets, to validate the RNA-Seq data and to confirm some significant results obtained with the RNA-Seq. All the qRT-PCR reactions were carried out on the cDNA obtained through the retrotranscription of 0.5 μg of extracted RNA using M-MLV reverse transcriptase (Invitrogen), in a Bio-Rad thermalcycler (model CFX96).

For the quantification of the FD phytoplasma, the constitutive 16S rRNA gene was used as a reference. Quantitative RT-PCR assays were carried out according to a previously described protocol [24]. A standard curve was established by diluting plasmid pGEM-T Easy Vector (Promega, WI, USA) containing a 1240 bp portion of the 16S rRNA gene of the FD-D phytoplasma. The plasmid was quantified by means of a spectrophotometer and then used to prepare 1:2 serial dilutions in water. The concentration (ng/μl) of the plasmid with the insert was converted to copy number; the dilutions ranged from 2.5 × 10^4^ to 50 copies. Each sample of cDNA obtained from the FD-infected plantlets was run in duplicate together with the plasmid serial dilutions, and the mean number of phytoplasma 16S rRNA copies was calculated.

In order to validate the RNA-Seq technique, qRT-PCR analyses on eight grapevine genes which showed different expression level and profiles were performed on two independent biological replicates collected at 6 dpi. Moreover, to confirm some of the most interesting results obtained with the RNA-Seq, the expression level of further seven genes, involved in the jasmonic acid (JA)-related pathway, was analyzed at least on two independent biological replicates collected at 3 and 6 dpi. Primers were designed using Primer3 software [113] (Additional file 27) and primer specificity was evaluated by blasting primer sequences against the NCBI database. Quantitative RT-PCR assays were carried out using the SensiFAST SYBR No-ROX kit (Bioline). PCR reactions were performed at least in duplicate, in a total volume of 20 μl, including 0.3 μM of each primer and 4 μl of a 1:10 dilution of cDNA. The thermal protocol included 2 min at 95 °C for polymerase activation and 40 cycles of a two-step protocol, consisting of 5 s of denaturation at 95 °C and 30 s of annealing/extension at 60 °C. Identical thermal cycling conditions were used for all targets. The performance of each qPCR assay was evaluated by generating three standard curves over a broad range of cDNA dilutions (from undiluted to 10^− 4^) with the obtainment of amplification efficiencies higher than 93% in all cases. For the relative quantification, a set of five *V. vinifera* candidate reference genes was tested in the experimental conditions, using primer pairs and qPCR conditions described previously [114]. The qbasePLUS software (Biogazelle) was used to identify the *Glyceraldehyde-3-phosphate dehydrogenase* (GAPDH) and *Cytochrome oxidase* (COX) as the two most stably-expressed genes, which were thus chosen for the normalization. The relative gene expression was calculated using the comparative Cq (2-∆∆Cq) method.

## Additional files

Additional file 1: Quantification of the Flavescence dorèe phytoplasma on the plantlets of the FDSt treatments. For each infected sample the copy numbers of Flavescence doreé phytoplasmas were calculated for μg of RNA. The very low amount of RNA of the sample Cha_FDSt_3dpi_134 did not permit us to quantify phytoplasmas on it. The average copy numbers of each thesis, together with the standard deviations, are reported. Results are not significantly different at 5% using the Student-Newman-Keuls test (a). (DOCX 15 kb)

Additional file 2: Number of biological replicates and samples discarded. Each column indicates the number of plantlets available for each condition in the different phases of the work. RIN: RNA Integrity Number. (DOCX 13 kb)

Additional file 3: All sample correlation coefficients. Pearson correlation coefficients for all samples originally sequenced are reported. (XLSX 25 kb)

Additional file 4: Clustering of all samples. Heatmaps reporting clustering of all samples were generated upon rlog-transformation of DESeq2-normalized expression data. (PNG 237 kb)

Additional file 5: Spearman correlation coefficients of all sequenced samples, according to RNA-seq ENCODE standard, 2016 [44]. Spearman correlations were calculated using the “cor” function in R “stats” package. Samples highlighted in colours have been subsequently removed from downstream steps, based on the above Pearson correlations as well as sample clustering after Rlog transformation of data. (XLSX 27 kb)

Additional file 6: Principal Component Analysis (PCA) of all sequenced sample. Care was taken to identify univocally all samples by marking each one with a specific symbol, as listed in the legend. The treatments are specified by colour-coding of the above symbols. Removed samples are labelled. (PNG 596 kb)

Additional file 7: Selected sample correlation coefficients. Pearson correlation coefficients for samples retained in all analyses are reported. (XLSX 20 kb)

Additional file 8: Clustering of selected samples. Heatmaps reporting sample clustering of non-discarded samples retained in analyses were generated upon rlog-transformation of DESeq2-normalized expression data. (PNG 215 kb)

Additional file 9: Read mapping details. Total number of input reads and read mapping details, based on TopHat2 output reports, are shown for the two varieties. (XLSX 14 kb)

Additional file 10: IGV read mapping patterns on validation set genes. Read mapping patterns over Pinot noir reference for the eight genes of the validation set (ID on the bottom of each panel) for Tocai friulano and Chardonnay. All alignment files for all conditions were separately merged for T. friulano and Chardonnay and loaded on IGV (Integrative Genome Viewer). Mapped reads are colored in red or blue depending on their mapping on sense or antisense strand, respectively. Above each read mapping row, a further row summarizes the total amount of mapping reads (autoscaled to best fit data). Colored spikes in this same row (the actual color depends on base involved) indicate SNPs, which are reported in case that at least 40% of the reads shares the SNP. (PNG 1591 kb)

Additional file 11: Differentially-expressed genes in the comparisons between Chardonnay and Tocai friulano at a constitutive level and after HSt and FDSt treatments. RNA-Seq results with DEGs obtained in the comparisons between “Cha_HSt vs. To_HSt”, “Cha_FDSt vs. To_FDSt” and “Cha_noSt vs. To_noSt” cultivars at 3 and 6 days post infestation. (XLSX 6099 kb)

Additional file 12: Enriched GO terms in the comparisons between Chardonnay and Tocai friulano at a constitutive level and after HSt and FDSt attacks. Results of GO enrichment analysis obtained in the comparisons between “Cha_HSt vs. To_HSt”, “Cha_FDSt vs. To_FDSt” and “Cha_noSt vs. To_noSt” cultivars at 3 and 6 days post infestation. (XLSX 54 kb)

Additional file 13: Differentially-expressed genes in Chardonnay after HSt and FDSt attacks. RNA-Seq results with DEGs obtained in the “noSt vs. HSt” and “noSt vs. FDSt” comparisons in Chardonnay at 3 and 6 days post infestation. (XLSX 1075 kb)

Additional file 14: Differentially-expressed genes in Tocai friulano after HSt and FDSt attacks. RNA-Seq results with DEGs obtained in the “noSt vs. HSt” and “noSt vs. FDSt” comparisons in Tocai friulano at 3 and 6 days post infestation. (XLSX 1353 kb)

Additional file 15: Enriched GO terms in Chardonnay after HSt and FDSt attacks. Results of GO enrichment analysis obtained in the “noSt vs. HSt” and “noSt vs. FDSt” comparisons in Chardonnay at 3 and 6 days post infestation. (XLSX 54 kb)

Additional file 16: Enriched GO terms in Tocai friulano after HSt and FDSt attacks. Results of GO enrichment analysis obtained in the “noSt vs. HSt” and “noSt vs. FDSt” comparisons in Tocai friulano at 3 and 6 days post infestation. (XLSX 48 kb)

Additional file 17: Venn diagrams illustrating the relationship between the DEGs in the different treatments and the overlap between up-regulated and down-regulated DEGs. Twenty Venn diagrams are reported, grouped in five different figures; each figure includes two pairwise comparisons on the top, and the overlap between up-regulated and down-regulated DEGs on the bottom. (PDF 791 kb)

Additional file 18: RNA-Seq validation by qRT-PCR. Fold change values obtained by means of qRT-PCR and RNA-Seq are plotted on an x and y axis. Log fold change values were calculated for 15 selected genes considering different comparisons (Cha_noSt vs. HSt_3dpi, Cha_ noSt vs. FDSt_3dpi; Cha_noSt vs. HSt_6dpi; Cha_noSt vs. FDSt_6dpi; To_noSt vs. HSt_3dpi; To_noSt vs. FDSt_3dpi; To_noSt vs. HSt_6dpi; To_noSt vs. FDSt_6dpi; Cha vs. To_noSt_3dpi; Cha vs. To_noSt_6dpi; Cha vs. To_HSt_3 dpi; Cha vs. To_HSt_6 dpi; Cha vs. To_FDSt_3 dpi; Cha vs. To_FDSt_6 dpi), for a total of 129 comparisons. The correlation coefficient (R^2^) is reported. (PNG 60 kb)

Additional file 19: Global overview of the constitutive transcriptomic differences between Chardonnay and Tocai friulano. Mean expression versus log fold change-plots (MA-plot) computed for noSt treatments of Chardonnay and T. friulano. Normalized expression mean values are plotted versus log2 fold changes and the DEGs (FDR < 0.05) are plotted in red. (PNG 73 kb)

Additional file 20: Details of Mapman Wilcoxon’s rank sum test reporting the *p*-values of different bins modulated in Chardonnay and represented in Fig. 9. Data concerning bin number and name, the number of DEGs per bin, and the *p*-values are reported after HSt and FDSt treatments at 3 and 6 dpi. (XLSX 88 kb)

Additional file 21: Details of Mapman Wilcoxon’s rank sum test reporting the *p*-values of different bins modulated in Tocai friulano and represented in Fig. 10. Data concerning bin number and name, the number of DEGs per bin, and the *p*-values are reported after HSt and FDSt treatments at 3 and 6 dpi. (XLSX 99 kb)

Additional file 22: Expression patterns of genes involved in different pathways in Chardonnay: A) signaling pathways, B) hormone-related pathways, C) transcription factors, D) antioxidant mechanisms, E) cell wall, F) defense proteins, G) secondary metabolism, H) primary metabolism. From left to right, the four columns show the expression of DEGs modulated by HSt and FDSt at 3 and 6 days post infestation, respectively. Every row represents a different gene. The green, black and red colors indicate the log2 fold changes, low, medium and high, respectively, obtained from DESeq2 with FDR < 0.05. Log2 fold changes of genes that were not considered differentially regulated in a specific comparison due to a FDR above the threshold were arbitrary set to 0. (PDF 2356 kb)

Additional file 23: Expression patterns of genes involved in different pathways in Tocai friulano: A) signaling pathways, B) hormone-related pathways, C) transcription factors, D) antioxidant mechanisms, E) cell wall, F) defense proteins, G) secondary metabolism, H) primary metabolism. From left to right, the four columns respectively show the expression of DEGs modulated by HSt and FDSt at 3 and 6 days post infestation. Every row represents a different gene. The green, black and red colors indicate the log2 fold changes, respectively low, medium and high, obtained from DESeq2 with FDR < 0.05. For genes that were not considered differentially regulated in a specific comparison due to a FDR above the threshold, their log2 fold change have been arbitrary set to 0. (PDF 2755 kb)

Additional file 24: Details of differentially expressed genes involved in hormone-related pathways, cell wall and secondary metabolism modulated by HSt and FDSt at 3 and 6 days post infestation in Chardonnay and Tocai friulano. The bin numbers and names, the id numbers and the description of the genes, and the Log2 fold changes, as obtained from the DESeq2 output, are reported. (XLSX 176 kb)

Additional file 25: Co-expression analysis results: annotation details for genes identified in each cluster. ID, name, annotation and description of each gene, together with GO annotation, are reported for each cluster. (XLSX 78 kb)

Additional file 26: Co-expression analysis results: enriched GO terms identified for each cluster. Enriched GOs for the Biological Process domain for genes in clusters were calculated with the hypergeometric test as implemented in the Bioconductor GOstats package, using a *p*-value cutoff of 0.01. (XLSX 64 kb)

Additional file 27: Sequences of primer pairs designed and used in qRT-PCR for each target gene. Above, primers used for the validation of RNA-Seq technique; below, primers used for the validation of RNA-Seq results, concerning the jasmonate pathway. (XLSX 14 kb)

## Data Availability

All data generated or analysed during this study are included in this published article and its supplementary information files. FastQ files of RNA-Seq reads have been loaded into ArrayExpress with accession E-MTAB-7710.

## Abbreviations

4CL: 4-coumarate-CoA ligase
AAO: ABA-aldehyde oxidase
ABA: Abscisic acid
ACC: 1-aminocyclopropane-1-carboxylic acid
ACO: 1-aminocyclopropane-1-carboxylic oxidase
ACS: 1-aminocyclopropane-1-carboxylic synthase
AP2/ERF: APETALA2/ethylene-responsive factor
APX: Ascorbate peroxidase
ATP: Adenosine triphosphate
ATPase: Adenosine triphosphatase
AY: Aster Yellow
BAM: Binary Alignment Map
BP: Biological Process
C4H: Cinnamate 4-hydroxylase
CAD: Cinnamyl alcohol dehydrogenase
CCoAOMT: Caffeoyl-CoA 3-O-methyltransferase
CCR: Cinnamoyl-CoA reductase
CDD: Conserved Domain Database
cDNA: Complementary DNA
CDS: Coding sequence
Cha: Chardonnay
CMLs: Calmodulin-like proteins
COX: Cytochrome oxidase
Cq: Quantification cycle
DEGs: Differentially expressed genes
DNAse: Deoxyribonuclease
dpi: Days post infestation
ELISA: Enzime-linked immunosorbent assay
ENCODE: Encyclopedia of DNA Elements
ERF: Ethylene response factor
ET: Ethylene
F5H: Ferulate-5-hydroxylase
FD: Flavescence dorée
FDR: False Discovery Date
FDSt: Response to the three-trophic grapevine-phytoplasma-insect interaction
GAPDH: Glyceraldehyde 3-phosphate dehydrogenase
GLV: Green leaf volatiles
GO: Gene Ontology
GST: Glutathione S-transferase
GST: Glutathione S-transferase
GY: Grapevine yellows
HHT: O-feruloyl transferase
HSt: Grapevine response to *Scaphoideus titanus* piercing and sucking
IGV: Integrative Genomic Viewer
JA: Jasmonic acid
JAMT: Methiltransferase
KAAS: KEGG Automatic Annotation Server
KEGG: Kyoto Encyclopedia of Genes and Genomes
KO: KEGG Orthology
LOX: Lipoxygenase
LRR: Leucine-rich repeat
MAMPs: Microbe-associated molecular patterns
MEP: 2-C-methylerythritol 4-phosphate
MeSA: Gaseous methyl salicylate
M-MLV: Moloney Murine Leukemia Virus
mRNA: Messenger RNA
NB: Nucleotide binding
NCBI: National Center for Biotechnology Information
NCED: 9-cis-epoxycarotenoid dioxygenase
OPR: Oxo-phytodienoic acid reductase
ORA: Over-representation analysis
PAL: Phenylalanine ammonia lyase
PCA: Principal Component Analysis
PCR: Polymerase Chain Reaction
POD: Peroxidase
PR proteins: Pathogenesis-related proteins
PRX: Peroxiredoxin
qRT-PCR: Quantitative Reverse Transcriptase - Polymerase Chain Reaction
RFLP: Restriction Frangment Lenght Polymorphism
RH: Relative Humidity
RIN: RNA Integrity Number
RLKs: Receptor-like kinases
RNase: Ribonuclease
RNA-Seq: RNA sequencing
ROS: Reactive oxygen species
rRNA: Ribosomal RNA
RT-PCR: Reverse Transcriptase- Polymerase Chain Reaction
SA: Salicylic acid
SAMT: Salicylic acid methyltransferase
SAMtools: Sequence Alignment/Map tools
SNPs: Single nucleotide polymorphisms
SOD: Superoxide dismutase
SSR: Simple Sequence Repeat
St: Scaphoideus titanus
STS: Stilbene synthase
T. friulano: Tocai friulano
TIR: Toll-Interleukin receptor
To: Tocai friulano
TPSs: Terpene synthases
VOCs: Volatile organic compounds
WAKs: Cell wall-associated-like kinases
WGCNA: Weighted correlation network analysis
XTH: Xyloglucan endotransglycosylase
