# Supplementary material for: Grapevine comparative early transcriptomic profiling suggests that Flavescence dorée phytoplasma represses plant responses induced by vector feeding in susceptible varieties
Source: BMC Genomics. 2019 Jun 26;20:526. doi: 10.1186/s12864-019-5908-6 (PMC6595628; doi:10.1186/s12864-019-5908-6)
Supplement: Supplementary file 1 — Quantification of the Flavescence dorèe phytoplasma on the plantlets of the FDSt treatments. For each infected sample the copy numbers of Flavescence doreé phytoplasmas were calculated for μg of RNA. The very low amount of RNA of the sample Cha_FDSt_3dpi_134 did not permit us to quantify phytoplasmas on it. The average copy numbers of each thesis, together with the standard deviations, are reported. Results are not significantly different at 5% using the Student-Newman-Keuls test (a). (DOCX 15 kb) [file 12864_2019_5908_MOESM1_ESM.docx]

| **Sample id** | **Copy numbers for μg of RNA** | **Average** | **Standard deviation** |
| --- | --- | --- | --- |
| **Cha_FDSt_3dpi_50** | 37263 | 19255 (a) | 25468 |
| **Cha_FDSt_3dpi_132** | 1246 |  |  |
| **Cha_FDSt_6dpi_662** | 8845 | 7262.7 (a) | 1780.9 |
| **Cha_FDSt_6dpi_668** | 5334 |  |  |
| **Cha_FDSt_6dpi_670** | 7609 |  |  |
| **To_FDSt_3dpi_80** | 11443 | 8351 (a) | 4372.7 |
| **To_FDSt_3dpi_82** | 5259 |  |  |
| **To_FDSt_6dpi_100** | 7886 | 5623 (a) | 32004 |
| **To_FDSt_6dpi_101** | 3360 |  |  |
