# Supplementary material for: Grapevine comparative early transcriptomic profiling suggests that Flavescence dorée phytoplasma represses plant responses induced by vector feeding in susceptible varieties
Source: BMC Genomics. 2019 Jun 26;20:526. doi: 10.1186/s12864-019-5908-6 (PMC6595628; doi:10.1186/s12864-019-5908-6)
Supplement: Supplementary file 2 — Number of biological replicates and samples discarded. Each column indicates the number of plantlets available for each condition in the different phases of the work. RIN: RNA Integrity Number. (DOCX 13 kb) [file 12864_2019_5908_MOESM2_ESM.docx]

| **Treatment name** | **No. of biological replicates** | | | | | |
| --- | --- | --- | --- | --- | --- | --- |
|  | **Used for transmission** | **Extracted *** | **After sanitary evaluation **** | **After RNA quality assessment (RIN>7)** | **Sequenced** | **After RNA-Seq analyses** |
| To_FDSt_3dpi | 16 | 8 | 3 | 3 | 3 | 2 |
| To_HSt_3dpi | 8 | 5 | 5 | 4 | 3 | 3 |
| To_ noSt_3dpi | 5 | 5 | 5 | 5 | 5 | 3 |
| To_FDSt_6dpi | 18 | 11 | 3 | 2 | 2 | 2 |
| To_HSt_6dpi | 5 | 3 | 3 | 3 | 3 | 3 |
| To_ noSt_6dpi | 4 | 3 | 3 | 3 | 3 | 3 |
| Cha_FDSt_3dpi | 17 | 8 | 5 | 5 | 5 | 3 |
| Cha _HSt_3dpi | 5 | 5 | 5 | 4 | 3 | 2 |
| Cha_ noSt_3dpi | 5 | 3 | 3 | 3 | 3 | 3 |
| Cha _FDSt_6dpi | 14 | 5 | 3 | 3 | 3 | 3 |
| Cha _HSt_6dpi | 7 | 5 | 5 | 4 | 3 | 3 |
| Cha_noSt_6dpi | 4 | 4 | 4 | 3 | 3 | 2 |

* For FDSt treatments, only plantlets where at least one of the two insects have been found infected were analysed

** For FDSt treatments, only FD-infected samples were retained
