# Supplementary figures and images for: Grapevine comparative early transcriptomic profiling suggests that Flavescence dorée phytoplasma represses plant responses induced by vector feeding in susceptible varieties
Source: BMC Genomics. 2019 Jun 26;20:526. doi: 10.1186/s12864-019-5908-6 (PMC6595628; doi:10.1186/s12864-019-5908-6)

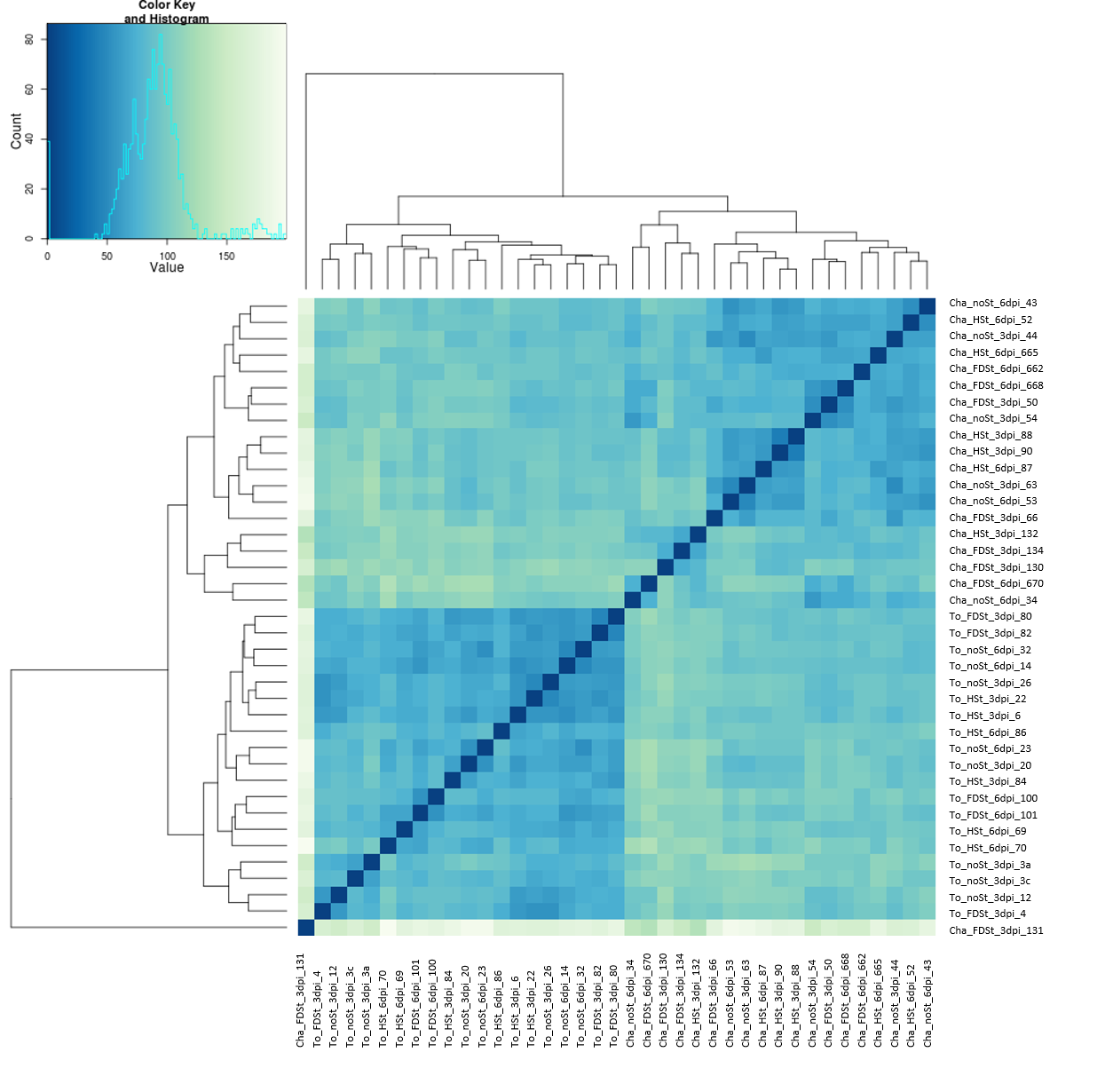

Supplement: Supplementary file 4 — Clustering of all samples. Heatmaps reporting clustering of all samples were generated upon rlog-transformation of DESeq2-normalized expression data. (PNG 237 kb) [file 12864_2019_5908_MOESM4_ESM.png]

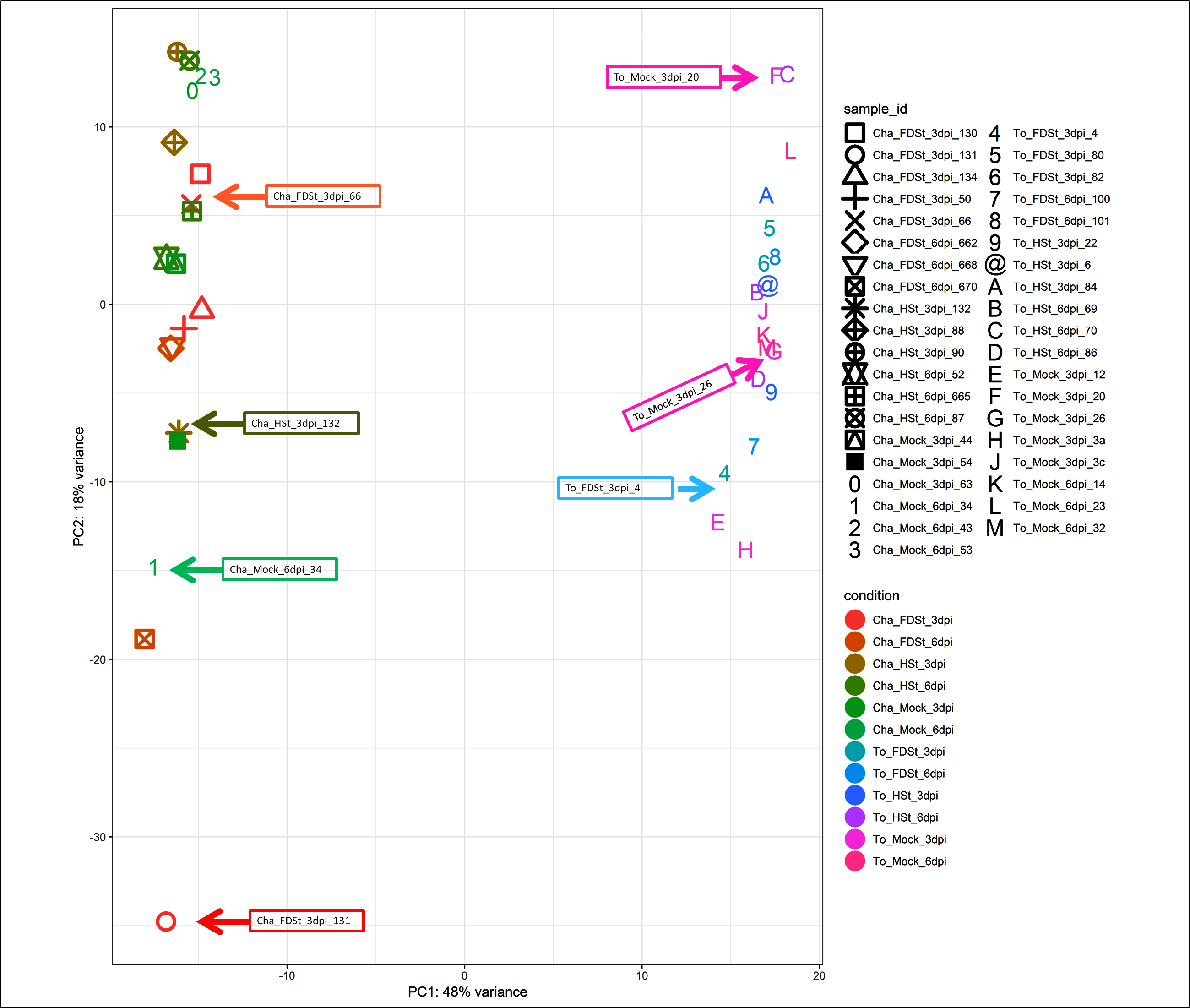

Supplement: Supplementary file 6 — Principal Component Analysis (PCA) of all sequenced sample. Care was taken to identify univocally all samples by marking each one with a specific symbol, as listed in the legend. The treatments are specified by colour-coding of the above symbols. Removed samples are labelled. (PNG 596 kb) [file 12864_2019_5908_MOESM6_ESM.png]

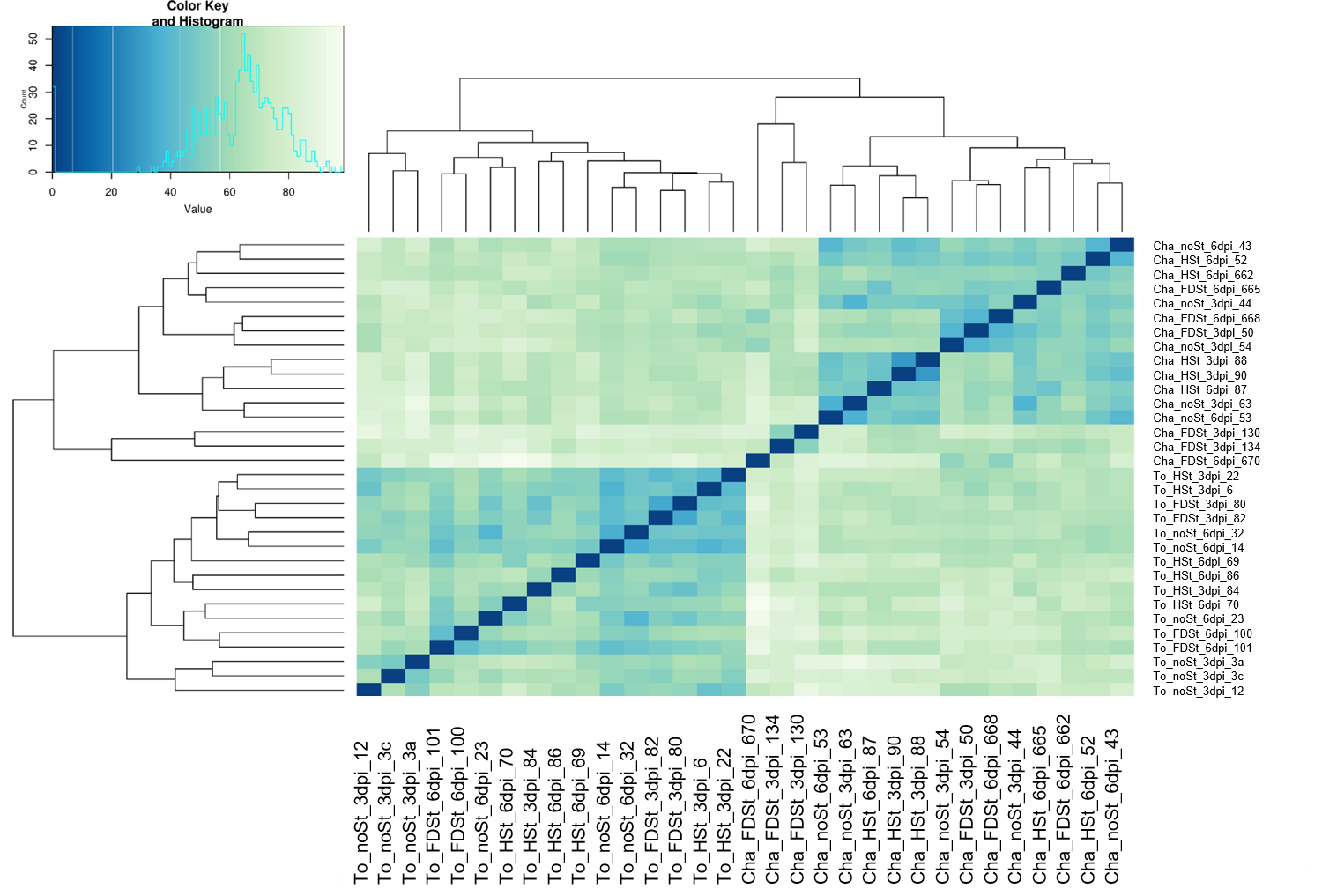

Supplement: Supplementary file 8 — Clustering of selected samples. Heatmaps reporting sample clustering of non-discarded samples retained in analyses were generated upon rlog-transformation of DESeq2-normalized expression data. (PNG 215 kb) [file 12864_2019_5908_MOESM8_ESM.png]

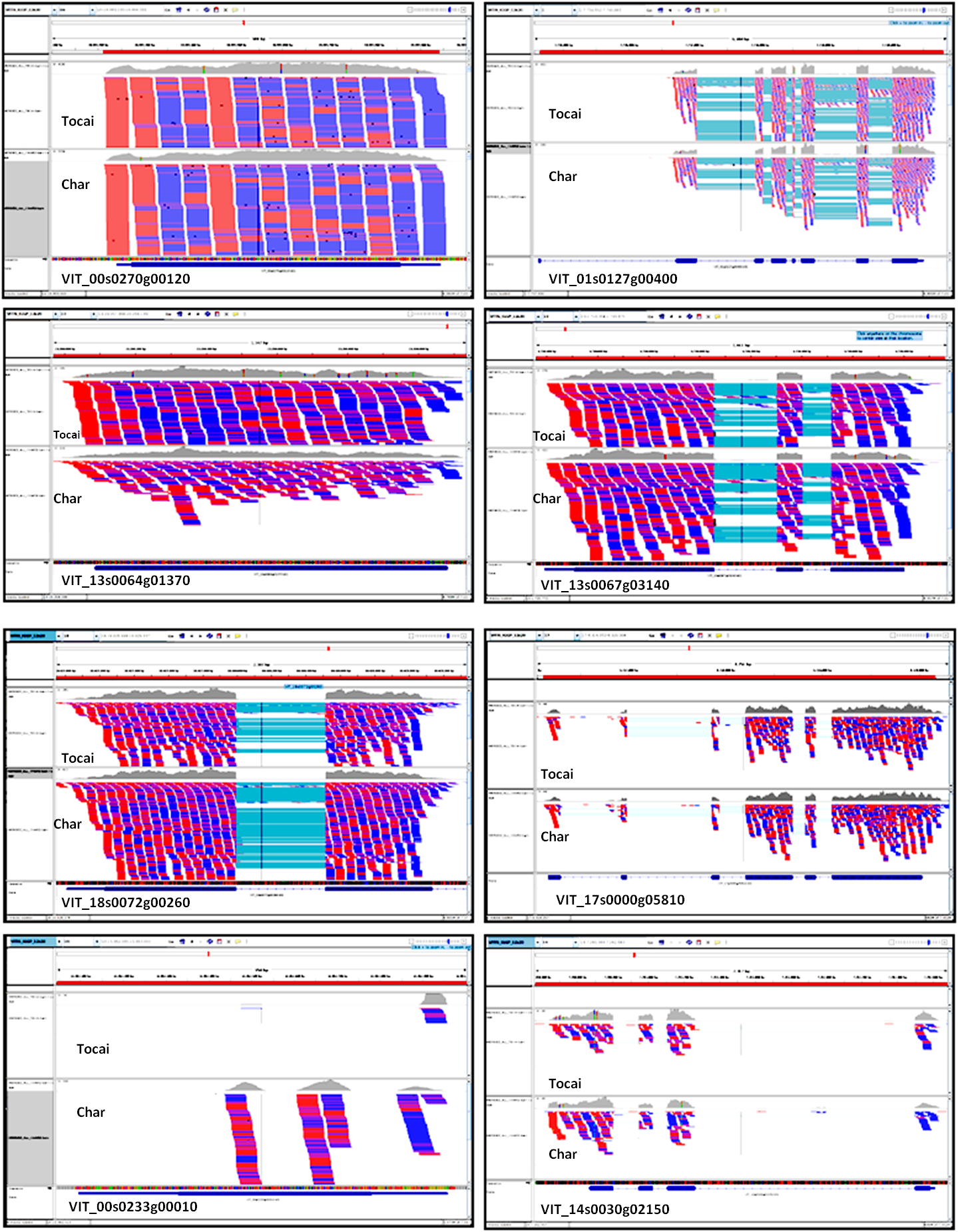

Supplement: Supplementary file 10 — IGV read mapping patterns on validation set genes. Read mapping patterns over Pinot noir reference for the eight genes of the validation set (ID on the bottom of each panel) for Tocai friulano and Chardonnay. All alignment files for all conditions were separately merged for T. friulano and Chardonnay and loaded on IGV (Integrative Genome Viewer). Mapped reads are colored in red or blue depending on their mapping on sense or antisense strand, respectively. Above each read mapping row, a further row summarizes the total amount of mapping reads (autoscaled to best fit data). Colored spikes in this same row (the actual color depends on base involved) indicate SNPs, which are reported in case that at least 40% of the reads shares the SNP. (PNG 1591 kb) [file 12864_2019_5908_MOESM10_ESM.png]

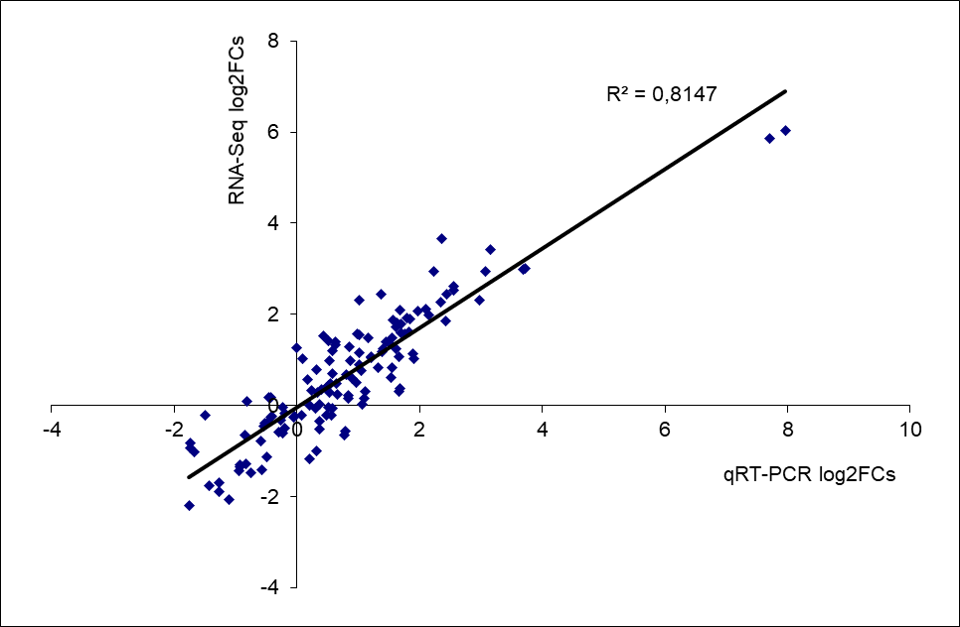

Supplement: Supplementary file 18 — RNA-Seq validation by qRT-PCR. Fold change values obtained by means of qRT-PCR and RNA-Seq are plotted on an x and y axis. Log fold change values were calculated for 15 selected genes considering different comparisons (Cha_noSt vs. HSt_3dpi, Cha_ noSt vs. FDSt_3dpi; Cha_noSt vs. HSt_6dpi; Cha_noSt vs. FDSt_6dpi; To_noSt vs. HSt_3dpi; To_noSt vs. FDSt_3dpi; To_noSt vs. HSt_6dpi; To_noSt vs. FDSt_6dpi; Cha vs. To_noSt_3dpi; Cha vs. To_noSt_6dpi; Cha vs. To_HSt_3 dpi; Cha vs. To_HSt_6 dpi; Cha vs. To_FDSt_3 dpi; Cha vs. To_FDSt_6 dpi), for a total of 129 comparisons. The correlation coefficient (R2) is reported. (PNG 60 kb) [file 12864_2019_5908_MOESM18_ESM.png]

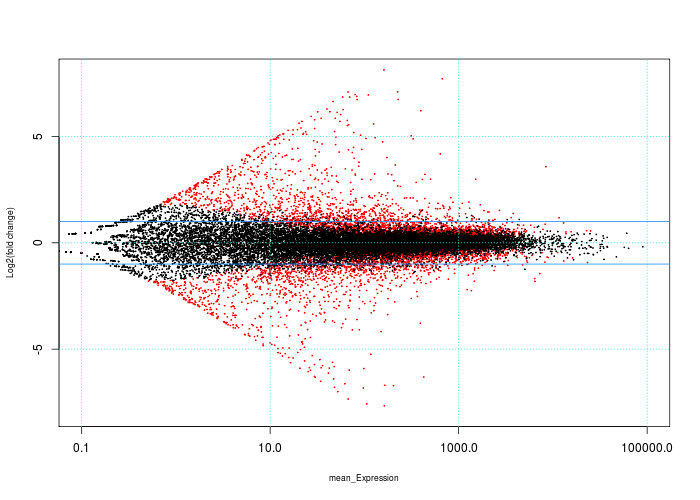

Supplement: Supplementary file 19 — Global overview of the constitutive transcriptomic differences between Chardonnay and Tocai friulano. Mean expression versus log fold change-plots (MA-plot) computed for noSt treatments of Chardonnay and T. friulano. Normalized expression mean values are plotted versus log2 fold changes and the DEGs (FDR < 0.05) are plotted in red. (PNG 73 kb) [file 12864_2019_5908_MOESM19_ESM.png]
