# Supplementary material for: Grapevine comparative early transcriptomic profiling suggests that Flavescence dorée phytoplasma represses plant responses induced by vector feeding in susceptible varieties
Source: BMC Genomics. 2019 Jun 26;20:526. doi: 10.1186/s12864-019-5908-6 (PMC6595628; doi:10.1186/s12864-019-5908-6)

1) Venn diagrams of genes differentially expressed between the two grapevine cultivars at 3 and 6 days after HSt and FDSt attacks. Venn diagrams illustrating the relationship between the DEGs detected at 3 and 6 days post infestation in HSt (**A on the top**) and FDSt (**B on the top**) and the overlap between up-regulated and down-regulated DEGs (**A and B on the bottom**).

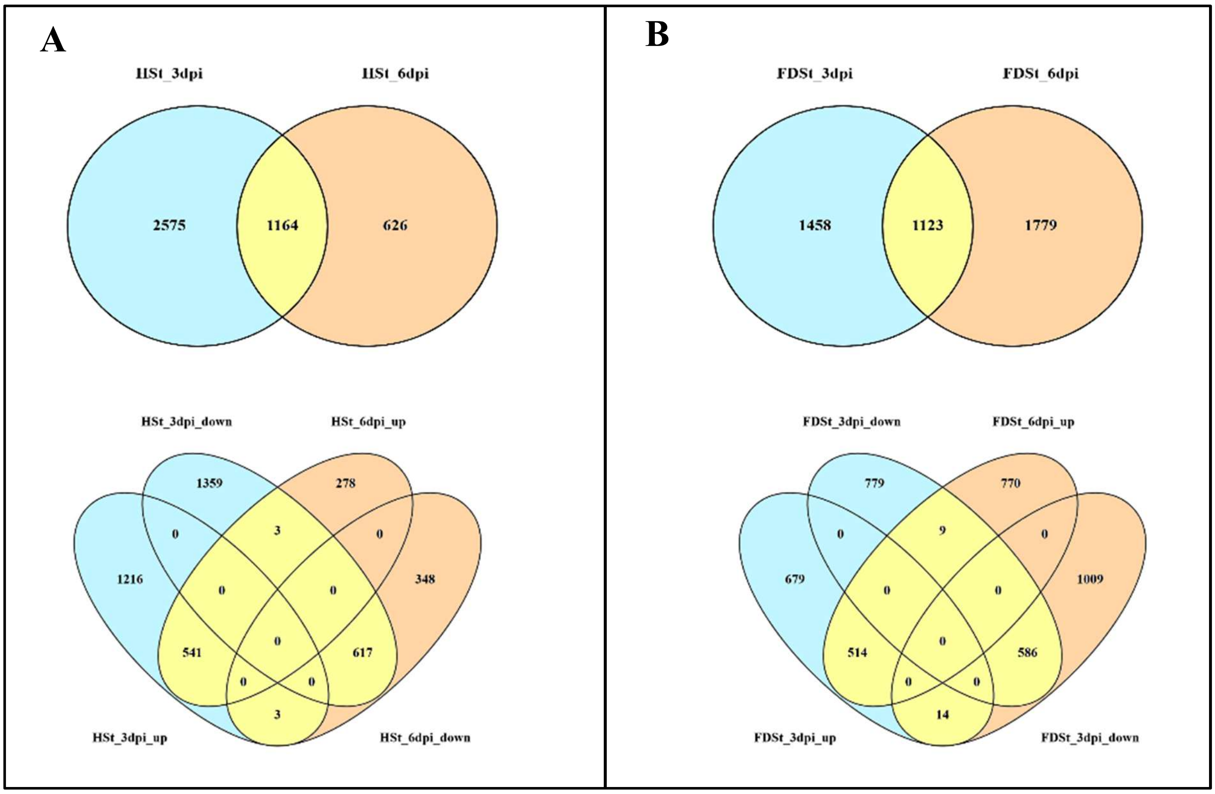

2) Venn diagrams of genes genes differentially expressed between the two grapevine cultivars after HSt and FDSt attacks at two time points. Venn diagrams illustrating the relationship between the DEGs modulated after HSt and FDSt attacks at 3 (**A on the top**) and 6 days post infestation (**B on the top**) and the overlap between up-regulated and down-regulated DEGs (**A and B on the bottom**).

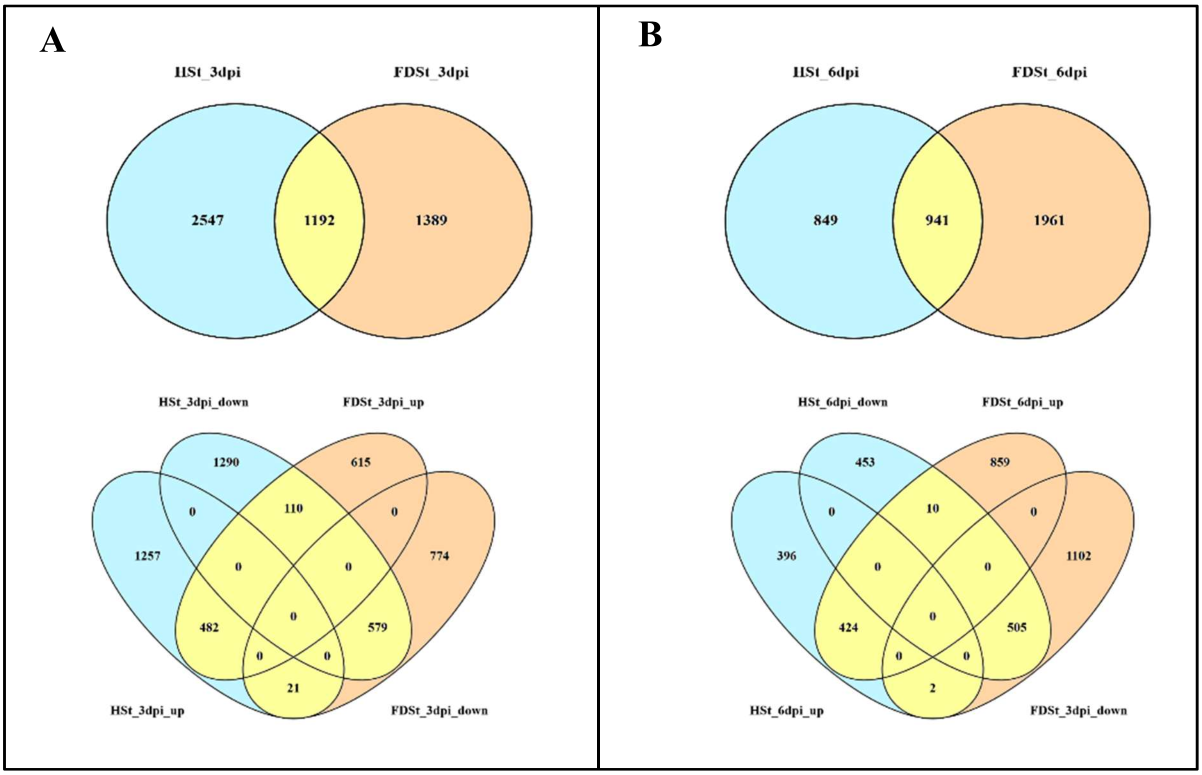

3) Venn diagrams of genes modulated at 3 and 6 days after HSt and FDSt attacks in Chardonnay. Venn diagrams illustrating the relationship between the DEGs detected at 3 and 6 days post infestation in HSt (**A on the top**) and FDSt (**B on the top**) and the overlap between up-regulated and down-regulated DEGs (**A and B on the bottom**).

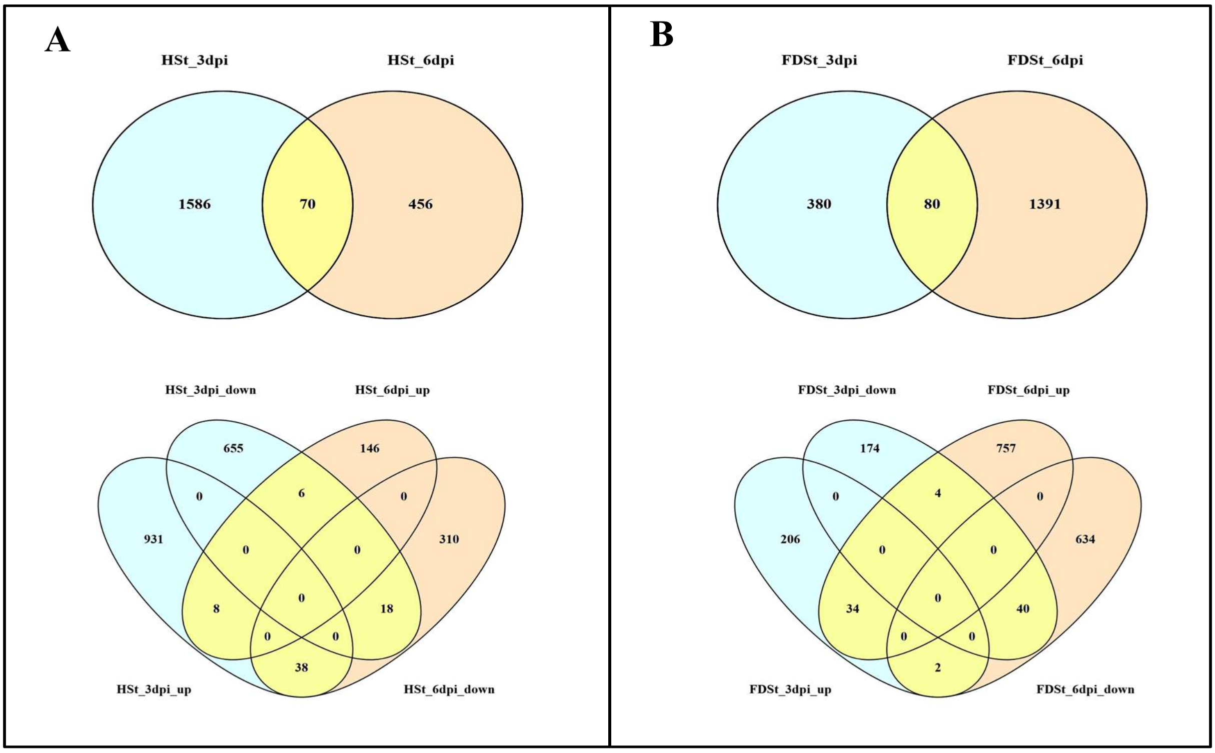

Venn diagrams of genes modulated after HSt and FDSt attacks in Chardonnay at two time points. Venn diagrams illustrating the relationship between the DEGs modulated after HSt and FDSt attacks at 3 (**A on the top**) and 6 days post infestation (**B on the top**) and the overlap between up-regulated and down-regulated DEGs (**A and B on the bottom**).

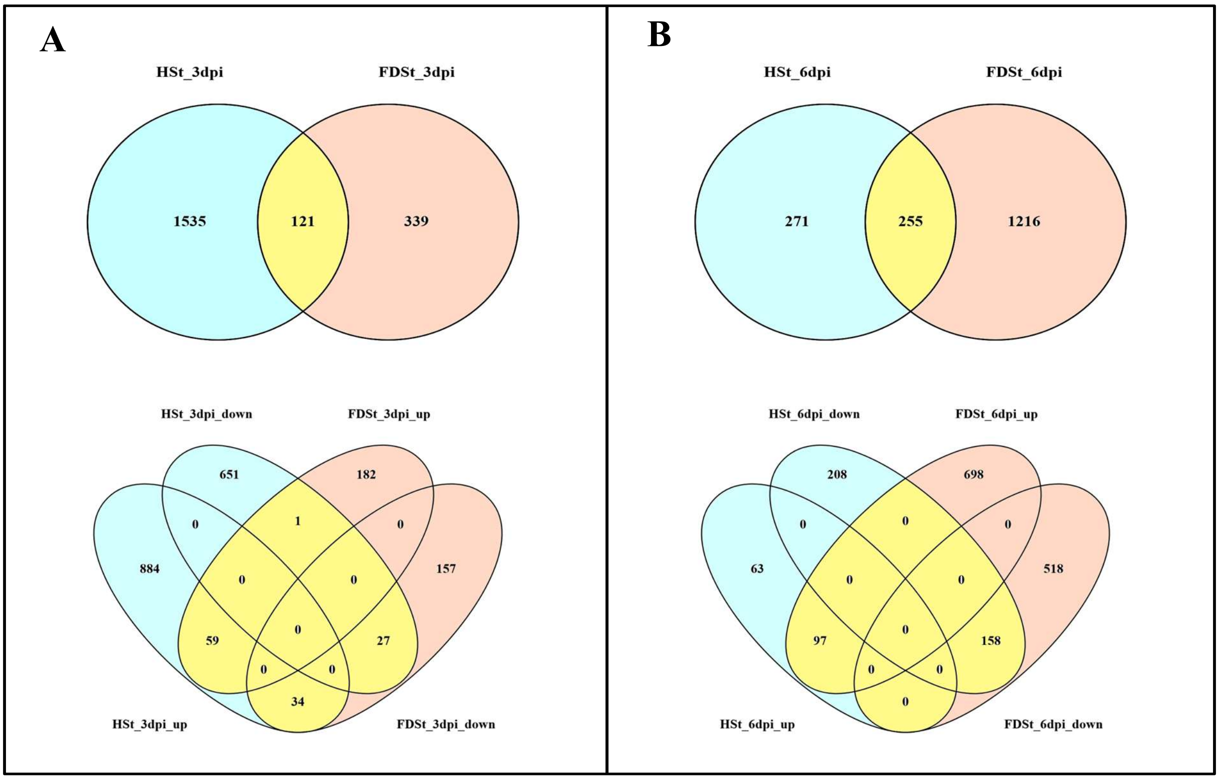

4) Venn diagrams of genes modulated at 3 and 6 days after HSt and FDSt attacks in Tocai friulano. Venn diagrams illustrating the relationship between the DEGs detected at 3 and 6 days post infestation in HSt (**A on the top**) and FDSt (**B on the top**) and the overlap between up-regulated and down-regulated DEGs (**A and B on the bottom**).

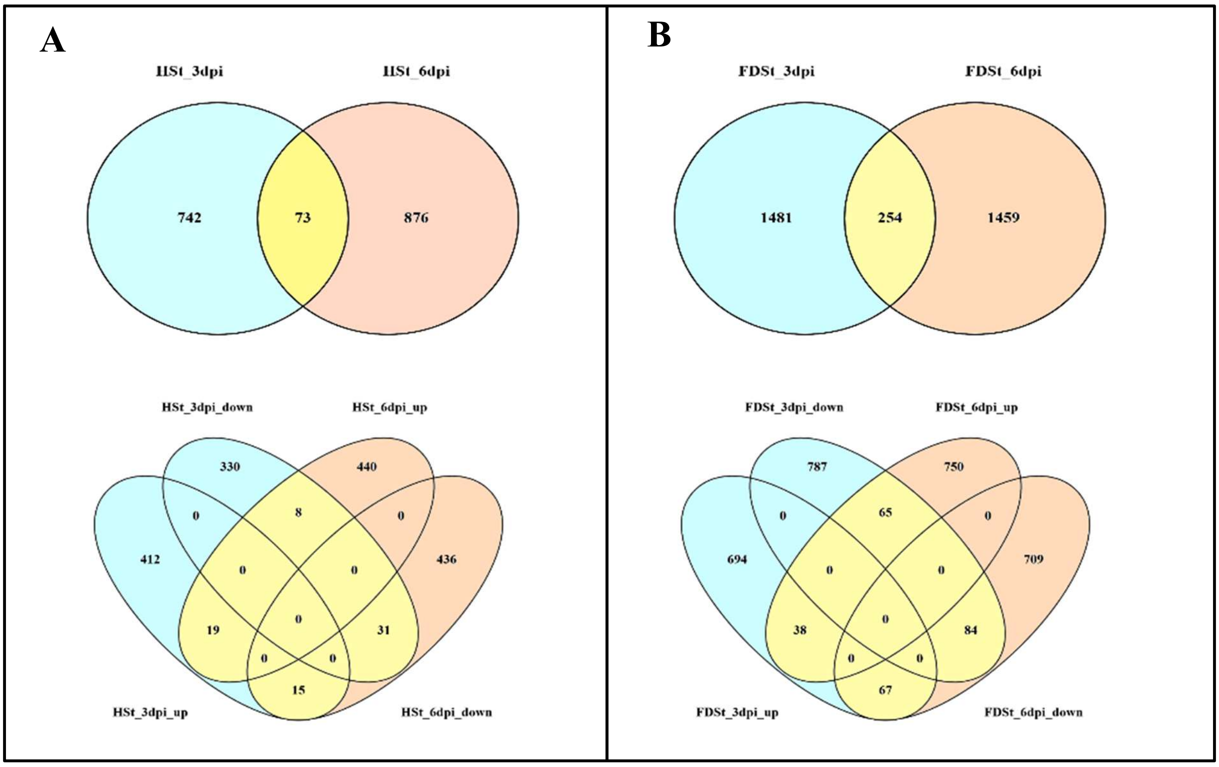

5) Venn diagrams of genes modulated after HSt and FDSt attacks in Tocai friulano at two time points. Venn diagrams illustrating the relationship between the DEGs modulated after HSt and FDSt attacks at 3 (**A on the top**) and 6 days post infestation (**B on the top**) and the overlap between up-regulated and down-regulated DEGs (**A and B on the bottom**).

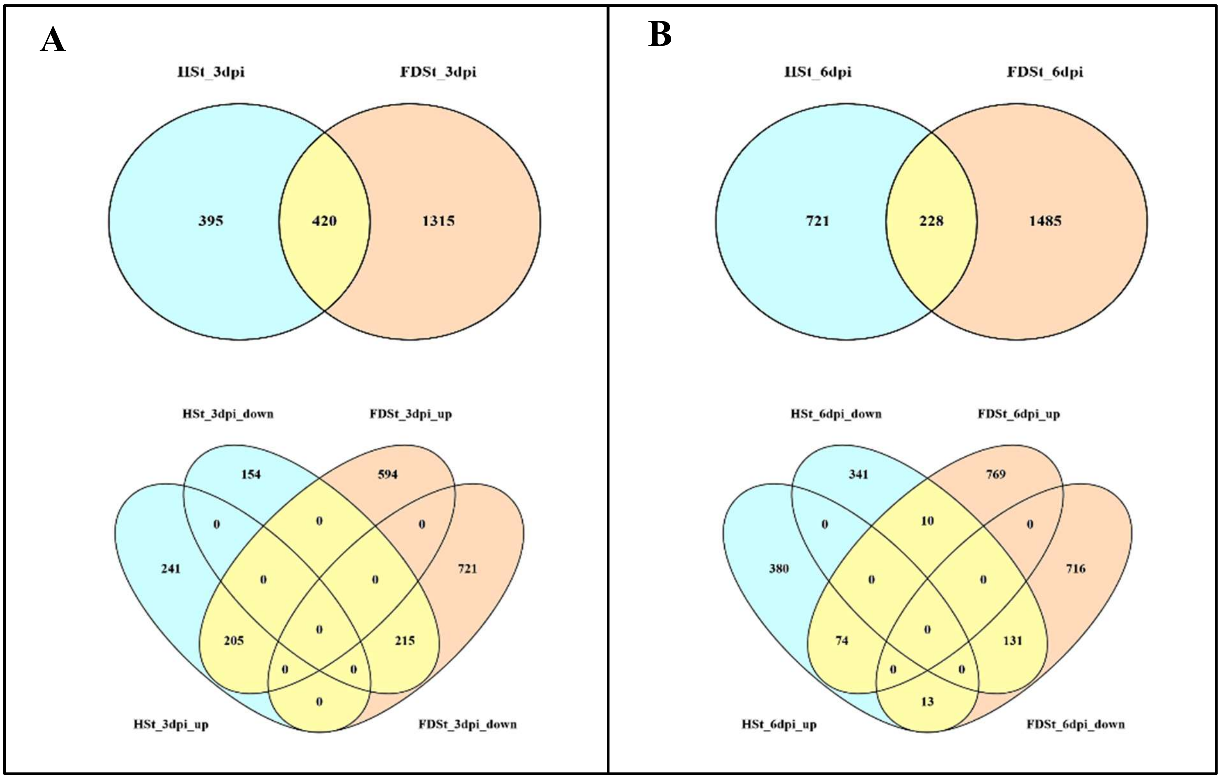

Supplement: Supplementary file 17 — Venn diagrams illustrating the relationship between the DEGs in the different treatments and the overlap between up-regulated and down-regulated DEGs. Twenty Venn diagrams are reported, grouped in five different figures; each figure includes two pairwise comparisons on the top, and the overlap between up-regulated and down-regulated DEGs on the bottom. (PDF 791 kb) [file 12864_2019_5908_MOESM17_ESM.pdf]
