# Supplementary material for: Grapevine comparative early transcriptomic profiling suggests that Flavescence dorée phytoplasma represses plant responses induced by vector feeding in susceptible varieties
Source: BMC Genomics. 2019 Jun 26;20:526. doi: 10.1186/s12864-019-5908-6 (PMC6595628; doi:10.1186/s12864-019-5908-6)

A) Signaling pathways

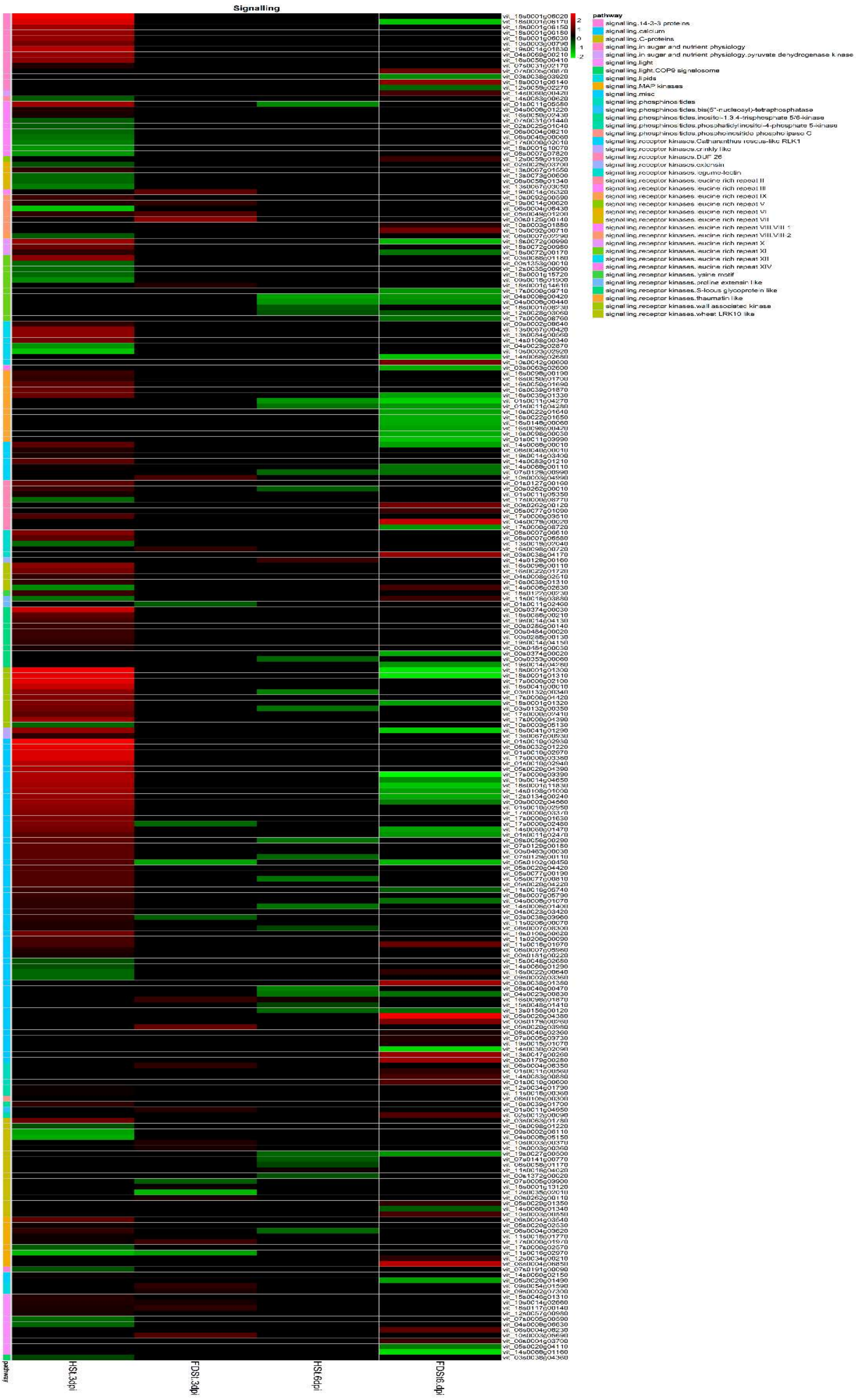

B) Hormone-related pathways

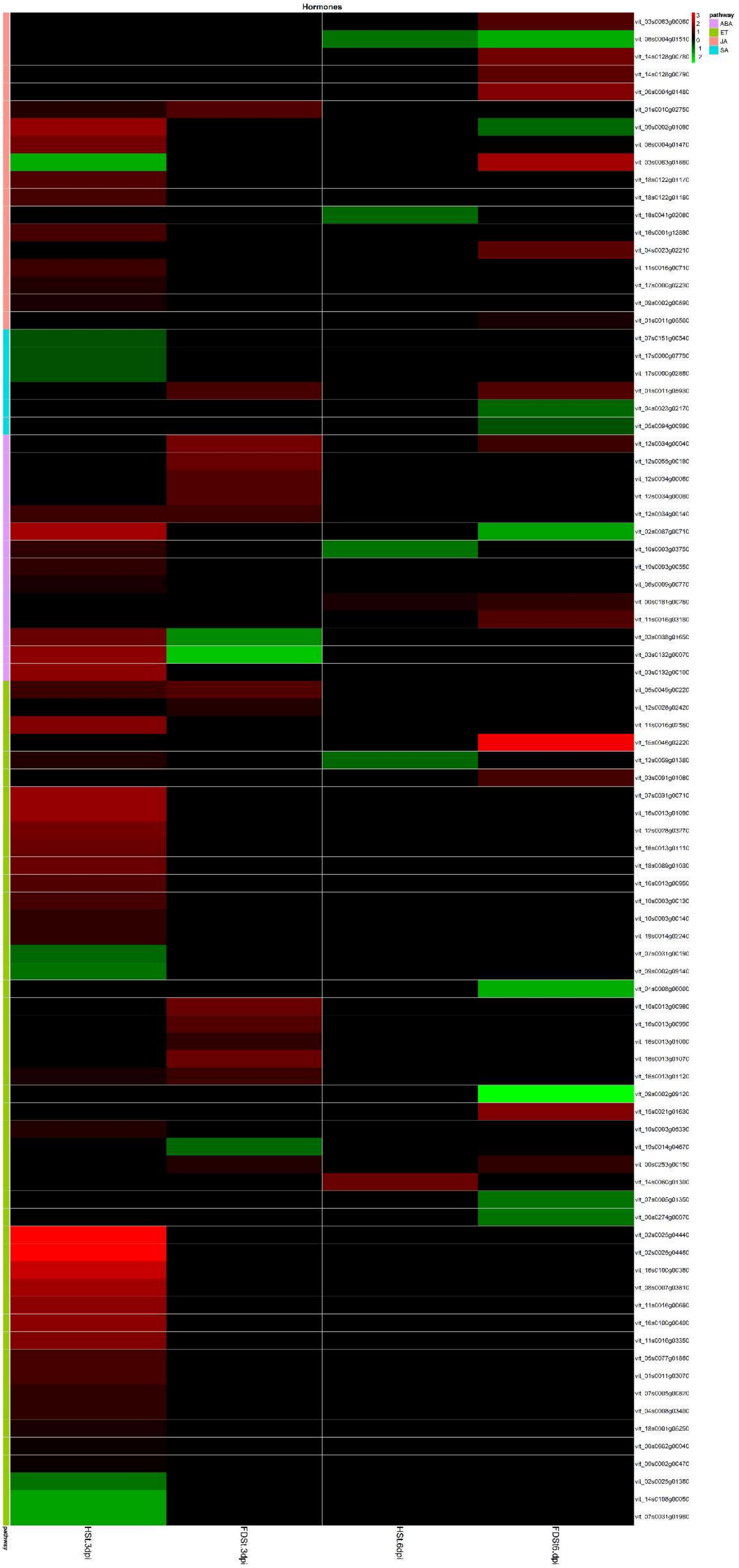

C) Transcription factors

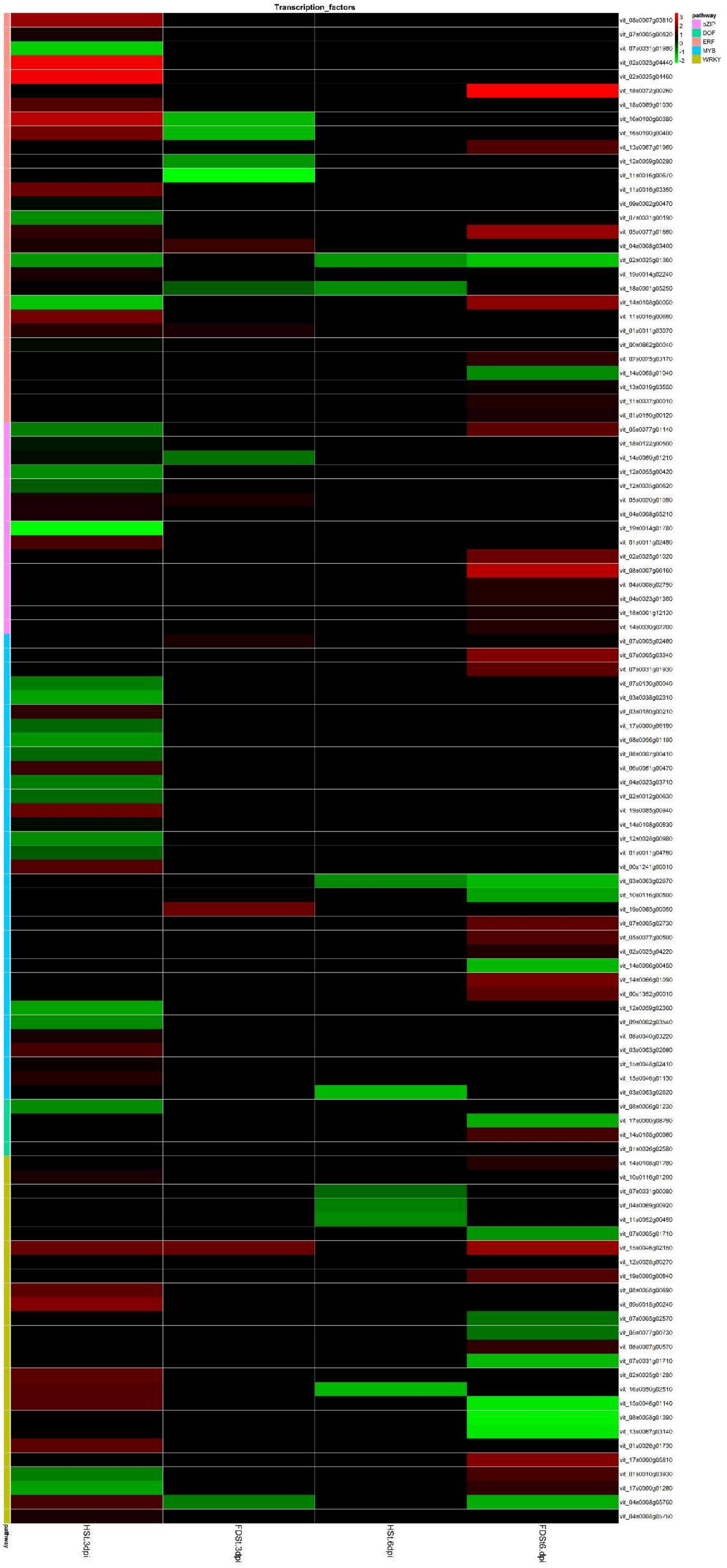

D) Antioxidant mechanisms

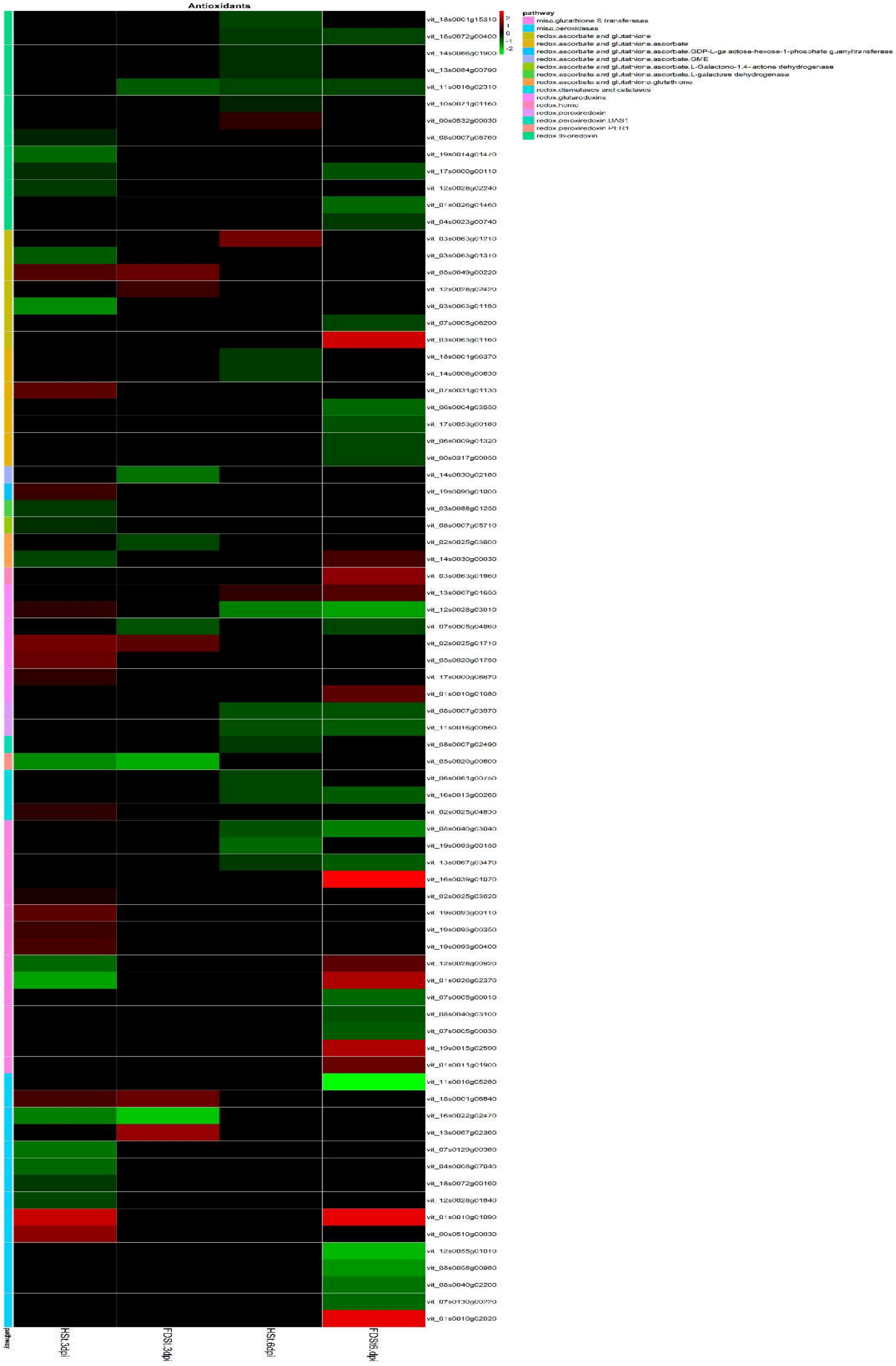

E) Cell wall

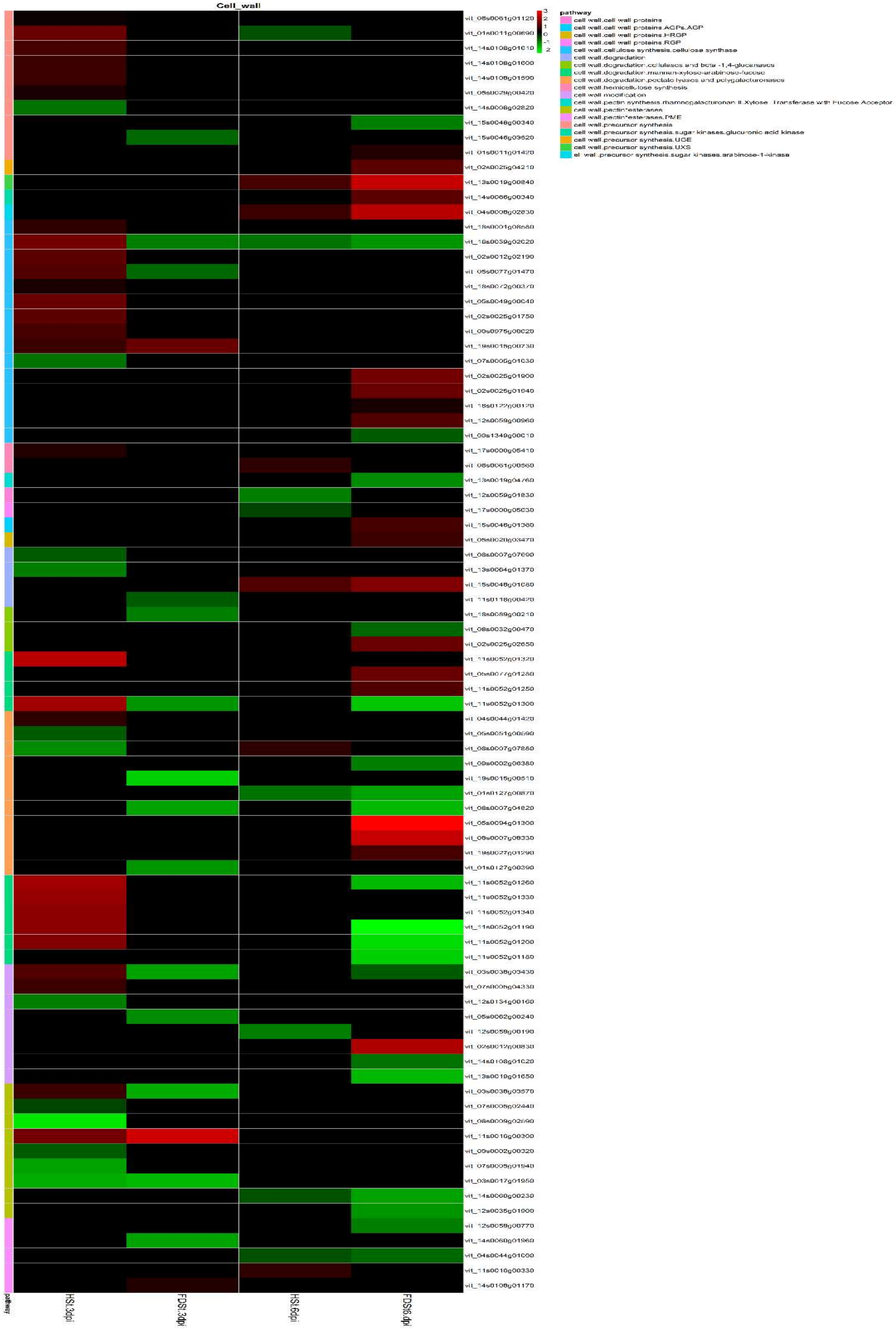

Cell_wall
pathway
cell wall.cell wall proteins
cell wall.cell wall proteins.AGPs.AGP
cell wall.cell wall proteins.HRGP
cell wall.cell wall proteins.RGP
cell wall.cellulose synthesis.cellulose synthase
cell wall.degradation
cell wall.degradation.cellulases and beta -1,4-glucanases
cell wall.degradation.mannan-xylose-arabinose-fucose
cell wall.degradation.pectate lyases and polygalacturonases
cell wall.hemicellulose synthesis
cell wall.modification
cell wall.pectin synthesis.rhamnogalacturonan II.Xylose Transferase with Fucose Acceptor
cell wall.pectin*esterases
cell wall.pectin*esterases.PME
cell wall.precursor synthesis
cell wall.precursor synthesis.sugar kinases.glucuronic acid kinase
cell wall.precursor synthesis.UGE
cell wall.precursor synthesis.UXS
el wal .precursor synthesis.sugar kinases.arabinose-1-kinase
3
2
1
0
-1
2
pathway
HSt.3dpi
FDSt.3dpi
HSt.6dpi
FDSt6.dpi

F) Defense proteins

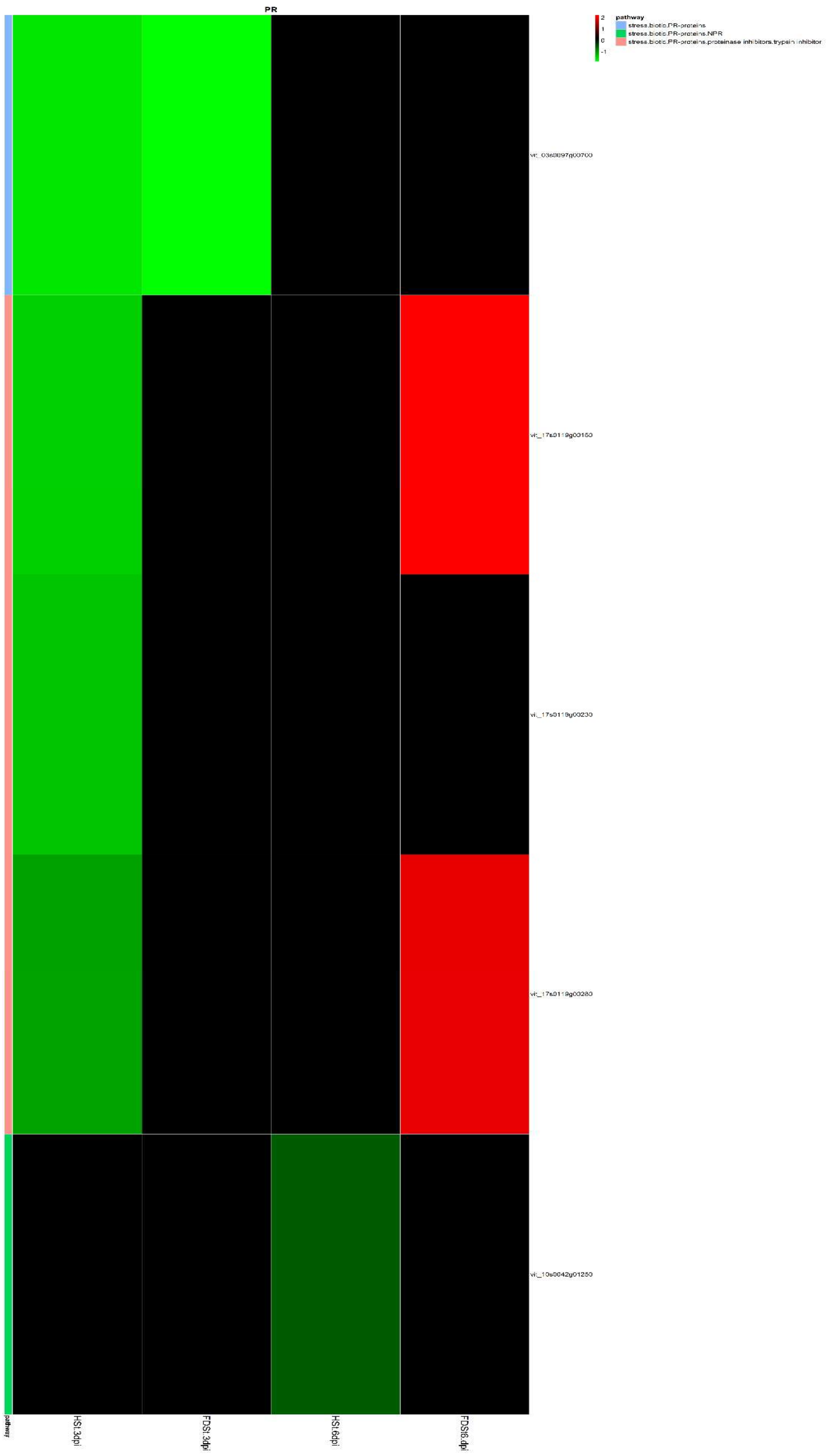

G) Secondary metabolism

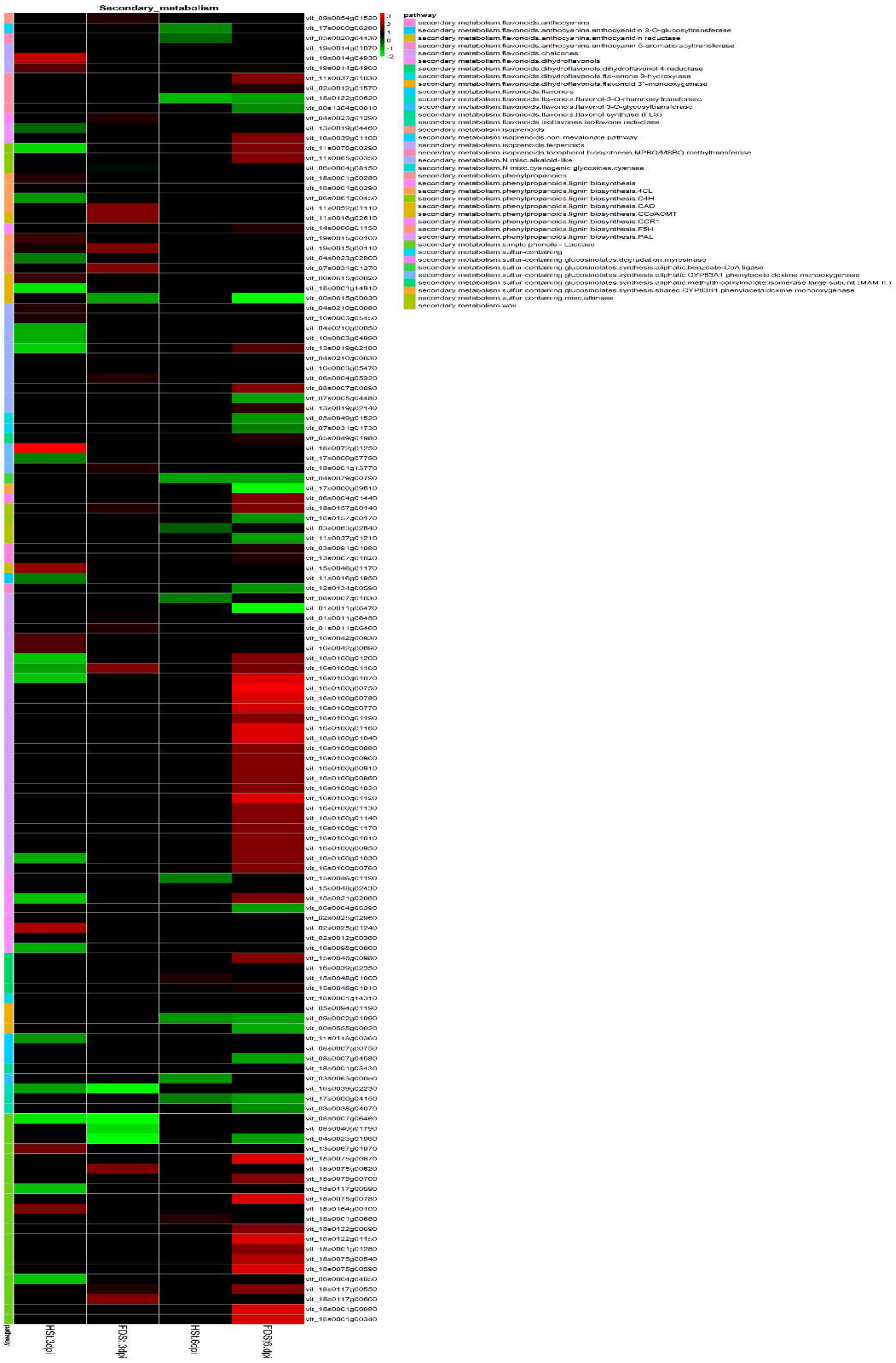

H) Primary metabolism

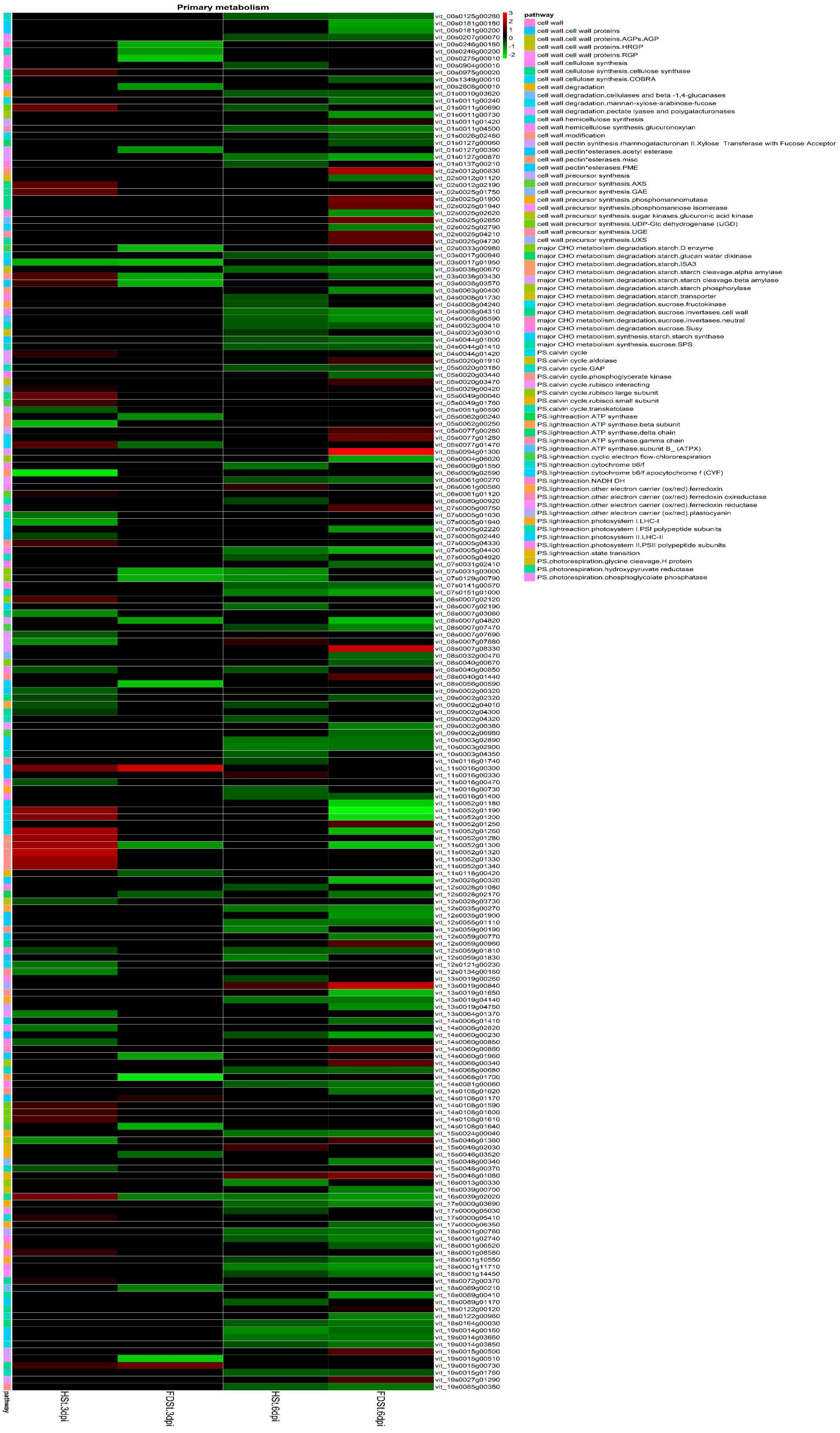

Supplement: Supplementary file 22 — Expression patterns of genes involved in different pathways in Chardonnay: A) signaling pathways, B) hormone-related pathways, C) transcription factors, D) antioxidant mechanisms, E) cell wall, F) defense proteins, G) secondary metabolism, H) primary metabolism. From left to right, the four columns show the expression of DEGs modulated by HSt and FDSt at 3 and 6 days post infestation, respectively. Every row represents a different gene. The green, black and red colors indicate the log2 fold changes, low, medium and high, respectively, obtained from DESeq2 with FDR < 0.05. Log2 fold changes of genes that were not considered differentially regulated in a specific comparison due to a FDR above the threshold were arbitrary set to 0. (PDF 2356 kb) [file 12864_2019_5908_MOESM22_ESM.pdf]
