# Supplementary material for: Grapevine comparative early transcriptomic profiling suggests that Flavescence dorée phytoplasma represses plant responses induced by vector feeding in susceptible varieties
Source: BMC Genomics. 2019 Jun 26;20:526. doi: 10.1186/s12864-019-5908-6 (PMC6595628; doi:10.1186/s12864-019-5908-6)

A) Signaling pathways

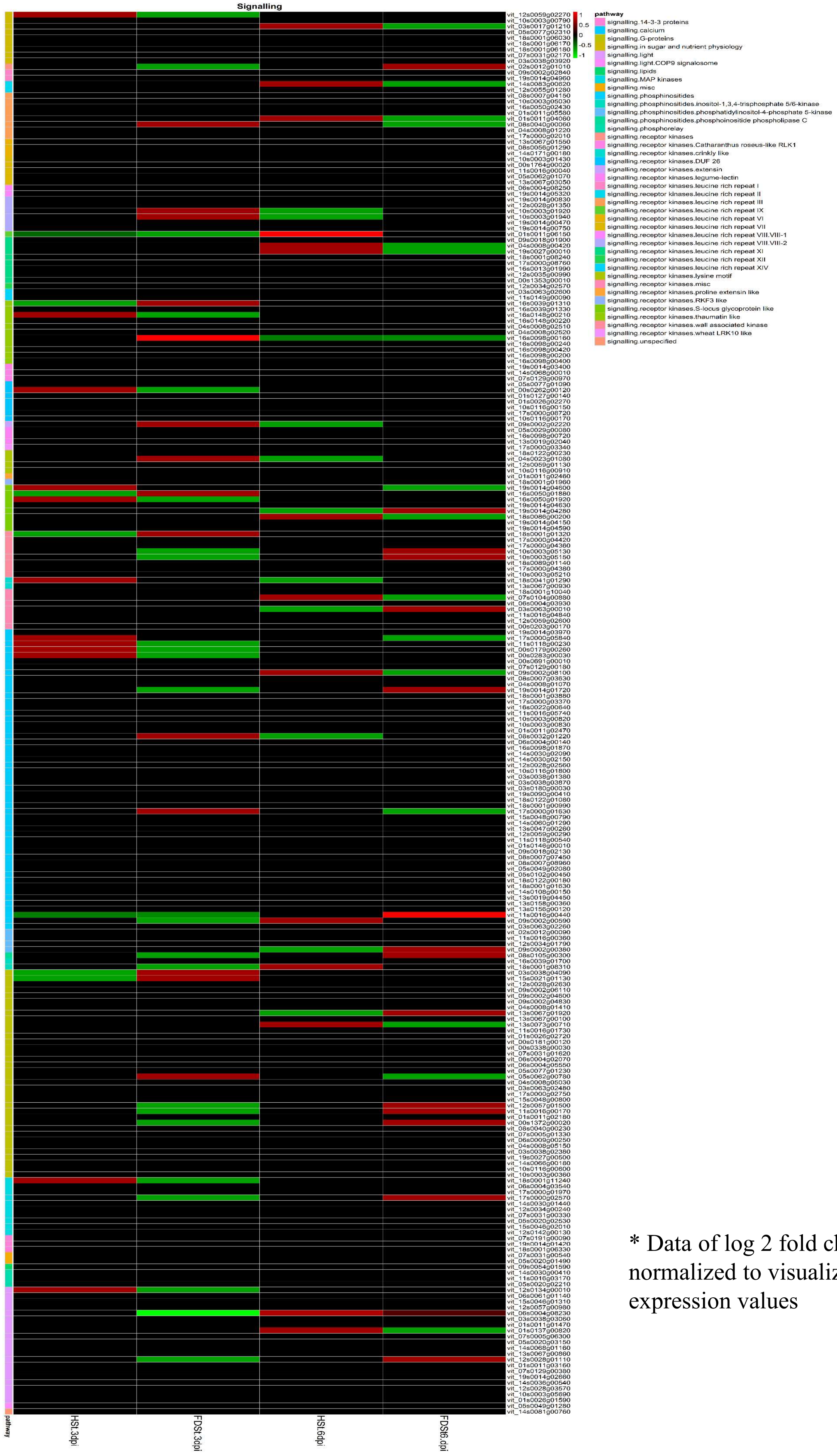

* Data of log 2 fold change are normalized to visualize all the expression values

B) Hormone-related pathways

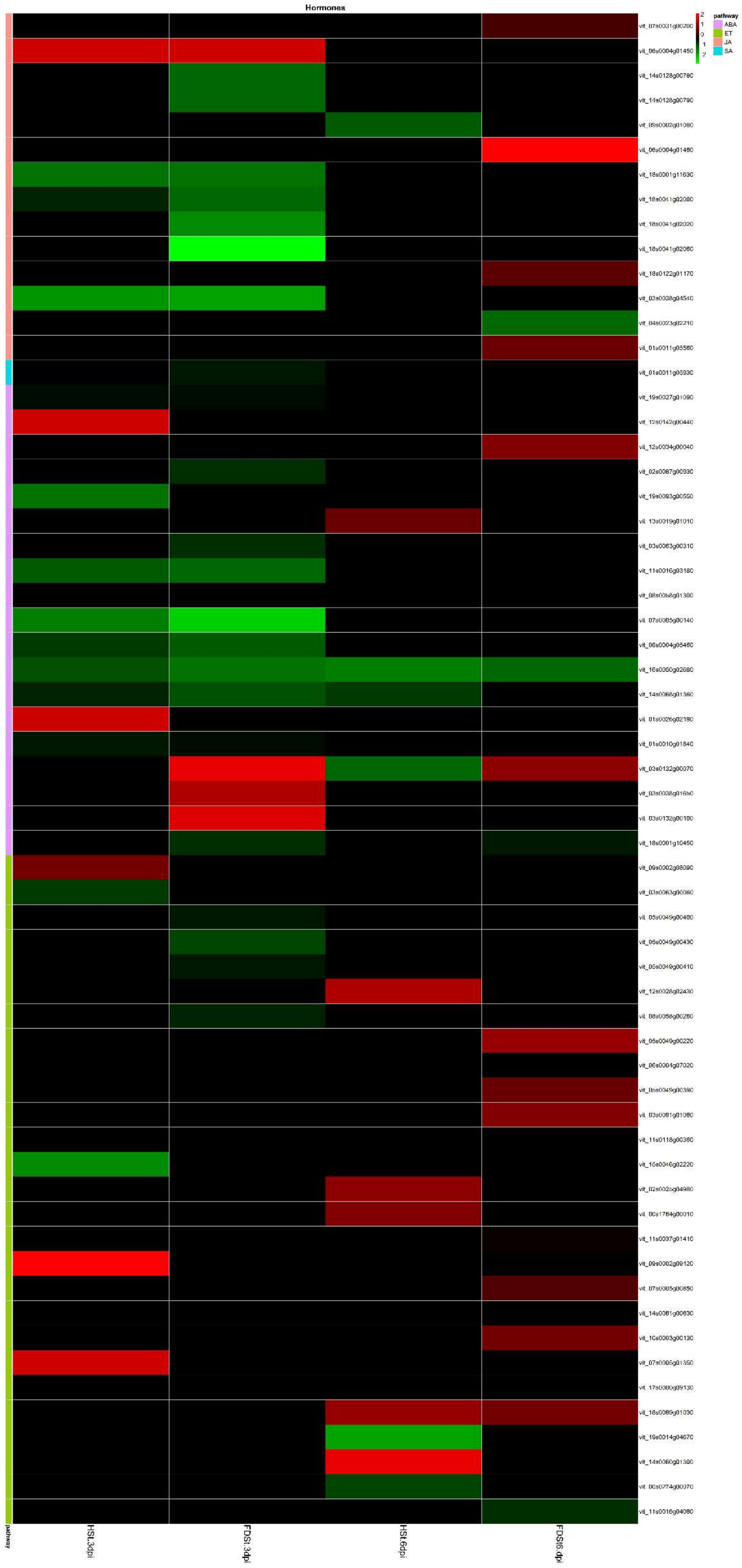

C) Transcription factors

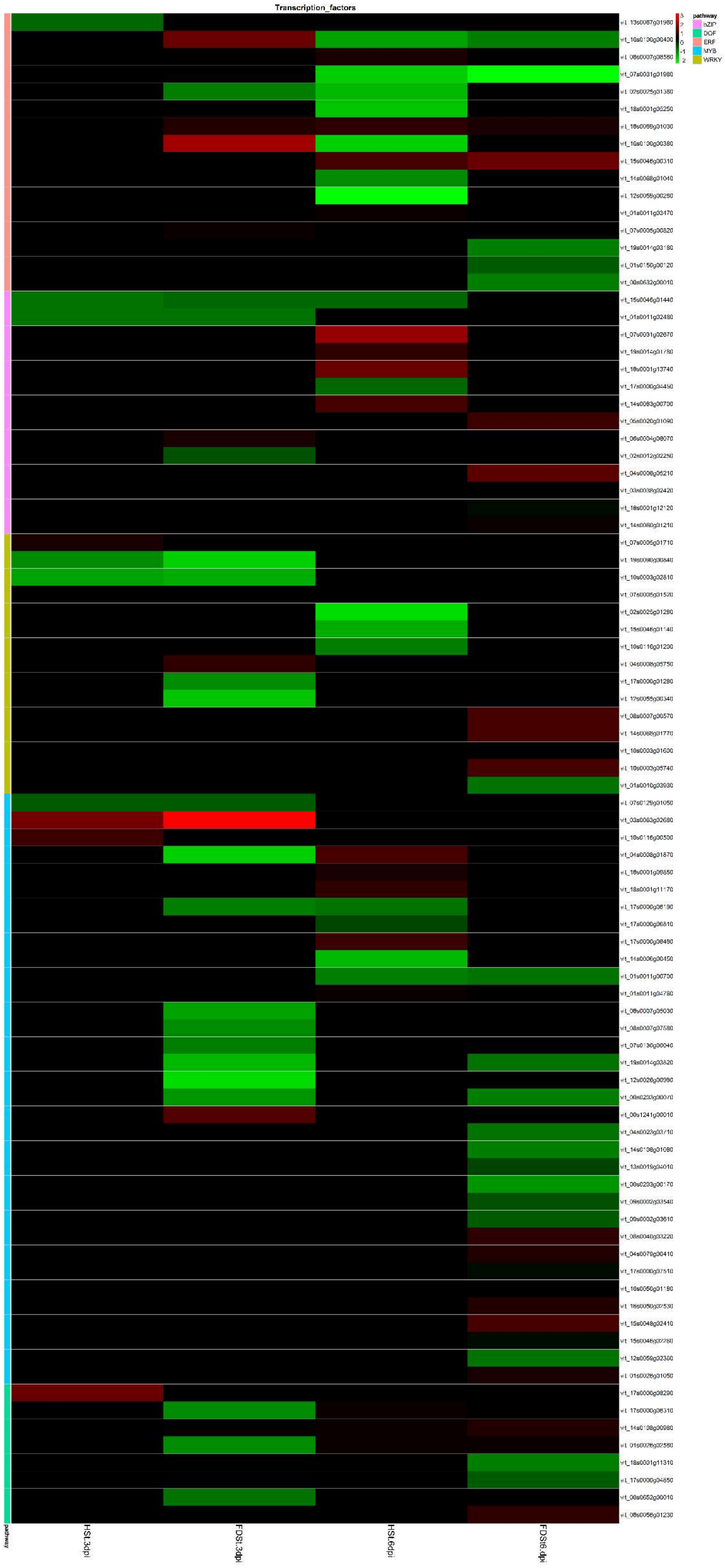

D) Antioxidant mechanisms

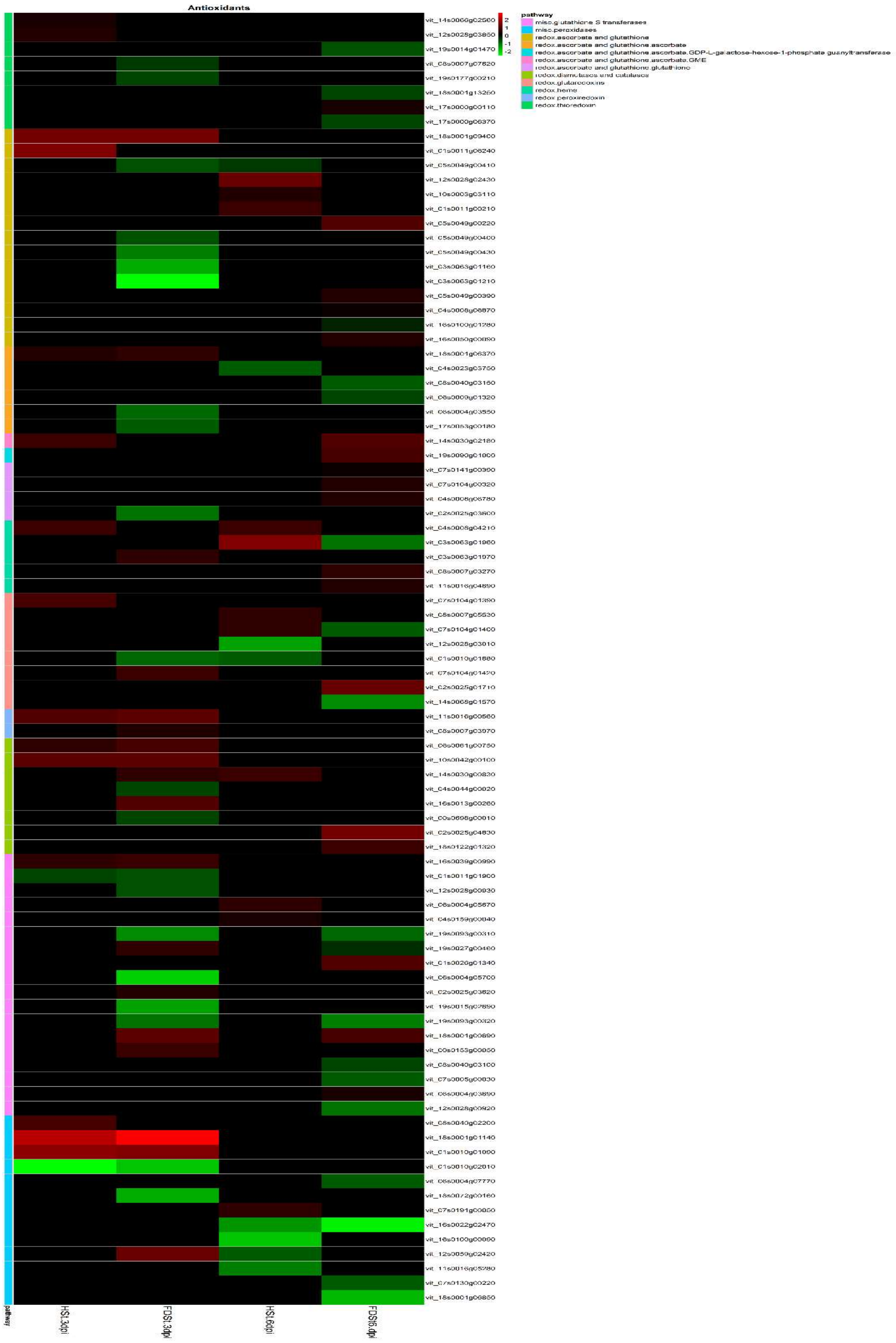

E) Cell wall

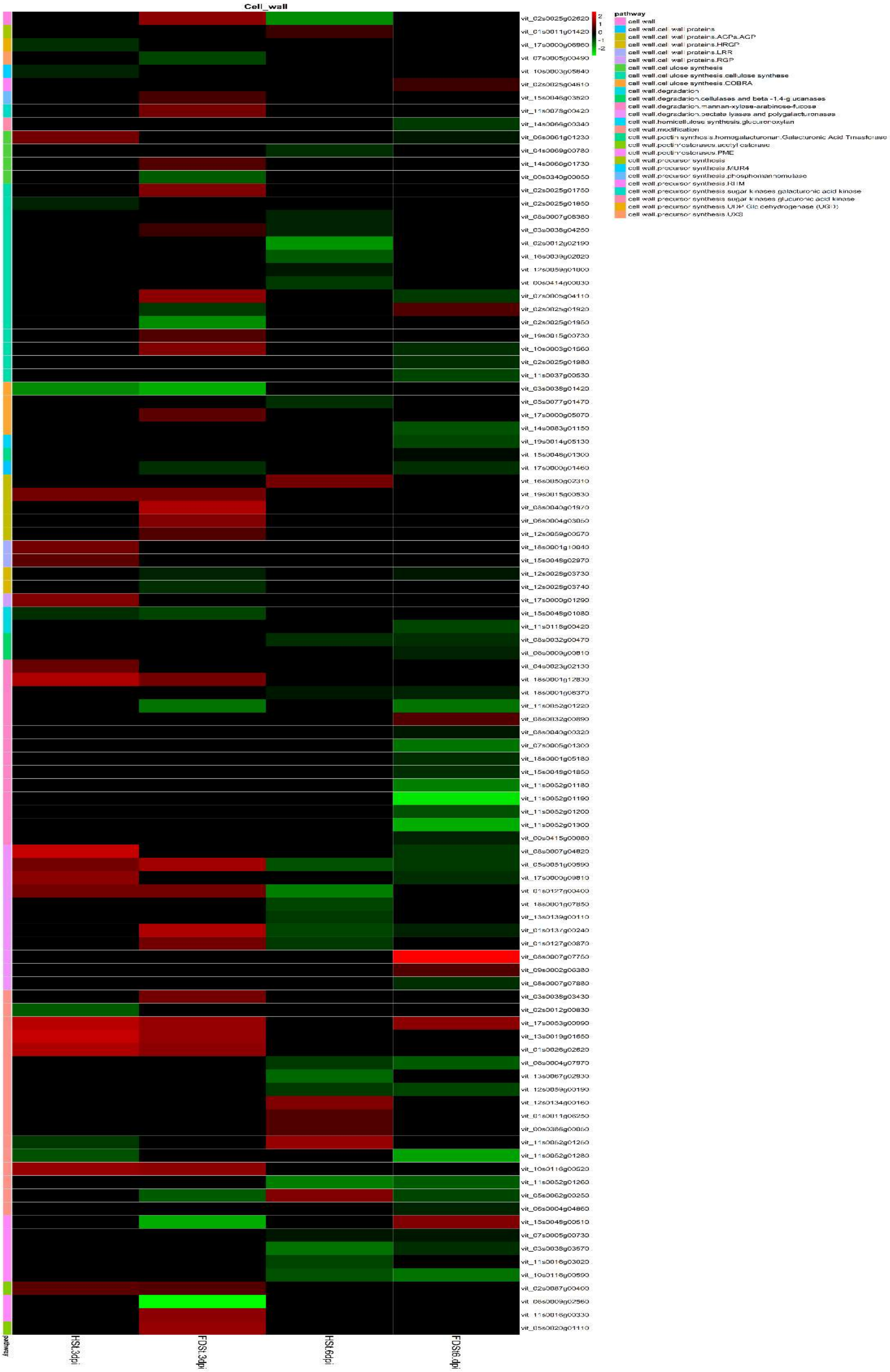
Cell_wall
vit_02s0025g02620
vit_01s0011g01420
vit_17s0000g06960
vit_07s0005g00490
vit_10s0003g05840
vit_02s0025g04610
vit_15s0046g03520
vit_11s0078g00420
vit_14s0066g00340
vit_06s0061g01230
vit_04s0069g00780
vit_14s0066g01730
vit_00s0340g00050
vit_02s0025g01750
vit_02s0025g01850
vit_08s0007g08380
vit_03s0038g04250
vit_02s0012g02190
vit_16s0039g02020
vit_12s0059g01000
vit_00s0414g00030
vit_07s0005g04110
vit_02s0025g01920
vit_02s0025g01950
vit_19s0015g00730
vit_10s0003g01560
vit_02s0025g01980
vit_11s0037g00530
vit_03s0038g01420
vit_05s0077g01470
vit_17s0000g05070
vit_14s0083g01150
vit_19s0014g05130
vit_15s0046g01300
vit_17s0000g01460
vit_16s0050g02310
vit_19s0015g00530
vit_08s0040g01970
vit_06s0004g03050
vit_12s0059g00570
vit_18s0001g10040
vit_15s0048g02970
vit_12s0028g03730
vit_12s0028g03740
vit_17s0000g01290
vit_15s0048g01080
vit_11s0118g00420
vit_08s0032g00470
vit_06s0009g00810
vit_04s0023g02130
vit_18s0001g12830
vit_18s0001g08370
vit_11s0052g01220
vit_08s0032g00890
vit_08s0040g00320
vit_07s0005g01300
vit_18s0001g05180
vit_15s0048g01850
vit_11s0052g01180
vit_11s0052g01190
vit_11s0052g01200
vit_11s0052g01300
vit_00s0415g00080
vit_08s0007g04820
vit_05s0051g00590
vit_17s0000g09810
vit_01s0127g00400
vit_18s0001g07850
vit_13s0139g00110
vit_01s0137g00240
vit_01s0127g00870
vit_08s0007g07750
vit_09s0002g06380
vit_08s0007g07880
vit_03s0038g03430
vit_02s0012g00830
vit_17s0053g00990
vit_13s0019g01650
vit_01s0026g02620
vit_06s0004g07970
vit_13s0067g02930
vit_12s0059g00190
vit_12s0134g00160
vit_01s0011g06250
vit_00s0386g00050
vit_11s0052g01250
vit_11s0052g01280
vit_10s0116g00520
vit_11s0052g01260
vit_05s0062g00250
vit_06s0004g04860
vit_15s0048g00510
vit_07s0005g00730
vit_03s0038g03570
vit_11s0016g03020
vit_10s0116g00590
vit_02s0087g00400
vit_06s0009g02560
vit_11s0016g00330
vit_05s0020g01110
pathway
HS1.3dpi
FDS1.3dpi
HS1.6dpi
FDS16.dpi
2
1
0
-1
-2
pathway
cell wall
cell wall.cell wall proteins
cell wall.cell wall proteins.AGPs.AGP
cell wall.cell wall proteins.HRGP
cell wall.cell wall proteins.LRR
cell wall.cell wall proteins.RGP
cell wall.cellulose synthesis
cell wall.cellulose synthesis.cellulose synthase
cell wall.cellulose synthesis.COBRA
cell wall.degradation
cell wall.degradation.cellulases and beta -1.4-glucanases
cell wall.degradation.mannan-xylose-arabinose-fucose
cell wall.degradation.pectate lyases and polygalacturonases
cell wall.hemicellulose synthesis.glucuronoxylan
cell wall.modification
cell wall.pectin synthesis.homogalacturonan.Galacturonic Acid Tranasferase
cell wall.pectin*esterases.acetyl esterase
cell wall.pectin*esterases.PME
cell wall.precursor synthesis
cell wall.precursor synthesis.MUR4
cell wall.precursor synthesis.phosphomannomutase
cell wall.precursor synthesis.RHM
cell wall.precursor synthesis.sugar kinases.galacturonic acid kinase
cell wall.precursor synthesis.sugar kinases.glucuronic acid kinase
cell wall.precursor synthesis.UDP Glc dehydrogenase (UGD)
cell wall.precursor synthesis.UXS

F) Defense proteins

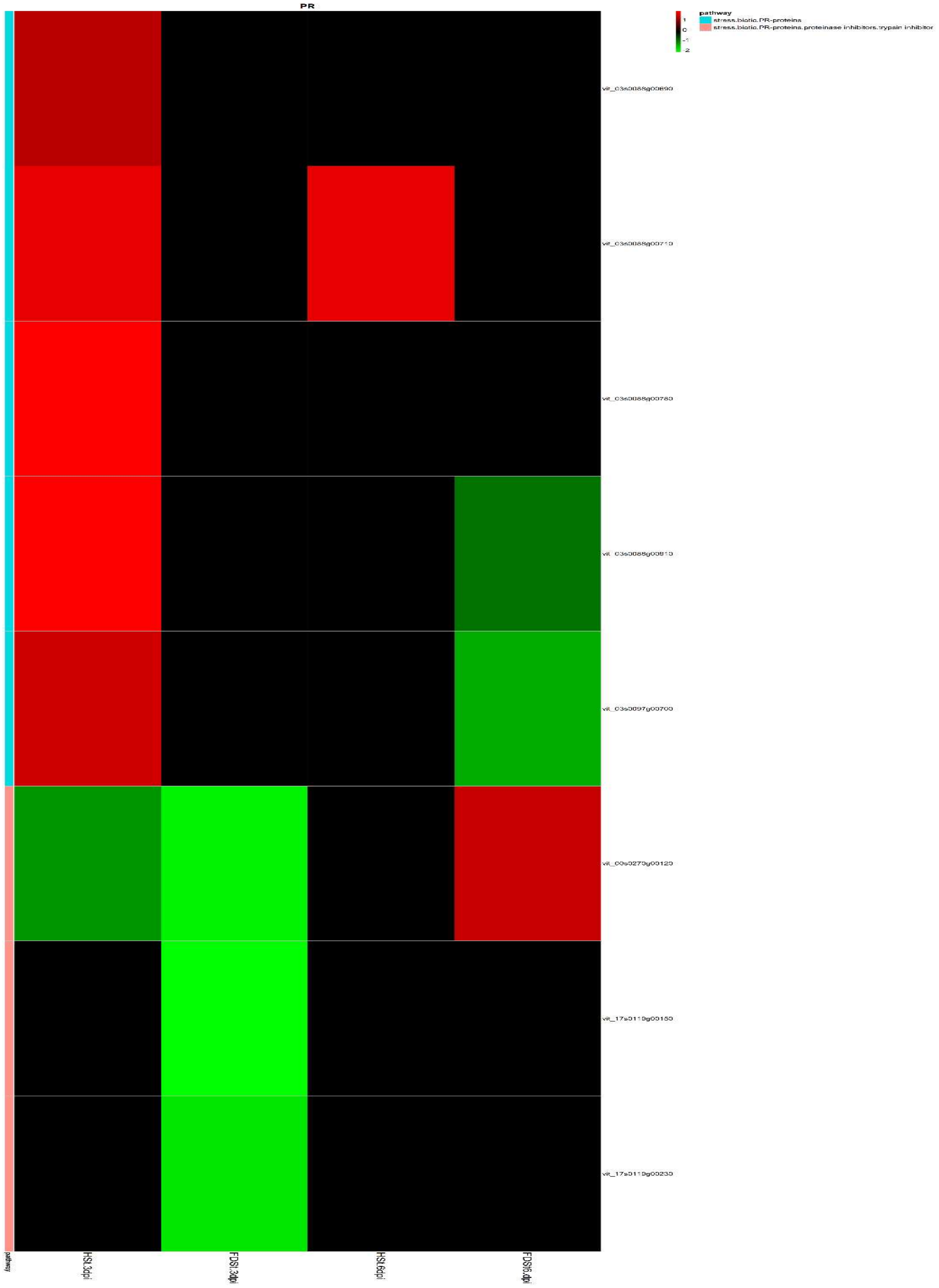

G) Secondary metabolism

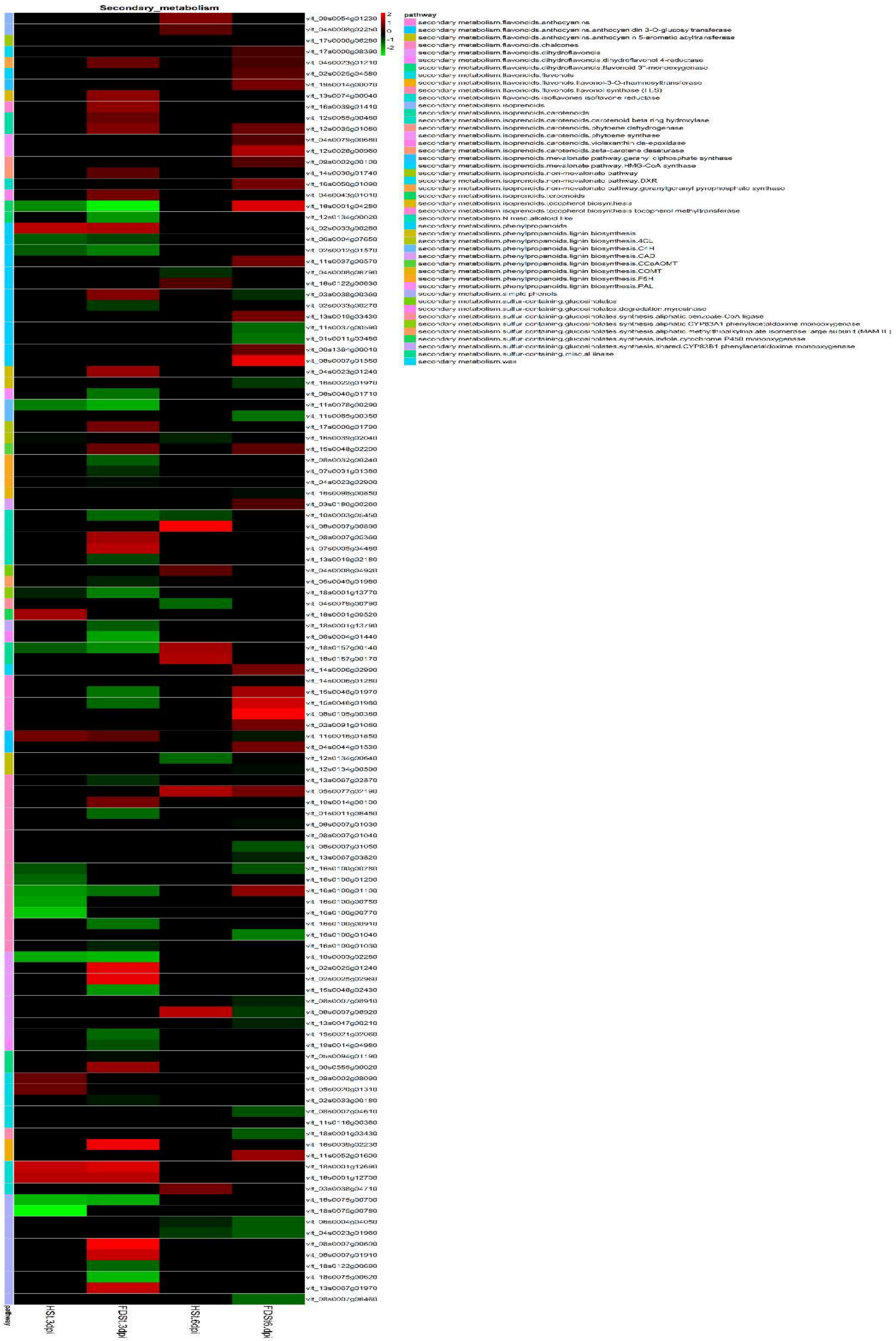

H) Primary metabolism

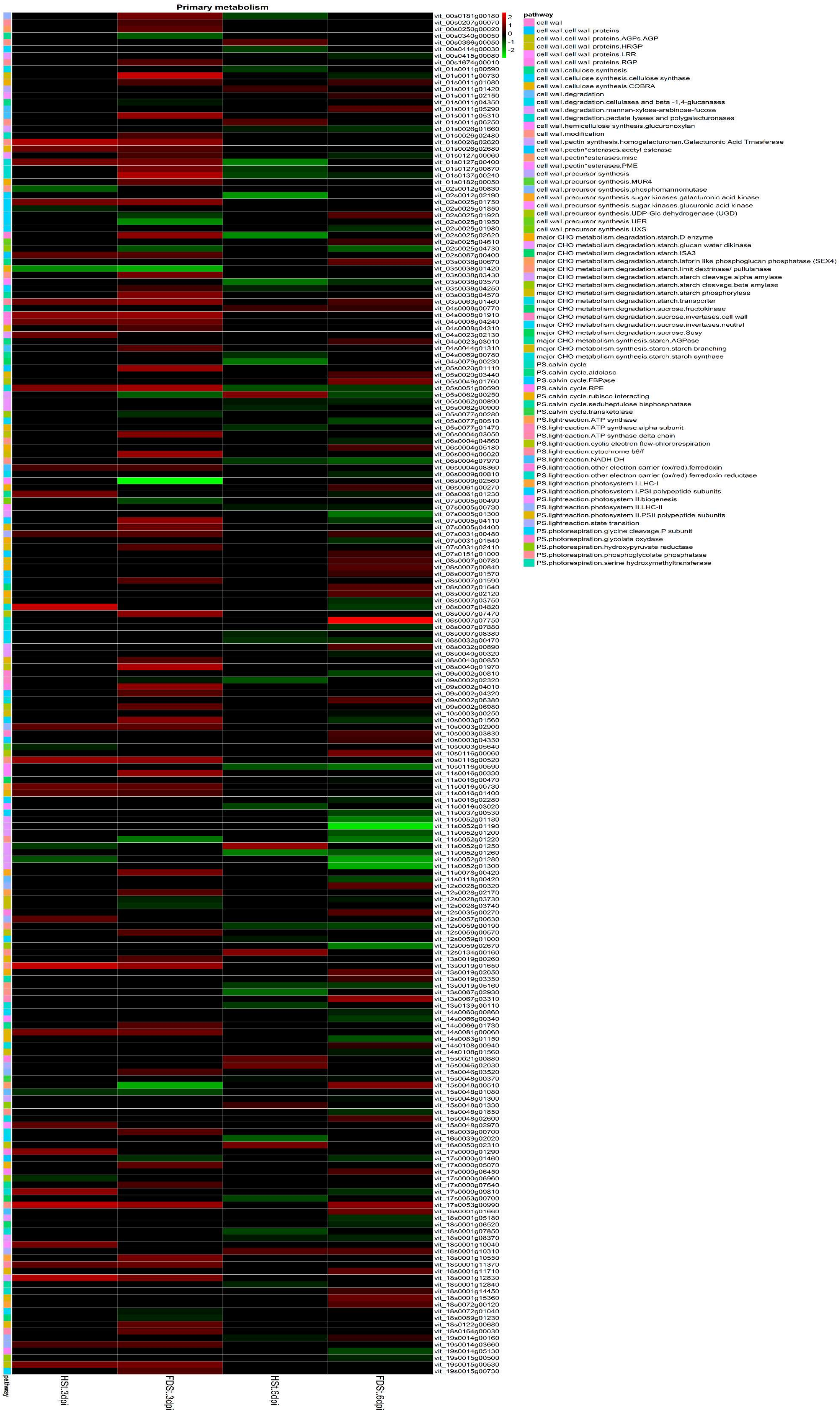

Supplement: Supplementary file 23 — Expression patterns of genes involved in different pathways in Tocai friulano: A) signaling pathways, B) hormone-related pathways, C) transcription factors, D) antioxidant mechanisms, E) cell wall, F) defense proteins, G) secondary metabolism, H) primary metabolism. From left to right, the four columns respectively show the expression of DEGs modulated by HSt and FDSt at 3 and 6 days post infestation. Every row represents a different gene. The green, black and red colors indicate the log2 fold changes, respectively low, medium and high, obtained from DESeq2 with FDR < 0.05. For genes that were not considered differentially regulated in a specific comparison due to a FDR above the threshold, their log2 fold change have been arbitrary set to 0. (PDF 2755 kb) [file 12864_2019_5908_MOESM23_ESM.pdf]
